# Semisynthesis and anti-cancer properties of novel honokiol derivatives in human nasopharyngeal carcinoma CNE-2Z cells

**DOI:** 10.1080/14756366.2023.2244694

**Published:** 2023-08-09

**Authors:** Bo-Han Li, Hui Ma, Jing Zhu, Jie Chen, Yi-Qun Dai, Xiao-Jing Zhang, Hong-Mei Li, Cheng-Zhu Wu

**Affiliations:** a School of Pharmacy, Bengbu Medical College, Bengbu, Anhui, China; b Anhui Province Biochemical Pharmaceutical Engineering Technology Research Center, Bengbu, Anhui, China; c Department of Surgical Oncology, The First Affiliated Hospital of Bengbu Medical College, Bengbu, Anhui, China

**Keywords:** Honokiol derivatives, nasopharyngeal carcinoma, migration, invasion, HIF-1α

## Abstract

In this study, 21 new honokiol derivatives were synthesised, and their anti-cancer properties were investigated. Among these, compound **1g** exhibited the most potent cytotoxic activity against human nasopharyngeal carcinoma CNE-2Z cells, human gastric cancer SGC7901 cells, human breast cancer MCF-7 cells, and mouse leydig testicular cancer I-10 lines with IC_50_ values of 6.04, 7.17, 6.83, and 5.30 μM, respectively. Compared to the parental compound, **1g** displayed up to 5.18-fold enhancement of the cytotoxic effect on CNE-2Z cells. We further demonstrated that **1g** inhibited cell growth, suppressed migration and invasion, and induced apoptosis of CNE-2Z cells by down-regulating HIF-1α, MMP-2, MMP-9, Bcl-2, Akt and up-regulating Bax protein levels. Transfection of CNE-2Z cells with HIF-1α siRNA reduced cell migration and invasion. In addition, *in vivo* experiments confirmed that **1g** inhibited tumour growth in CNE-2Z cell-xenografted nude mice with low toxicity. Thus, our data suggested that **1g** was a potent and safe lead compound for nasopharyngeal carcinoma therapy.

## GRAPHICAL ABSTRACT

**Figure:**
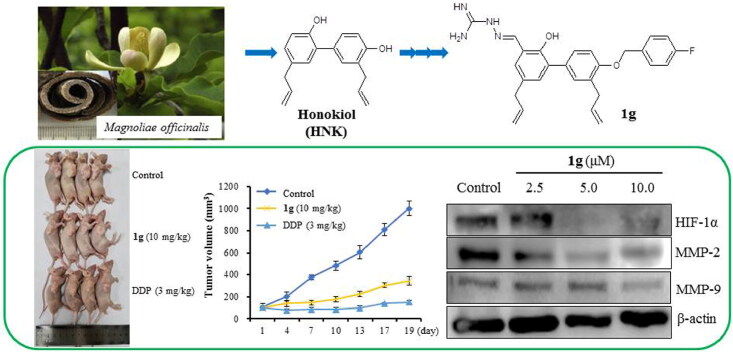

## Introduction

Cancer is one of the leading causes of death, and it is a critical issue in the worldwide. The International Agency for Research on Cancer (IARC) estimated that there were 19.3 million new cancer cases and approximately 10 million cancer-related deaths in 2020.^1^ In Southeast Asia, North Africa, and Southern China, nasopharyngeal carcinoma (NPC) is one of the common malignant tumours of the head and neck, which endangered human health.^2^^,^^3^ Although most patients with NPC respond well to radiotherapy/chemotherapy, with an improved 5-year survival rate of approximately 80%, local recurrence and distant metastasis are still primarily the causes of treatment failure and death associated with NPC.^4^^,^^5^ Therefore, it is necessary to discover new therapeutic targets and effective agents to prevent the invasion and metastasis of NPC.

Hypoxia is a common characteristic of most solid tumours, including NPC.^6^ Hypoxia-inducible factor-1α (HIF-1α) is a potential therapeutic target for many types of cancer.^7^^,^^8^ Recently, several studies have provided convincing evidence for a strong correlation between increased HIF-1α levels and tumour metastasis, angiogenesis, poor patient prognosis, and cancer drug resistance.^9–12^ In addition, meta-analysis demonstrated that HIF-1α could be an appropriate prognostic biomarker for NPC patients.^13^

Natural products play an important role in drug discovery and research.^14^^,^^15^ Currently, approximately 80% of small molecule anti-cancer agents on the market are originate natural products or their modified derivatives.^16^ Honokiol (HNK), a biphenyl neolignan extracted from *Magnolia* species, exhibited several pharmacological effects, including anti-cancer, anti-inflammatory, anti-oxidant, anti- infectious, and neuroprotective activities.^17^^,^^18^ These findings promote interest in the application of HNK and its analogs in the treatment of cancer, including anti-proliferation, induction of programmed cell death, suppression of invasion and metastasis, and inhibition of angiogenesis.^19–22^ Many studies have demonstrated that the combination of HNK with other anti-cancer agents or ionising radiation could generate better therapeutic effects.^23–25^ This evidence suggests that HNK could serve as a potential lead compound candidate for the further development and application of anti-cancer drugs.

Previous studies have reported that a series of HNK derivatives exhibited higher cytotoxic activity than HNK.^26–30^ These studies indicated that free phenolic hydroxyl groups and hydrophobic side chains were necessary active groups for HNK to exert anti-cancer properties. Aminoguanidine is a functional moiety with high polarity and the capacity for hydrogen bonding with critical amino acid residues and metal ions.^31^ Aminoguanidine derivatives (e.g. metformin) have been the focus of many studies because of their diverse pharmacolgical properties, including anti-cancer, anti-microbial, and anti-inflammatory effects.^32–35^ In the present study, we modified the phenolic hydroxyl group of HNK and preserved the aminoguanidine moiety to obtain a series of novel HNK derivatives, and investigated the anti-NPC effects of these compounds *in vitro* and *in vivo.*

## Results

### Chemistry

**Scheme 1.**
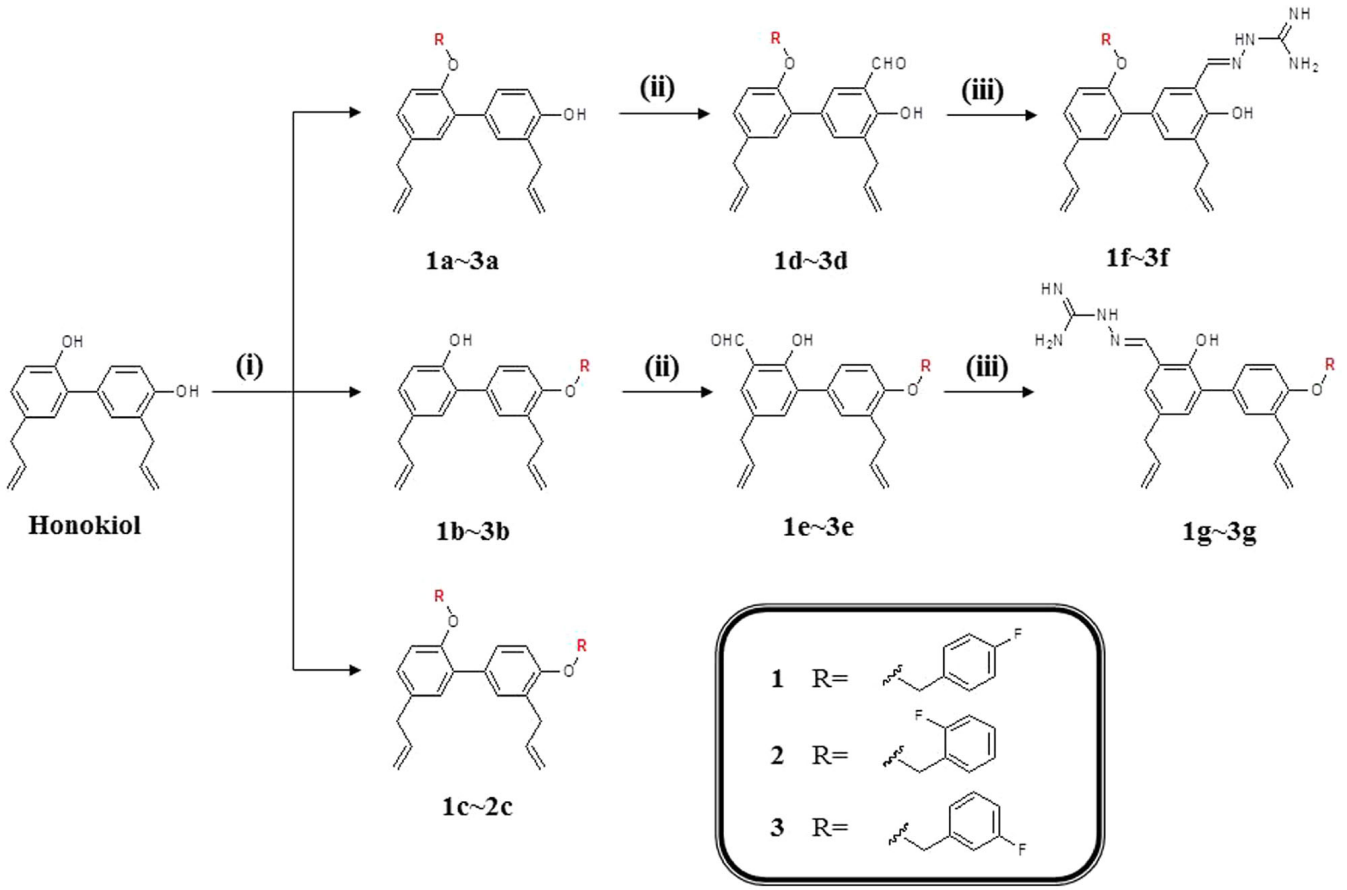
Design strategy and synthetic route for honokiol derivatives. (i) Fluorobenzylchloride, DMF, Na_2_CO_3_, 70–75 °C, 3–6 h. (ii) 25%NaOH, TBAB, CHCl_3_, 65 °C, 2–3 h. (ii) Acetic acid, aminoguanidine carbonate, 65 °C, 3–4 h.

As shown in Scheme 1, a series of HNK derivatives were designed and synthesised using honokiol as the starting compound. Compounds **1a**–**1c**, **2a**–**2c**, and **3a**–**3c** were synthesised from HNK through Williamson alkylation of alkyl halides. Next, compounds **1d**–**3d** and **1e**–**3e** were synthesised via C-formylation using NaOH/tetrabutylammonium bromide (TBAB) in CHCl_3_ as the catalyst. The latter compounds **1d**–**3d** and **1e**–**3e** were further reacted with aminoguanidine bicarbonate in the presence of catalytic amounts of hydrochloric acid to provide **1f**–**3f** and **1g**–**3g**, respectively. The chemical structures of all compounds were characterised by ^1^H-NMR, ^13^C-NMR, and HR-ESI-MS. The detailed synthesis processes and an overview of the physical and analytical data were described in the experimental section (see Supplementary Materials).

### Biological evaluation

#### Cytotoxic activity of HNK derivatives

All synthesised HNK derivatives were evaluated for their cytotoxic activities against the four cancer cell lines using the methyl thiazolyl tetrazolium (MTT) assay (Table 1). We observed that the O-alkylation of the substituted F atoms on the benzyl group of 2-OH or 4′-OH slightly improved the cytotoxic effect against cancer cells, whereas both 2-OH and 4′-OH alkylated derivatives were inactive at 50 μM. We further demonstrated that the HNK derivatives conjugated with aminoguanidine units exhibited better cytotoxicity than the parent compound. Among these, compounds **1e**, **1f**, **1g**, **2f**, **2g**, **3e**, **3f**, and **3g** showed excellent cytotoxicity against CNE-2Z, SG7901, MCF-7 and I-10 cells, with IC_50_ values of 5.30 to 20.14 μM. Compared to HNK, **1g** displayed 5.18-, 4.02-, 6.12-, and 5.87-fold enhancement of the cytotoxic effects on CNE-2Z, SG7901, MCF-7, and I-10 cells, respectively. Taken together, **1g** showed relatively low cytotoxic effect against normal mouse testicular leydig cells (TM3 cells), with IC_50_ values of 42.29 μM, indicating its selective inhibition (SI) to cancer cells (Table 2). The results indicated that **1g** had the most potent *in vitro* cytotoxic activity against the four cancer cell lines with low toxicity.

**Table 1.** Anti-proliferative activities of compounds (72 h, IC_50_, μM).

Compounds	CNE-2Z	SGC7901	MCF-7	I-10
HNK	31.27 ± 0.40	28.83 ± 0.36	41.80 ± 1.16	31.11 ± 0.99
DDP	1.04 ± 0.20	7.53 ± 0.14	16.55 ± 1.40	4.56 ± 0.81
**1a**	26.57 ± 1.09	20.33 ± 0.43	24.71 ± 0.54	25.10 ± 0.84
**1b**	23.92 ± 1.58	18.19 ± 1.30	19.76 ± 0.34	21.19 ± 1.30
**1c**	> 50	> 50	> 50	> 50
**2a**	24.18 ± 0.71	15.33 ± 1.06	19.64 ± 1.81	21.44 ± 0.40
**2b**	21.62 ± 1.12	21.47 ± 0.45	19.10 ± 1.05	20.66 ± 0.76
**2c**	> 50	> 50	> 50	> 50
**3a**	25.47 ± 0.18	16.47 ± 1.60	19.92 ± 0.37	25.10 ± 1.37
**3b**	21.53 ± 1.71	16.06 ± 0.37	18.50 ± 0.42	19.82 ± 0.33
**3c**	> 50	> 50	> 50	> 50
**1d**	20.69 ± 1.11	18.64 ± 1.20	20.82 ± 0.67	22.30 ± 0.55
**1e**	20.14 ± 0.34	17.77 ± 0.43	17.66 ± 0.54	18.48 ± 0.31
**1f**	15.33 ± 1.75	15.51 ± 1.08	17.51 ± 0.15	18.95 ± 0.26
**1g**	6.04 ± 0.45	7.17 ± 1.04	6.83 ± 1.94	5.30 ± 0.92
**2d**	23.25 ± 0.57	18.74 ± 1.06	16.01 ± 0.91	17.44 ± 1.34
**2e**	19.86 ± 1.04	23.13 ± 0.58	16.30 ± 1.18	20.82 ± 0.80
**2f**	19.18 ± 1.21	15.42 ± 0.31	12.41 ± 0.41	12.04 ± 2.51
**2g**	13.03 ± 0.60	14.40 ± 0.24	14.56 ± 0.93	14.11 ± 1.22
**3d**	23.73 ± 0.13	21.13 ± 0.50	17.92 ± 1.09	24.71 ± 0.46
**3e**	17.42 ± 0.40	14.10 ± 0.30	14.96 ± 0.93	14.80 ± 0.15
**3f**	15.88 ± 0.21	17.31 ± 2.16	13.30 ± 0.45	16.69 ± 0.76
**3g**	12.42 ± 0.54	10.66 ± 0.73	11.64 ± 1.06	12.88 ± 0.23

**Table 2.** Cytotoxic activity of **1g** on TM cells for 72 h (IC_50_, μM).

Compounds	TM3
**1g**	42.29 ± 3.69
HNK	58.68 ± 5.12
Geldanamycin	0.69 ± 0.08

#### Anti-proliferative activity of 1g in CNE-2Z cells

**Figure 1.**
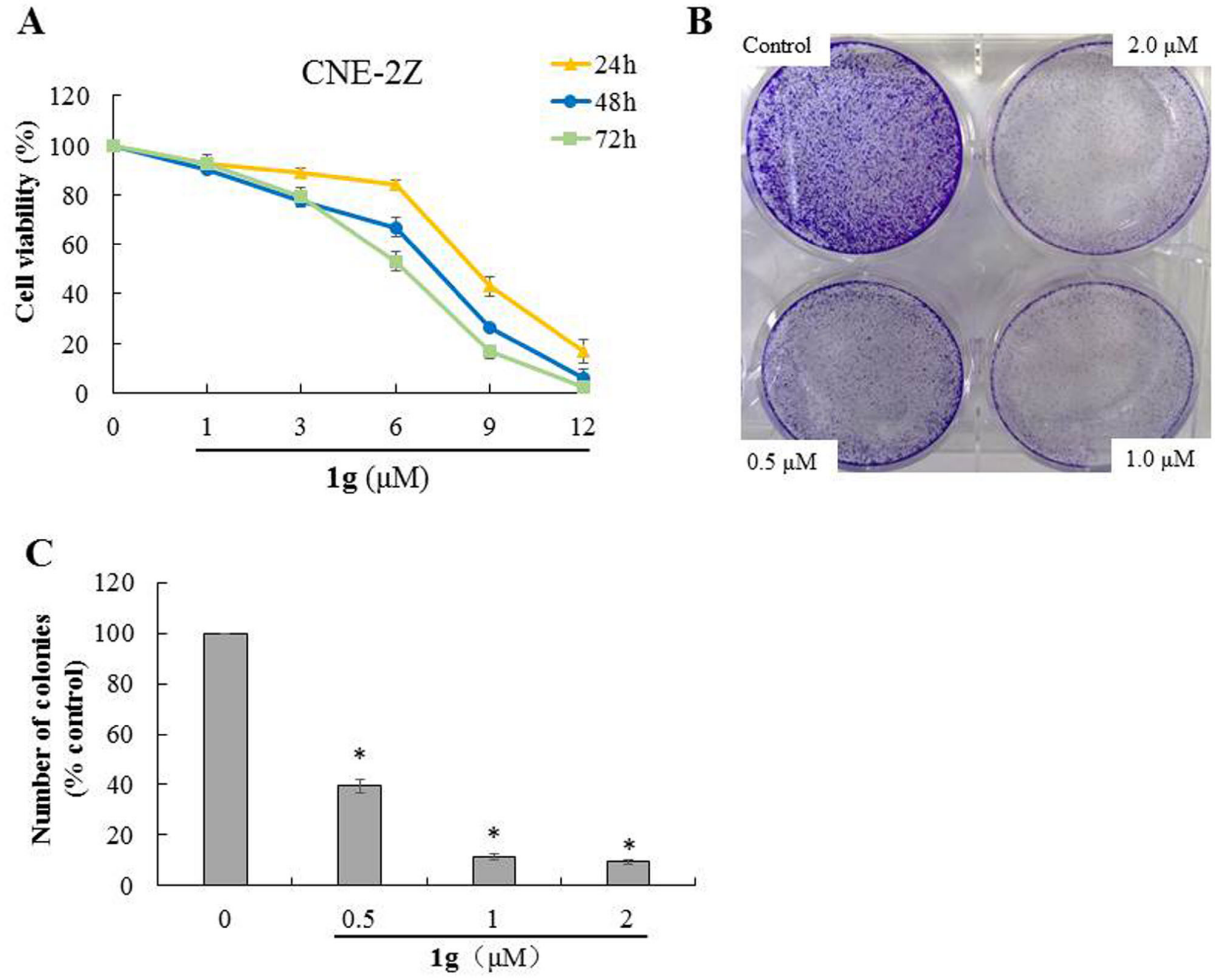
Inhibitory effect of **1g** on cell viability in CNE-2Z cells. (a) Cytotoxic activity of **1g** was measured using MTT assay. (b) Effects of **1g** on the ability of cells to form colonies in CNE-2Z cells using colony-formation assay. (c) Quantification of the colony-formation assay. *P < 0.05 compared with the control.

To understand the *in vitro* anti-cancer effect of **1g** in NPC, we analysed the anti-proliferative activity of **1g** on CNE-2Z cells after treated with different concentrations (0, 1, 3, 6, 9, and 12 μM) for three time points (24, 48, and 72 h). As shown in Figure 1a, **1g** significantly reduced the viability of CNE-2Z cells in a concentration- and time-dependent manner. Similarly, the colony-formation assay also revealed that **1g** inhibited cell growth at low concentrations (0, 0.5, 1, and 2 μM) (*P* < 0.05) (Figure 1b, c). These results indicated that compound **1g** exhibited potent anti-proliferative activity in CNE-2Z cells.

#### 1g suppressed migration and invasion of CNE-2Z cells

**Figure 2.**
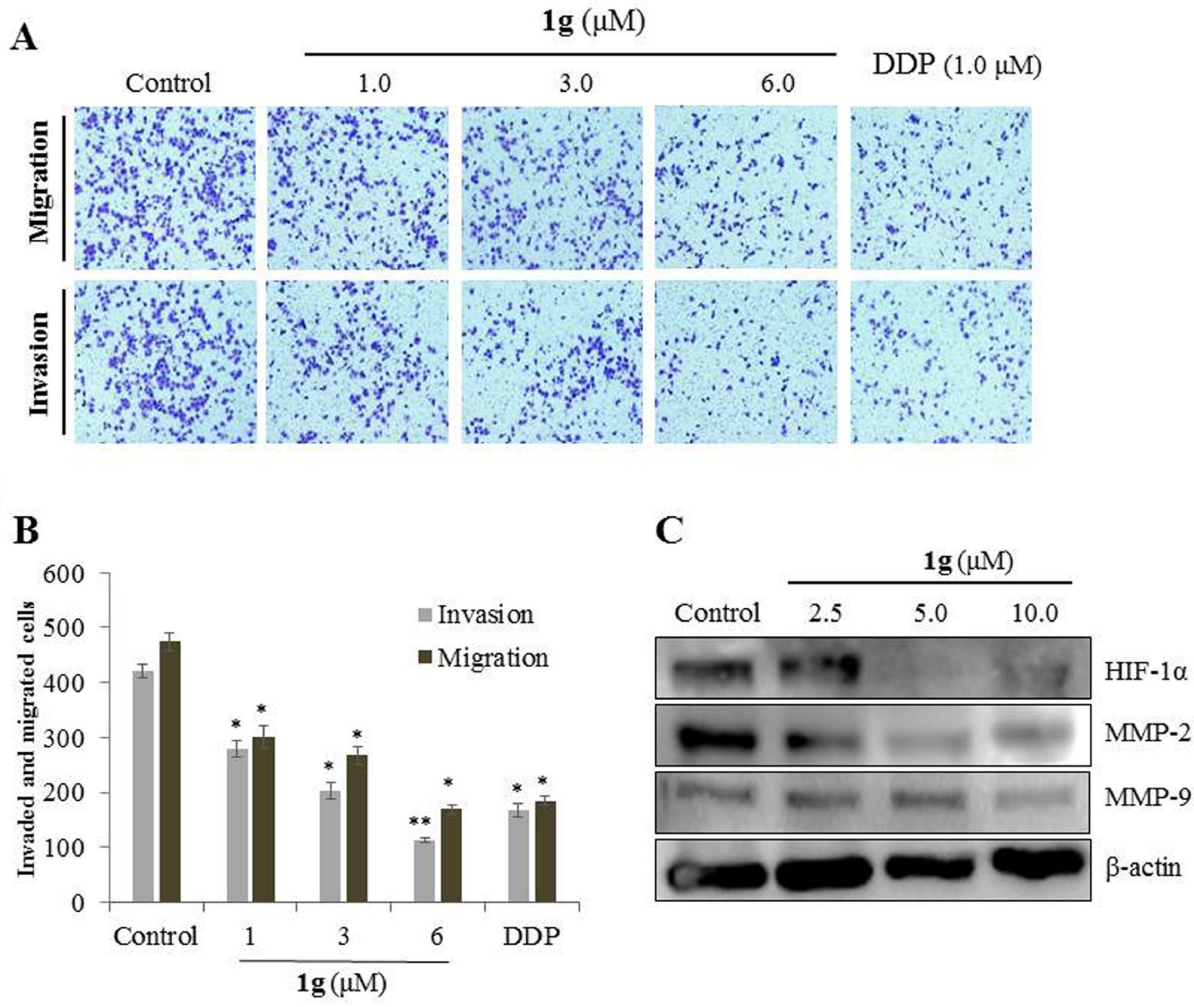
Effects of compound **1g** on the migration and invasion of CNE-2Z cells by a transwell assays. (a) CNE-2Z cells were seeded into a transwell chamber and exposed to **1g** (1.0, 3.0, and 6.0 μM) for 24 h to evaluate the migration and invasion activities. (b) Quantification analysis presented as the mean ± standard deviation. (c) Western blotting analyses of HIF-1α, MMP-2, and MMP-9 protein levels in CNE-2Z cells treated with various concentrations (2.5, 5.0, and 10.0 μM) of **1g**. β-Actin was used as an internal control. *P < 0.05, **P < 0.01compared with the control.

Transwell assays were performed to investigate the effects of **1g** on the migration and invasion abilities of CNE-2Z cells (Figure 2(a)). As shown in Figure 2b, **1g** treatments significantly reduced the number of cancer cells that crossed the membrane of the transwell chamber compared to the control (*P* < 0.05, *P* < 0.01). These results suggested that **1g** inhibited the migration and invasion of CNE-2Z cells in a concentration-dependent manner. Western blotting data showed that treated with **1g** significantly down-regulated the expression of HIF-1α, MMP-2, and MMP-9 (Figure 2(c)).

#### Effect of HIF-1α knockdown on migration and invasion of CNE-2Z cells by siRNA

**Figure 3.**
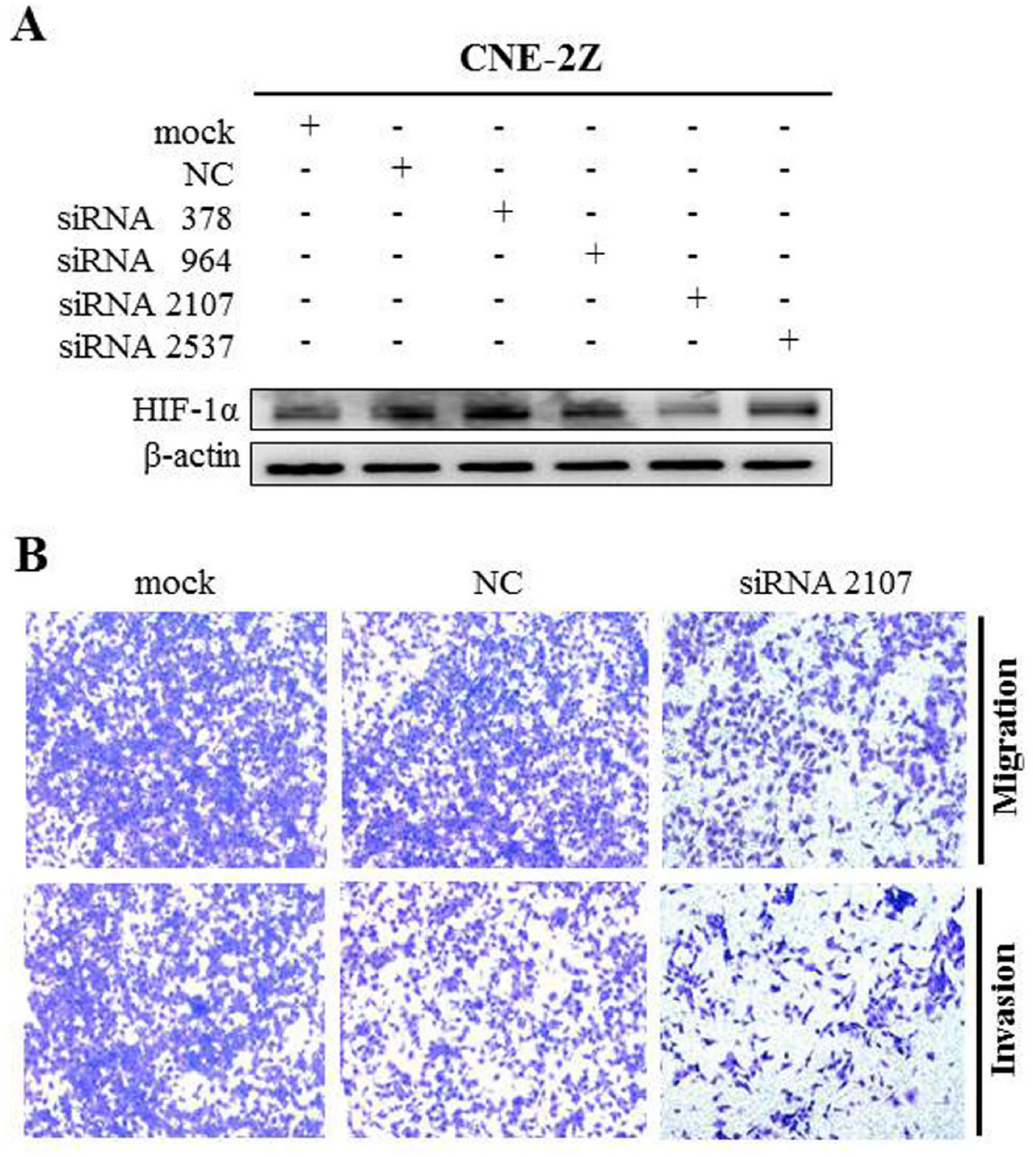
Knock-down of HIF-1α with siRNA protected against **1g** inhibited migration and invasion in CNE-2Z cells. (a) The cells were transfected with HIF-1α siRNA, and whole cell lysates were subjected to western blotting analysis. (b) Transwell assay was used to detect cell migration and invasion after 24 h transfection with HIF-1α siRNA (original magnification 200×).

To characterise the mechanisms, we investigated the effect after HIF-1α knockdown in CNE-2Z cells with invasive and metastatic potential. As shown in Figure 3, siRNA2107 can gradually down-regulated expression of HIF-1α, while the migration and invasion of CNE-2Z cells were significantly decreased after knockdown of HIF-1α. These results suggested that **1g** suppressed migration and invasion of CNE-2Z cells, which may be mimicked by down-regulation of HIF-1α.

#### 1g induced apoptosis in CNE-2Z cells

**Figure 4.**
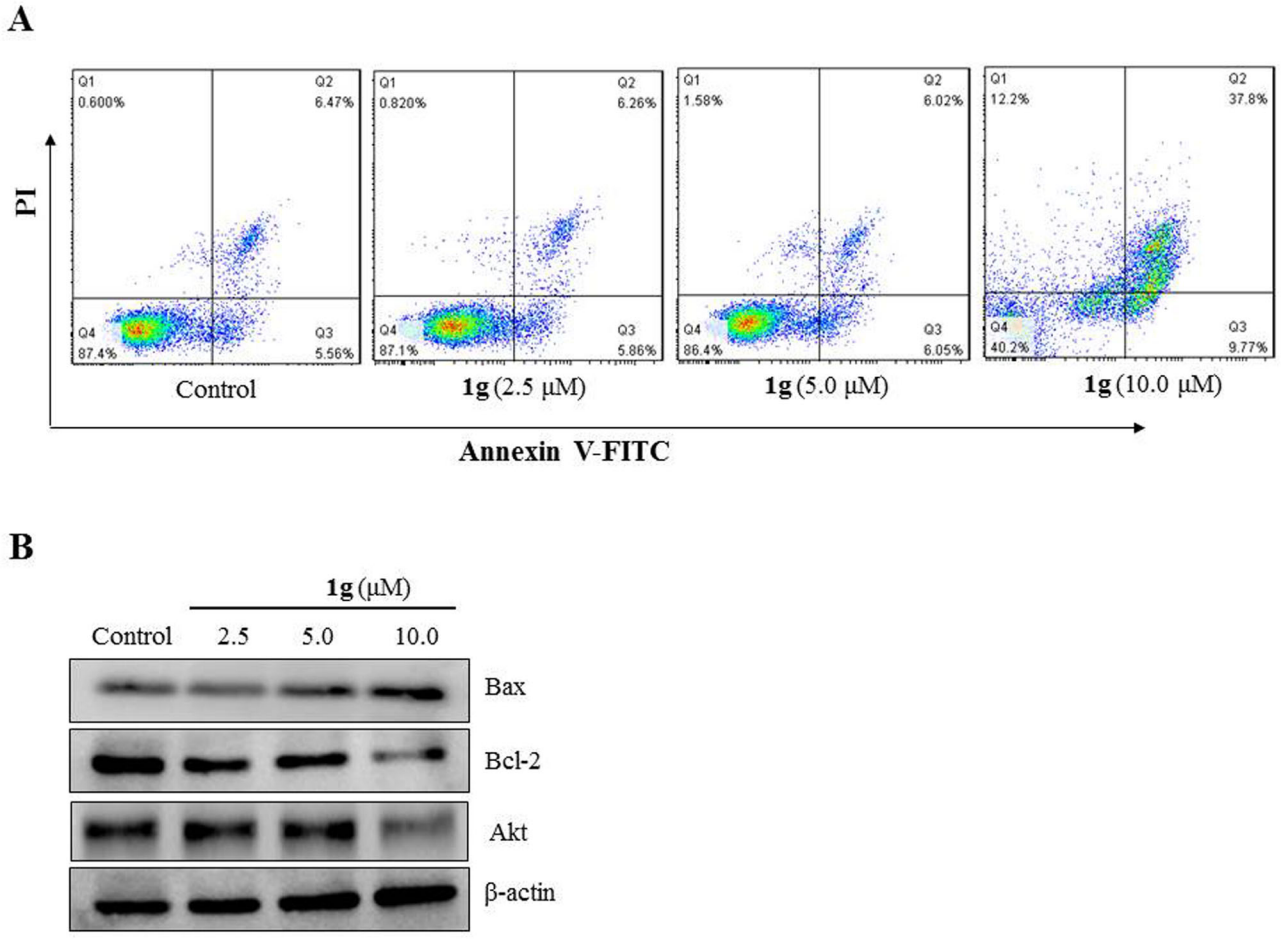
Compound **1g** induced cell death in human nasopharyngeal carcinoma CNE-2Z cells. (a) Flow cytometric analysis of cell death after treatment with various concentrations (2.5, 5.0, and 10.0 μM) of **1g** using annexin V-FITC/PI dual staining. (b) Western blotting analysis of Bax, Bcl-2, and Akt protein levels.

To clarify whether **1g** had the ability to induce apoptosis in CNE-2Z cells, we stained the cells with Annexin V-FITC and PI and performed flow cytometry. After treatment with increasing concentration of **1g** (2.5, 5, and 10 μM), the percentages of apoptotic cells increase to 12.12%, 12.07%, and 47.57%, respectively (Figure 4(a)). In addition, with an increase in **1g** concentration, the expression of the pro-apoptotic protein Bax gradually increased, while that of the anti-apoptotic proteins Bcl-2 and Akt decreased (Figure 4(b)). These results indicated that **1g** induced apoptosis in CNE-2Z cells.

#### In vivo anti-tumour efficacy of 1g

**Figure 5.**
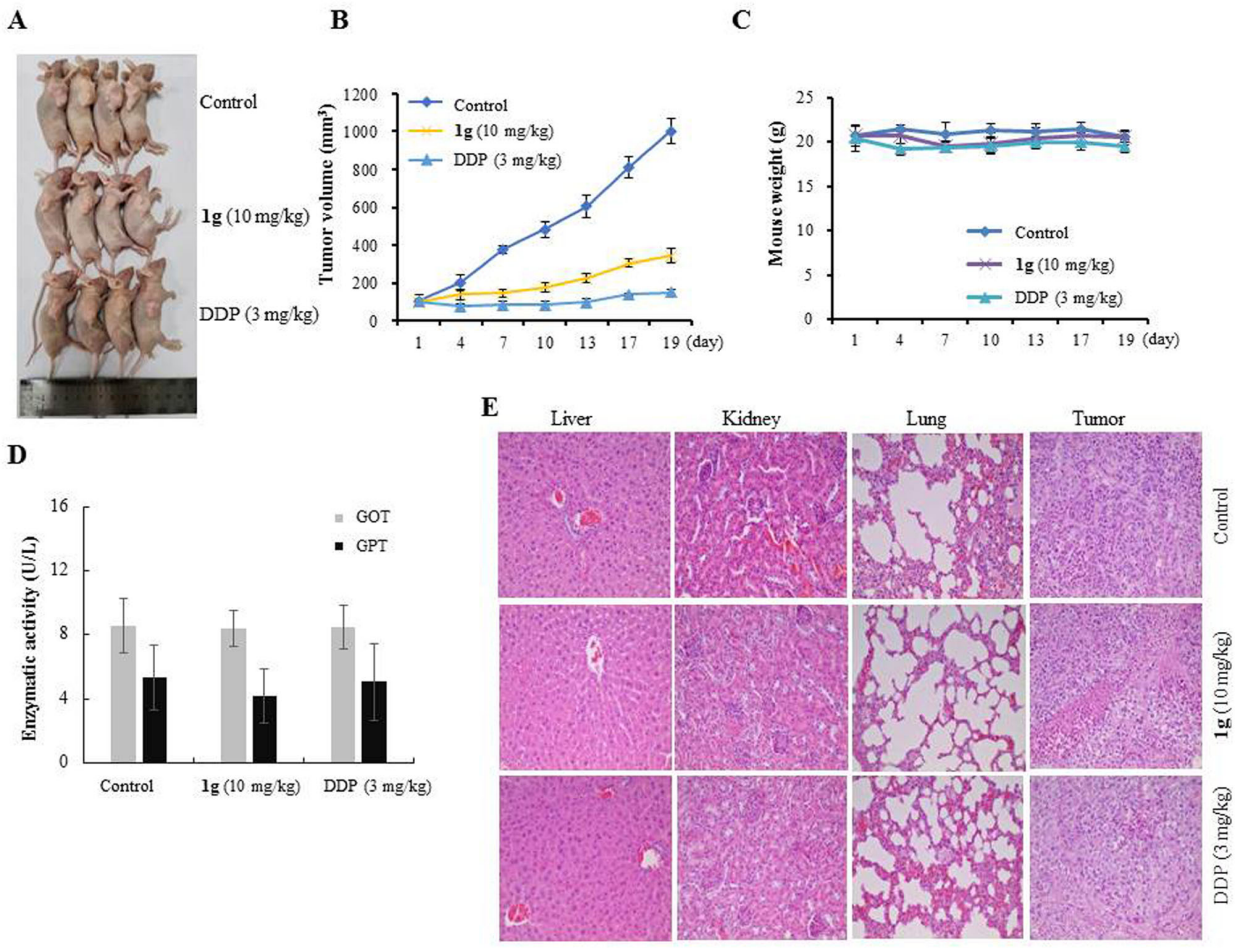
Anti-tumour efficacy of **1g** in CNE-2Z cell xenograft in nude mice (*n* = 4). (a) Representative tumours from each treatment group. (b) Tumour volume of each treatment group. (c) Body weight changes of nude mice. (d) The GPT and GOT of blood serum samples were determined by assay kit and the GPT and GOT activities are expressed as U/L. (e) H&E stained sections of the liver, kidney, lung, and tumour from the mice after treated with saline, DDP (3 mg/kg) and **1g** (10 mg/kg).

The *in vivo* anti-tumour efficacy of **1g** was evaluated in nude mice bearing CNE-2Z xenograft tumours, and the mice were treated with **1g** (10 mg/kg/3 days) for 19 days by intraperitoneal injection. DDP was used as a positive control. We observed that **1g** significantly prevented tumour growth compared with the control (Figure 5(a)). As shown in Figure 5b, **1g** showed potent inhibition of tumour growth and tumour volume to 343.59 mm^3^ compared with 1002.06** **mm^3^ in the control group, accounting for a 65.71% decrease in tumour volume (*P* < 0.01). Compound **1g** was well tolerated as it did not affect body weight loss (Figure 5(c)). As shown in Figure 5d, the enzymatic activity assay revealed that **1g** had nearly no effect on the GPT and GOT levels in serum. In addition, H&E staining of the liver, kidneys, and lungs revealed no serious damage after treatment with **1g** (Figure 5(e)). These results indicated that **1g** may be a potent anti-NPC agent with low totoxicity.

## Discussion

Several studies have reported that the four main treatments for NPC, including radiation, chemotherapy, molecule targeted therapy, and immune checkpoint therapy.^3^^,^^36^ However, the main challenges in NPC treatment include distant metastasis, chemo-resistance, and local recurrence.^5^ Recently, HIF-1α has been recognised as a promising biomarker for NPC treatment.^13^ Thus, small molecules HIF-1α inhibitors could show significant anti-tumour effects in NPC.

In our previous studies, we had found that HNK and its derivative exhibited potential inhibitory effect of HIF-1α and anti-tumour properties.^30^^,^^37^ Current literature on the anti-tumour properties and mechanisms of action of HNK derivatives has uncovered their potential in prevention and chemotherapy. In an attempt to improve its therapeutic profile, there has been an interest in obtaining novel HNK derivatives with enhanced anti-cancer activity, increased cell selectivity, and identification of the precise mechanisms of action in cell death.^28^^,^^29^ Based on the previous studies, we aimed to enhance the cytotoxic activity of the parent compound focusing on developing HNK derivatives containing aminoguanidine units and preliminarily assessing their mechanisms of action in CNE-2Z cells.

Highly electronegative and small fluorine atoms can play a significant role in medicinal chemistry and structural modification of natural products.^38^ Fluorinated drug candidates can improve several physicochemical and pharmacokinetic properties, such as membrane permeation, metabolic stability, and binding affinity to target proteins.^39^ In this study, we designed and synthesised a series of novel HNK derivatives based on fluorobenzyl-modified HNK conjugated with an aminoguanidine unit. As shown Table 1, the O-fluorobenzylation of 2-OH or 4′-OH might slightly improve cytotoxic effect against four cancer cell lines. Consistent with previous report, it was observed that O-fluorobenzylation of 4′-hydroxy group of HNK more than O-fluorobenzylation of 2-hydroxy group slightly improved the cytotoxic activity against four cancer cells.^40^ Our results suggest that the 2-OH and 4′-OH of HNK were part of the pharmacophoric moiety in the inhibiting of tumour growth. However, the position of the fluorine atom had little effect on cytotoxicity in cancer cells. Among these, compound **1g**, a novel HNK derivative conjugated with an aminoguanidine moiety, exhibited the highest cytotoxic activity against CNE-2Z, SGC7901, MCF-7, and I-10 cells with IC_50_ values of 6.04, 7.17, 6.83, and 5.30 μM, respectively.

The increased cytotoxic activity and cell sensitivity of compound **1g** illustrated that HNK associated with the aminoguanidine moiety is an effective strategy for structural modification. During structural modifications, reducing the toxicity and side effects of compounds is as important as increasing their biological activity and sensitivity.^41^ Relative to the strong cytotoxicity of compound **1g** against CNE-2Z cells, it showed low toxicity in normal TM-3 cells, with an SI value of 7.0. Together, **1g** also induced apoptosis in CNE-2Z cells by up-regulating of Bax and down-regulating of Bcl-2 and Akt protein expression. In addition, the *in vivo* experiment showed that **1g** caused less damage to the liver, kidney, and lung tissues at therapeutic doses.

The underlying detailed mechanisms of the anti-cancer properties of **1g** were explored. The invasion and metastasis of cell are the main reasons for the failure of NPC treatment.^5^^,^^42^ We observed that **1g** significantly inhibited migration and invasion in CNE-2Z cells involving decreasing MMP-2, MMP-9, as well as HIF-1α protein levels. The key hypoxic regulator HIF-1α, has been shown to be highly expressed in NPC and was a promising involve in cancer cell survival, proliferation, apoptosis, invasion, metastasis etc.^13^^,^^43^^,^^44^ To date, although many small molecule HIF-1α inhibitors have emerged, there are no clinically available HIF-1α inhibitors.^45–47^ Transfection of CNE-2Z cells with HIF-1α siRNA resulted in reduced cell invasion and migration. In the present study, we synthesised a novel HIF-1α inhibitor from a natural source, compound **1g**, which exhibited potent anti-NPC properties *in vitro* and *in vivo*.

## Conclusions

Twenty-one novel HNK derivatives were synthesised and investigated for their cytotoxic activity. Among these HNK derivavites, **1g** showed significant broad-spectrum and potent cytotoxic activity. Transwell assay revealed that **1g** could prevent migration and invasion of CNE-2Z cells by down-regulating of HIF-1ɑ, MMP-2 and MMP-9. In addition, **1g** also significantly induced apoptosis of CNE-2Z cells. Furthermore, a CNE-2Z xenograft model experiment demonstrated that **1g** significantly inhibited tumour growth with low toxicity. Collectively, our findings suggest that **1g** is a potential anti-NPC agent that warrants further investigation.

## Experimental section

### Reagents and instruments

HNK (>98% by HPLC) was purchased from Xinmingtai Chemical Co., Ltd (Hubei, China). All reagents and solvents were of analytical grade and were purchased from commercial sources. All NMR spectra of the compounds were measured using Bruker equipment (Fallanden, Switzerland). Mass spectra of the compounds were analysed using an Agilent 1290/6538 accurate Q-TOF mass spectrometer (Agilent Technologies, USA). The purity of all derivatives was determined by HPLC (Shimadzu, Tokyo, Japan) and found to be in the 96–99% range.

### Synthesis of HNK derivatives 1a–3a, 1b–3c, and 1c–3c

HNK (2.1 mmol) was dissolved in dimethyl formamide (DMF solution, 5 ml), and then sodium carbonate solution (25%) was added at room temperature and stirred for another 0.5 h. The 4-fluorobenzyl-chloride (2.1 mmol) or 3-fluorobenzylchloride (2.1 mmol) or 5-fluorobenzylchloride (2.1 mmol) were slowly added to react and kept stirred for 3–6 h at 70–75 °C, and then detected by TLC analysis, respectively. The reaction was quenched with water, the pH was adjusted to weakly acidic with 1 M HCl and the aqueous layers were extracted twice with ethyl acetate. At the same time, the ethyl acetate layer was washed three times with saturated NaCl solution to wash away the residual DMF solvent. The combined extract was dried over Na_2_SO_4_ and concentrated under reduced pressure. Finally, the residue was purified by silica gel chromatography using petroleum ether: ethyl acetate = 100: 1 to 20: 1 to afford **1a**–**3a**, **1b**–**3c**, and **1c**–**3c**, respectively.

#### 2-((4-Fluorobenzyl)oxy)-honokiol (1a)

Yellow solid, yield: 18%; ^1^H-NMR (400 MHz, CDCl_3_): *δ* 7.45–7.38 (m, 2H), 7.28 − 7.25 (m, 2H), 7.11 − 7.01 (m, 4H), 6.97 (d, *J* = 9.0 Hz, 1H), 6.89 (d, *J* = 7.9 Hz, 1H), 6.06–5.91 (m, 2H), 5.20–5.09 (m, 2H), 5.07 (s, 2H), 5.06–5.02 (m, 2H), 3.47 (d, *J* = 6.2 Hz, 2H), 3.34 (d, *J* = 6.7 Hz, 2H). ^13^C-NMR (100 MHz, CDCl_3_): *δ* 163.70, 155.98, 150.85, 137.83, 136.46, 132.87, 132.23, 130.76, 130.26, 130.10, 129.61, 129.05, 128.97, 128.85, 127.96, 127.78, 116.06, 115.65, 115.65, 115.61, 115.43, 112.26, 69.47, 39.46, 34.58. HR-ESI-MS: molecular formula C_25_H_23_FO_2_ as revealed at *m/z* [M + Na]^+^ 397.15744.

#### 4'-((4-fluorobenzyl)oxy)-honokiol (2a)

Yellow solid, yield: 15%; ^1^H-NMR (400 MHz, CDCl_3_): *δ* 7.54 (t, *J* = 7.1 Hz, 1H), 7.36–7.25 (m, 3H), 7.18 (t, *J* = 7.5 Hz, 1H), 7.14–7.07 (m, 1H), 7.07–7.01 (m, 3H), 6.89 (d, *J* = 8.0 Hz, 1H), 6.07–5.92 (m, 2H), 5.19 (s, 2H), 5.13–5.01 (m, 4H), 3.48 (d, *J* = 6.7 Hz, 2H), 3.34 (d, *J* = 6.7 Hz, 2H). ^13^C-NMR (100 MHz, CDCl_3_): *δ* 161.59, 155.90, 150.85, 137.81, 136.45, 132.19, 130.70, 130.23, 130.19, 129.66, 129.58, 129.38, 128.83, 127.98, 127.77, 124.28, 116.02, 115.61, 115.57, 115.45, 115.24, 112.27, 63.92, 39.44, 34.55. HR-ESI-MS: molecular formula C_25_H_23_FO_2_ as revealed at *m/z* [M + H]^+^ 375.17551.

#### 4',2-Di-((4-fluorobenzyl)oxy)-honokiol (3a)

Yellow solid, yield: 13%; ^1^H-NMR (400 MHz, CDCl_3_): *δ* 7.38–7.32 (m, 1H), 7.28–7.15 (m, 4H), 7.06–6.98 (m, 3H), 6.95 (d, *J* = 8.1 Hz, 1H), 6.89 (d, *J* = 8.0 Hz, 1H), 6.08–5.91 (m, 2H), 5.14–5.03 (m, 4H), 5.11 (s, 2H), 3.50 (d, *J* = 6.8 Hz, 2H), 3.34 (d, *J* = 6.8 Hz, 2H). ^13^C-NMR (100 MHz, CDCl_3_): *δ* 164.27, 155.84, 150.85, 139.78, 137.82, 136.42, 132.24, 130.82, 130.26, 130.21, 130.13, 129.72, 128.86, 127.97, 127.75, 122.42, 116.11, 115.65, 115.59, 114.90, 114.08, 112.22, 69.29, 39.45, 34.57. HR-ESI-MS: molecular formula C_25_H_23_FO_2_ as revealed at *m/z* [M + Na]^+^ 397.15738.

#### 2-((2-Fluorobenzyl)oxy)-honokiol (1b)

Yellow solid, yield: 21%; ^1^H-NMR (400 MHz, CDCl_3_): *δ* 7.36–7.26 (m, 4H), 7.15 (d, *J* = 2.2 Hz, 1H), 7.07 (dd, *J* = 8.3, 2.3 Hz, 1H), 7.03–6.96 (m, 2H), 6.93 (d, *J* = 8.3 Hz, 1H), 6.84 (d, *J* = 8.1 Hz, 1H), 6.09–5.91 (m, 2H), 5.22–5.03 (m, 4H), 4.97 (s, 2H), 3.42 (d, *J* = 6.1 Hz, 2H), 3.37 (d, *J* = 6.8 Hz, 2H). ^13^C-NMR (100 MHz, CDCl_3_): *δ* 163.48, 153.83, 153.25, 137.68, 136.37, 133.10, 133.08, 133.05, 131.74, 131.16, 131.04, 129.02, 128.83, 128.75, 128.00, 124.59, 116.56, 115.67, 115.45, 115.35, 115.14, 113.69, 70.17, 39.47, 35.27. HR-ESI-MS: molecular formula C_25_H_23_FO_2_ as revealed at *m/z* [M + Na]^+^ 397.15747.

#### 4'-((2-Fluorobenzyl)oxy)-honokiol (2b)

Yellow solid, yield: 32%; ^1^H-NMR (600 MHz, CD_3_OD): *δ* 7.55 (td, *J* = 7.5, 1.8 Hz, 1H), 7.36–7.34 (m, 1H), 7.32 (d, *J* = 2.3 Hz, 1H), 7.19 (td, *J* = 7.5, 1.1 Hz, 1H), 7.15–7.11 (m, 1H), 7.03 (d, *J* = 8.4 Hz, 1H), 7.01 (d, *J* = 2.3 Hz, 1H), 6.92 (dd, *J* = 8.2, 2.3 Hz, 1H), 6.79 (d, *J* = 8.2 Hz, 1H), 6.03 − 5.92 (m, 2H), 5.17 (s, 2H), 5.08 − 4.95 (m, 4H), 3.42 (d, *J* = 1.6 Hz, 2H), 3.30 (d, *J* = 1.5 Hz, 2H). ^13^C-NMR (150 MHz, CD_3_OD): *δ* 161.44, 155.08, 152.03, 138.14, 136.97, 131.71, 131.14, 130.67, 130.20, 129.62, 129.52, 128.24, 127.93, 127.62, 124.56, 123.97, 115.54, 114.85, 114.71, 114.18, 114.01, 111.28, 63.72, 39.03, 34.18. HR-ESI-MS: molecular formula C_25_H_23_FO_2_ as revealed at *m/z* [M + H]^+^ 375.17554

#### 4',2-Di-((4-fluorobenzyl)oxy)-honokiol (3b)

Yellow solid, yield: 27%; ^1^H-NMR (600 MHz, CD_3_OD): *δ* 7.38 (td, *J* = 8.0, 5.8 Hz, 1H), 7.35–7.33 (m, 2H), 7.27 (d, *J* = 7.6 Hz, 1H), 7.21 (ddd, *J* = 9.9, 2.6, 1.5 Hz, 1H), 7.05–7.01 (m, 1H), 7.01 (d, *J* = 2.4 Hz, 1H), 6.99–6.96 (m, 1H), 6.94–6.90 (m, 1H), 6.79 (d, *J* = 8.2 Hz, 1H), 6.06–5.91 (m, 2H), 5.13 (s, 2H), 5.11–4.95 (m, 4H), 3.46 (dt, *J* = 6.5, 1.6 Hz, 2H), 3.29 (dt, *J* = 6.5, 1.5 Hz, 2H). ^13^C- NMR (150 MHz, CD_3_OD): *δ* 163.80, 155.02, 152.03, 138.13, 136.99, 131.63, 131.14, 130.73, 130.20, 129.88, 129.82, 128.16, 128.04, 127.93, 127.62, 122.42, 115.54, 114.22, 114.01, 113.84, 113.48, 111.23, 68.81, 39.02, 34.23. HR-ESI-MS: molecular formula C_25_H_23_FO_2_ as revealed at *m/z* [M + Na]^+^ 397.15741.

##### 2-((5-Fluorobenzyl)oxy)-honokiol (1c)

Yellow solid, yield: 24%; ^1^H-NMR (600 MHz, CD_3_OD): *δ* 7.49–7.45 (m, 2H), 7.44–7.40 (m, 1H), 7.32–7.26 (m, 4H), 7.12–7.06 (m, 4H), 7.03–6.97 (m, 3H), 6.00–5.90 (m, 2H), 5.08 (s, 2H), 5.08–4.95 (m, 4H), 4.95 (s, 2H), 3.39 (dt, *J* = 6.6, 1.5 Hz, 2H), 3.35 (dd, *J* = 6.7, 1.5 Hz, 2H). ^13^C-NMR (150 MHz, CD_3_OD): *δ* 161.72, 160.09, 153.78, 152.35, 136.33, 135.39, 132.11, 131.47, 129.70, 129.52, 128.91, 128.84, 127.53, 127.51, 127.47, 127.45, 126.53, 126.25, 113.56, 113.41, 113.31, 113.16, 113.13, 112.99, 112.80, 112.75, 112.19, 109.73, 68.36, 67.51, 37.50, 32.64. HR-ESI-MS: molecular formula C_32_H_28_F_2_O_2_ as revealed at *m/z* [M + Na]^+^ 505.19501.

#### 4'-((5-Fluorobenzyl)oxy)-honokiol (2c)

Yellow solid, yield: 25%; ^1^H-NMR (600 MHz, CD_3_OD): *δ* 7.55 (td, *J* = 7.6, 1.8 Hz, 1H), 7.38–7.28 (m, 5H), 7.19 (td, *J* = 7.5, 1.1 Hz, 1H), 7.14 (ddd, *J* = 10.3, 8.3, 1.1 Hz, 1H), 7.11–7.08 (m, 3H), 7.08–7.04 (m, 2H), 7.02 (d, *J* = 8.4 Hz, 1H), 6.02–5.88 (m, 2H), 5.18 (s, 2H), 5.10–4.91 (m, 6H), 3.38–3.35 (m, 4H). ^13^C-NMR (150 MHz, CD_3_OD): *δ* 161.46, 159.64, 155.22, 153.78, 137.81, 136.82, 133.10, 131.33, 131.11, 131.03, 130.44, 129.63, 129.54, 129.48, 129.33, 127.99, 127.79, 124.42, 123.96, 123.85, 114.86, 114.71, 114.68, 114.54, 114.32, 114.23, 113.53, 111.25, 64.34, 63.72, 39.00, 34.09. HR-ESI-MS: molecular formula C_32_H_28_F_2_O_2_ as revealed at *m/z* [M + Na]^+^ 505.19516.

#### 4',2-Di-((5-fluorobenzyl)oxy)-honokiol (3c)

Yellow solid, yield: 20%; ^1^H-NMR (600 MHz, CD_3_OD): *δ* 7.39 (td, *J* = 7.9, 5.9 Hz, 1H), 7.34 (d, *J* = 2.3 Hz, 1H), 7.32–7.26 (m, 3H), 7.24–7.19 (m, 1H), 7.12–7.06 (m, 3H), 7.05–6.95 (m, 5H), 6.01–5.93 (m, 2H), 5.15 (s, 2H), 5.09–4.95 (m, 6H), 3.46– 3.41 (dt, *J* = 6.7, 1.5 Hz, 2H), 3.35 (dt, *J* = 6.7, 1.5 Hz, 2H). ^13^C-NMR (150 MHz, CD_3_OD): *δ* 163.81, 162.06, 155.22, 153.75, 137.81, 136.81, 133.05, 131.28, 131.09, 130.43, 129.89, 129.70, 128.04, 127.88, 127.79, 122.46, 122.40, 114.36, 114.31, 114.01, 113.87, 113.84, 113.69, 113.55, 113.51, 113.39, 113.36, 111.22, 69.67, 68.86, 38.99, 34.10. HR-ESI-MS: molecular formula C_32_H_28_F_2_O_2_ as revealed at *m/z* [M + Na]^+^ 505.19507.

### Synthesis of honokiol derivatives 1d–3d

Compounds **1a**, **2a**, and **3a** were used as intermediates for the synthesis of 5′-formylation derivatives. In brief, the compounds and 25% NaOH/TBAB in CHCl_3_ were stirred for 2–3 h at 65 °C, and then detected by TLC analysis. When the reaction was complete, the mixture was neutralised with hydrochloric acid and extracted twice with ethyl acetate. The combined extract was dried over Na_2_SO_4_ and concentrated under reduced pressure. Finally, the residue was purified by silica gel chromatography using an eluent (petroleum ether: ethyl acetate = 120: 1 to 20: 1) to obtain **1d**, **2d**, and **3d**, respectively.

#### 2-((4-Fluorobenzyl)oxy)-5'-formy-honokiol (1d)

Yellow solid, yield: 33.5%; ^1^H-NMR (400 MHz, CDCl_3_): *δ* 11.30 (s, 1H), 9.84 (s, 1H), 7.37–7.29 (m, 5H), 7.25 (d, *J* = 2.2 Hz, 1H), 7.01 (t, *J* = 8.6 Hz, 2H), 6.89 (d, *J* = 8.4 Hz, 1H), 6.05–5.79 (m, 2H), 5.10–4.95 (m, 6H), 3.41 (d, *J* = 6.6 Hz, 2H), 3.34 (d, *J* = 6.7 Hz, 2H). ^13^C-NMR (100 MHz, CDCl_3_): *δ* 196.81, 157.48, 157.33, 155.89, 138.18, 136.81, 136.75, 133.02, 132.09, 131.39, 130.90, 130.21, 128.99, 128.94, 128.91, 128.89, 128.23, 120.67, 117.84, 116.51, 115.72, 115.55, 115.34, 111.45, 69.43, 39.02, 34.56. HR-ESI-MS: molecular formula C_26_H_23_FO_3_ as revealed at *m/z* [M + H]^+^ 403.17087.

#### 2-((2-Fluorobenzyl)oxy)-5'-formy-honokiol (2d)

Yellow solid, yield: 21.3%; ^1^H-NMR (400 MHz, CDCl_3_): *δ* 11.37 (s, 1H), 9.91 (s, 1H), 7.55 (t, *J* = 7.6 Hz, 1H), 7.45–7.40 (m, 3H), 7.35–7.29 (m, 2H), 7.18 (t, *J* = 7.4 Hz, 1H), 7.12–7.08 (m, 1H), 7.01 (d, *J* = 8.4 Hz, 1H), 6.10–5.93 (m, 2H), 5.20 (s, 2H), 5.15–5.04 (m, 4H), 3.50 (d, *J* = 7.0 Hz, 2H), 3.42 (d, *J* = 6.6 Hz, 2H). ^13^C-NMR (100 MHz, CDCl_3_): *δ* 196.80, 161.54, 157.34, 155.80, 138.20, 136.82, 136.77, 132.08, 131.38, 130.87, 130.24, 129.52, 129.35, 129.01, 128.95, 128.26, 124.27, 120.67, 116.51, 115.71, 115.37, 115.16, 111.43, 63.88, 39.02, 34.57. HR-ESI-MS: molecular formula C_26_H_23_FO_3_ as revealed at *m/z* [M + H]^+^ 403.1718.

#### 2-((5-Fluorobenzyl)oxy)-5'-formy-honokiol (3d)

Yellow solid, yield: 17.6%; ^1^H-NMR (400 MHz, CDCl_3_): *δ* 11.30 (s, 1H), 9.84 (s, 1H), 7.37–7.30 (m, 3H), 7.30–7.24 (m, 2H), 7.17–7.09 (m, 2H), 6.94 (td, *J* = 8.5, 2.6 Hz, 1H), 6.87 (d, *J* = 8.3 Hz, 1H), 6.05–5.83 (m, 2H), 5.10–4.96 (m, 6H), 3.44 (d, *J* = 6.6 Hz, 2H), 3.34 (d, *J* = 6.7 Hz, 2H). ^13^C-NMR (100 MHz, CDCl_3_): *δ* 195.77, 160.85, 156.29, 154.71, 137.15, 135.77, 135.67, 131.07, 130.36, 129.92, 129.15, 129.09, 129.00, 128.00, 127.85, 127.21, 121.35, 119.63, 115.48, 114.75, 113.74, 113.53, 110.35, 68.20, 37.98, 33.53. HR-ESI-MS: molecular formula C_26_H_23_FO_3_ as revealed at *m/z* [M-H]^-^ 401.1558.

### Synthesis of honokiol derivatives 1e–3e

Compounds **1b**, **2b**, and **3b** were used as intermediates for the synthesis of 3-formylation derivatives. In brief, the compounds and 25% NaOH/TBAB in CHCl_3_ were stirred for 3 h at 65 °C, and then detected by TLC analysis. When the reaction was complete, the mixture was neutralised with hydrochloric acid and extracted twice with ethyl acetate for twice. The combined extract was dried over Na_2_SO_4_ and concentrated under reduced pressure. Finally, the residue was purified by silica gel chromatography using an eluent (petroleum ether: ethyl acetate = 120: 1 to 20: 1) to obtain **1e**, **2e**, and **3e**, respectively.

#### 4'-((4-Fluorobenzyl)oxy)-3-formy-honokiol (1e)

Yellow solid, yield: 27%; ^1^H-NMR (600 MHz, CD_3_OD): *δ* 9.94–9.90 (m, 1H), 7.48 (q, *J* = 6.2, 4.5 Hz, 2H), 7.46–7.43 (m, 1H), 7.42 (q, *J* = 2.3 Hz, 1H), 7.38 (dt, *J* = 8.3, 2.2 Hz, 1H), 7.36 (t, *J* = 1.7 Hz, 1H), 7.12–7.09 (m, 2H), 7.05–7.00 (m, 1H), 6.06– 5.94 (m, 2H), 5.15–4.97 (m, 6H), 3.47–3.39 (m, 4H). ^13^C-NMR (150 MHz, CD_3_OD): *δ* 197.43, 163.23, 156.71, 155.75, 137.56, 137.10, 136.80, 133.51, 132.02, 131.64, 130.60, 129.81, 129.04, 129.02, 128.96, 128.32, 128.02, 120.89, 115.07, 114.83, 114.69, 114.35, 111.35, 69.02, 38.47, 34.14. HR-ESI-MS: molecular formula C_26_H_23_FO_3_ as revealed at *m/z* [M + H]^+^ 403.1711.

#### 4'-((2-Fluorobenzyl)oxy)-3-formy-honokiol (2e)

Yellow solid, yield: 22.5%; ^1^H-NMR (400 MHz, CDCl_3_): *δ* 11.20 (s, 1H), 9.78 (s, 1H), 7.58–7.50 (m, 2H), 7.29–7.18 (m, 2H), 7.11–7.05 (m, 2H), 7.04–6.93 (m, 3H), 6.00–5.81 (m, 2H), 5.07–4.93 (m, 6H), 3.38–3.34 (m, 2H), 3.33–3.28 (m, 2H). ^13^C-NMR (100 MHz, CDCl_3_): *δ* 195.80, 160.57, 157.50, 152.72, 137.64, 136.48, 134.72, 132.20, 131.66, 129.61, 128.92, 128.67, 128.54, 128.37, 127.69, 127.11, 123.13, 119.00, 115.21, 114.79, 114.32, 114.11, 112.17, 63.64, 38.36, 32.12. HR-ESI-MS: molecular formula C_26_H_23_FO_3_ as revealed at *m/z* [M-H]^-^ 401.1543

#### 4'-((5-Fluorobenzyl)oxy)-3-formy-honokiol (3e)

Yellow solid, yield: 20.4%; ^1^H-NMR (400 MHz, CDCl_3_): *δ* 11.22 (s, 1H), 9.80 (s, 1H), 7.58 (d, *J* = 2.2 Hz, 1H), 7.52 (d, *J* = 2.2 Hz, 1H), 7.20 (td, *J* = 7.9, 5.8 Hz, 1H), 7.10 − 7.03 (m, 2H), 6.99 (d, *J* = 7.7 Hz, 1H), 6.96–6.85 (m, 3H), 5.96–5.83 (m, 2H), 5.07– 4.95 (m, 6H), 3.38 (d, *J* = 6.6 Hz, 2H), 3.31 (d, *J* = 6.7 Hz, 2H). ^13^C-NMR (100 MHz, CDCl_3_): *δ* 195.72, 163.08, 157.59, 152.66, 137.62, 136.44, 134.66, 132.26, 131.52, 129.71, 129.02, 128.94, 128.89, 128.44, 127.68, 127.22, 121.36, 119.01, 115.35, 114.82, 113.75, 113.04, 112.21, 68.90, 38.35, 32.04. HR-ESI-MS: 401.1526 [M + H]^+^, (calcd for C_26_H_23_FO_3_, 403.1631). HR-ESI-MS: molecular formula C_26_H_23_FO_3_ as revealed at *m/z* [M-H]^-^ 401.1562.

### Synthesis of honokiol derivatives 1f–3f and 1g–3g

Compounds **1d**–**3d** and **1e**–**3e** were used as intermediates for the synthesis of hydrozinecarboximidamide-HNK derivatives. In brief, the compounds **1d**–**3d** (0.05–0.28 mmol) and acetic acid/aminoguanidine carbonate (0.18–0.39 mmol) in ethanol were stirred for 3–4 h at 65 °C, and then detected by TLC analysis, respectively. The reaction was quenched with water and extracted twice with ethyl acetate. The combined extract was dried over Na_2_SO_4_ and concentrated under reduced pressure. Finally, the residue was purified by a 2535Q semi-prep HPLC system (Waters, USA) using a SunFire^TM^ C18 column (250 × 10 mm) with 35–55%% acetonitrile to afford **1f–3f** and **1g–3g**.

#### 2-((4-Fluorobenzyl)oxy)-5'-methylene-hydrozinecarboximidamide-honokiol (1f)

Yellow solid, yield: 24.7%; ^1^H-NMR (400 MHz, CDCl_3_): *δ* 8.20 (s, 1H), 7.37–7.27 (m, 5H), 7.25–7.14 (s, 4H), 7.06–7.00 (m, 3H), 6.91 (s, 1H), 6.86 (d, *J* = 8.3 Hz, 1H), 6.04–5.76 (m, 2H), 5.06–4.95 (m, 6H), 3.41 (d, *J* = 6.6 Hz, 2H), 3.21 (d, *J* = 6.6 Hz, 2H). ^13^C-NMR (100 MHz, CDCl_3_): *δ* 163.58, 155.64, 155.58, 152.79, 151.90, 137.12, 136.79, 133.51, 132.98, 131.48, 130.91, 129.95, 129.89, 129.34, 128.97, 128.89, 128.78, 128.24, 117.58, 116.10, 115.68, 115.49, 115.28, 111.38, 69.33, 39.06, 34.60. HR-ESI-MS: molecular formula C_27_H_27_FN_4_O_2_ as revealed at *m/z* [M + H]^+^ 459.21939.

#### 2-((2-Fluorobenzyl)oxy)-5'-methylene-hydrozinecarboximidamide-honokiol (2f)

Brown solid, yield: 27.3%; ^1^H-NMR (600 MHz, CD_3_OD): *δ* 8.31 (s, 1H), 7.46 (d, *J* = 2.2 Hz, 1H), 7.43–7.40 (m, 1H), 7.38–7.29 (m, 2H), 7.16–7.05 (m, 5H), 6.03–5.92 (m, 2H), 5.13–4.96 (m, 6H), 3.45–3.41 (m, 2H), 3.40–3.36 (m, 2H). ^13^C-NMR (150 MHz, CD_3_OD): *δ* 161.08, 154.89, 153.49, 153.47, 150.91, 137.44, 135.83, 133.92, 132.91, 130.04, 129.93, 129.85, 129.41, 129.21, 127.92, 126.99, 123.98, 123.52, 116.78, 114.47, 114.43, 114.33, 114.09, 113.19, 64.15, 38.65, 33.09. HR-ESI-MS: molecular formula C_27_H_27_FN_4_O_2_ as revealed at *m/z* [M + H]^+^ 459.2207.

#### 2-((5-Fluorobenzyl)oxy)-5'-methylene-hydrozinecarboximidamide-honokiol (3f)

Yellow solid, yield: 27.3%; ^1^H-NMR (400 MHz, CDCl_3_): *δ* 8.22 (s, 1H), 7.33 (s, 4H), 7.36–7.21 (m, 4H), 7.18–7.06 (m, 3H), 7.03–6.81 (m, 3H), 6.09–5.77 (m, 2H), 5.13–4.86 (m, 6H), 3.45 (d, *J* = 6.5 Hz, 2H), 3.29–3.14 (m, 2H). ^13^C-NMR (100 MHz, CDCl_3_): *δ* 164.18, 155.52, 155.52, 152.78, 151.89, 139.93, 137.09, 136.74, 133.66, 131.56, 130.99, 130.08, 129.99, 129.95, 129.38, 128.80, 128.25, 122.40, 117.47, 116.14, 115.75, 114.72, 114.01, 111.34, 69.14, 39.06, 34.60. HR-ESI-MS: molecular formula C_27_H_27_FN_4_O_2_ as revealed at *m/z* [M + H]^+^ 459.2191.

#### 4'-((4-Fluorobenzyl)oxy)-3-methylene-hydrozinecarboximidamide-honokiol(1g)

Yellow solid, yield: 23.2%; ^1^H-NMR (400 MHz, DMSO-*d_6_*): *δ* 11.21 (s, 1H), 8.27 (s, 1H), 7.61–7.48 (m, 2H), 7.38 (dd, *J* = 8.4, 2.3 Hz, 1H), 7.34 (d, *J* = 2.3 Hz, 1H), 7.28–7.22 (m, 2H), 7.18 (d, *J* = 2.2 Hz, 1H), 7.09 (d, *J* = 8.5 Hz, 1H), 7.03 (d, *J* = 2.2 Hz, 1H), 6.64–6.16 (m, 4H), 6.04–5.93 (m, 2H), 5.16 (s, 2H), 5.14–4.97 (m, 4H), 3.41 (d, *J* = 6.6 Hz, 2H), 3.33 (d, *J* = 6.8 Hz, 2H). ^13^C-NMR (100 MHz, DMSO-*d_6_*): *δ* 163.35, 158.34, 155.27, 152.77, 148.51, 138.45, 137.35, 134.08, 131.14, 131.02, 130.83, 130.81, 129.94, 129.85, 128.85, 128.81, 128.66, 128.05, 120.39, 116.12, 116.07, 115.84, 115.62, 112.24, 69.04, 39.07, 34.62. HR-ESI-MS: molecular formula C_27_H_27_FN_4_O_2_ as revealed at *m/z* [M + H]^+^ 459.2192.

#### 4'-((2-Fluorobenzyl)oxy)-3-methylene-hydrozinecarboximidamide-honokiol(2g)

Yellow solid, yield: 20.3%; ^1^H-NMR (600 MHz, CD_3_OD): *δ* 8.37 (s, 1H), 7.58–7.55 (m, 1H), 7.38 (d, *J* = 2.3 Hz, 1H), 7.34 (t, *J* = 2.5 Hz, 2H), 7.22–7.13 (m, 4H), 7.09 (d, *J* = 8.3 Hz, 1H), 6.05 − 5.97 (m, 2H), 5.21 (s, 2H), 5.11–4.98 (m, 4H), 3.44 (d, *J* = 6.7 Hz, 2H), 3.38 (d, *J* = 6.7 Hz, 2H). ^13^C-NMR (150 MHz, CD_3_OD): *δ* 161.48, 155.58, 155.26, 152.26, 150.31, 137.46, 136.78, 133.60, 131.78, 130.75, 130.28, 129.92, 129.64, 129.58, 128.74, 128.58, 128.07, 123.99, 118.45, 114.90, 114.76, 114.69, 114.32, 111.42, 63.72, 38.69, 34.17. HR-ESI-MS: molecular formula C_27_H_27_FN_4_O_2_ as revealed at *m/z* [M + H]^+^ 459.2205.

#### 4'-((5-Fluorobenzyl)oxy)-3-methylene-hydrozinecarboximidamide-honokiol(3g)

Yellow solid, yield: 16.5%; ^1^H-NMR (400 MHz, DMSO-*d_6_*): *δ* 11.22 (s, 1H), 8.26 (s, 1H), 7.46 (td, *J* = 7.9, 6.0 Hz, 1H), 7.38 (dd, *J* = 8.4, 2.3 Hz, 1H), 7.35–7.28 (m, 3H), 7.19–7.14 (m, 2H), 7.06 (d, *J* = 8.5 Hz, 1H), 7.03 (d, *J* = 2.2 Hz, 1H), 6.27 (s, 4H), 6.06–5.93 (m, 2H), 5.21 (s, 2H), 5.13–5.01 (m, 4H), 3.44 (d, *J* = 6.6 Hz, 2H), 3.33 (d, *J* = 6.9 Hz, 3H). ^13^C-NMR (100 MHz, DMSO-*d_6_*): *δ* 163.90, 158.25, 155.13, 152.76, 148.50, 138.45, 137.37, 131.19, 131.10, 131.01, 130.93, 130.89, 130.85, 128.83, 128.69, 128.02, 123.54, 120.40, 116.12, 116.09, 115.01, 114.34, 114.12, 112.16, 68.84, 45.94, 34.63. HR-ESI-MS: molecular formula C_27_H_27_FN_4_O_2_ as revealed at *m/z* [M + H]^+^ 459.2210.

### Cell culture

The human nasopharyngeal carcinoma cell line (CNE-2Z), gastric cancer cell line (SGC7901), breast cancer cell line (MCF-7), mouse leydig testicular cancer cell line (I-10), and mouse leydig cell line (TM3) were obtained from the Chinese Academy of Sciences Cell Bank (Shanghai, China). The cells were cultured in Dulbecco’s modified Eagle’s medium (DMEM, Hyclone, USA) supplemented with 10% foetal bovine serum (FBS, Hangzhou Sijiqing Co., Ltd., China) and 1% penicillin/streptomycin (Gibco, USA). Cell culture was conducted in an incubator at 37 °C and 5% CO_2_.

### MTT assay

All the cell lines were incubated in 96-well plates at a density of 5 × 10^3^ cells per well and cultured overnight at 37 °C. After adherence, the cells were treated with various concentrations of the test compounds for 24, 48, and 72 h. After the mentioned time points, cell viability was examined using a standard MTT assay. The cytotoxic activity of compounds was expressed as IC_50_ value that inhibited cell growth to 50% of control. Cis-platinum (DDP) was used as the positive control.

### Colony-formation assay

CNE-2Z cells were seeded in 6-well plates at a density of 6** **×** **10^3^ cells/well and cultured overnight. When colonies formed, the medium was replaced with fresh medium containing **1g** at various concentrations (0, 0.5, 1, and 2** **μM), and the cells were cultured for 7** **days. After 7** **days, the cells were washed with phosphate buffer saline (PBS), fixed with 4% paraformaldehyde for 10 min, followed by stained with crystal violet for 10 min and photographed.

### Cell migration assay

The migration assay was assessed using a 24-well plate with 8.0 μm pore membrane inserts (Corning, NY) without matrigel. CNE-2Z cells were added to the upper chambers at a concentration of 5 × 10^4^/well, and incubated for 24 h, followed by treated with various concentrations (0, 1, 3, and 6 μM) of **1g**. The lower chambers were filled with the conditioned media. After 24 h, the cells that had migrated were stained with 0.1% crystal violet and photographed under a light microscope at 200× magnification. The number of migratory cells was counted and analysed to determine statistically significant differences. The migration assay was performed independently three times. DDP was used as a positive control.

### Cell invasion assay

The invasion assay was assessed using a 24-well plate with 8.0 μm pore membrane inserts that were coated with 50 μl of matrigel (BD, USA) and incubated at 37 °C for 1 h. CNE-2Z cells (5 × 10^4^/well) were added to the upper chambers and incubated with various concentrations (0, 1, 3, and 6 μM) of **1g** for 36 h. The remainder of the procedure was same as that described for the cell migration experiment.

### Western blotting

CNE-2Z cells were cultured in 6-well plates at a density of 6** **×** **10^5^ cells/well. After adherence, the cells were treatment with various concentrations of **1g** (0, 2.5, 5, and 10 μM) and incubated for 24 h. The cells were harvested, washed with PBS, and proteins were extracted and quantified. Proteins were separated by sodium dodecyl sulfate polyacrylamide gel electrophoresis (SDS-PAGE) and transferred onto polyvinylidene fluoride (PVDF) membranes. The PVDF membrane was blocked with 5% skimmed milk and incubated with primary antibodies at 4 °C for overnight. After washing with TBST buffer, the membranes were incubated with the corresponding secondary antibodies at room temperature for 2.5 h. Finally, the protein bands were visualized using a chemiluminescence kit and detected using a gel imaging system (Bio-Rad, USA). Anti-β-actin was used as an internal control.

### Small interfering RNA (siRNA) transfection

HIF-1a siRNAs were purchased from Gene-Pharma (China) and were transiently transfected into CNE-2Z cells in 6-well plates using Lipofectamine 2000 reagent (Invitrogen, USA). After 48 h of transfection, the cells were collected for western blotting as described previously. The siRNA sequences used for experiments with human HIF-1a were as follows: Negative control sense, 5′-UUC UCC GAA CGU GUC ACG UTT-3′ and antisense, 5′-ACG UGA CAC GUU CGG AGA ATT-3′; Positive control sense, 5′-UGA CCU CAA CUA CAU GGU UTT-3′ and antisense, 5′-AAC CAU GUA GUU GAG GUC ATT-3′; HIF-1a homo 2107 sense, 5′-CCA GCA GAC UCA AAU ACA ATT-3′ and antisense, 5′-UUG UAU UUG AGU CUG CUG GTT-3′.

### Flow cytometry with Annexin V/PI staining

CNE-2Z cells were cultured in a 12-well plate at a density of 2.5 × 10^5^ cells per well and incubated overnight. After adherence, the cells were treated with various concentrations of **1g** (0, 2.5, 5, and 10 μM), and cultured for 24 h. The cells were harvested from each well, washed with PBS, and stained with Annexin V-FITC solution, followed by the addition of PI staining solution and subsequent incubation at room temperature in the dark for 12 min. The percentage of apoptotic cells was analyzed using flow cytometry (BD Biosciences, USA).

### In vivo anti-tumour experiments

To evaluate whether anti-tumour efficacy was delivered by **1g**, we used nude mice (female BALB/c nude mice, 4–5 weeks of age, obtained from Canes Laboratory, Changzhou, China) to perform experiment. CNE-2Z cells (5 × 10^6^ cells/animal) were injected subcutaneously to induce tumour formation. All experimental procedures and protocols were performed in accordance with the National Institutes of Health Guidelines for the Care and Use of Laboratory Animals. When approximately 100 mm^3^ of tumour volume was measured, the mice were randomly divided into three groups (four mice per group), Vehicle control, **1g** (10 mg/kg), and DDP (3 mg/kg) injected intraperitoneally every 3 days for 19 days. DDP was used as a positive control drug. Tumour volume (calculated as tumour length × width^2^/2) and body weight were monitored every 3 days. After treatment for 19 days, the mice were sacrificed, and serum glutamate oxaloacetate transaminase (GOT) and glutamate pyruvate transaminase (GPT) levers were measured. In addition, the liver, kidneys, lungs, and solid tumours were removed, preserved in 4% formalin solution, that were stained with haematoxylin and eosin (H&E).

### Statistical analysis

Data are presented as the means ± SD from three independent experiments. SPSS software (version 16.0; SPSS Inc., Chicago, IL, USA) was used for the data analysis. Statistical Comparisons between groups were performed using one-way ANOVA followed by the Student’s *t*-test. *P < 0.05, **P < 0.01 were considered statistically significant.

## Supplementary Material

Supplemental Material
