# Supplementary material for: Semisynthesis and anti-cancer properties of novel honokiol derivatives in human nasopharyngeal carcinoma CNE-2Z cells
Source: J Enzyme Inhib Med Chem. 2023 Aug 9;38(1):2244694. doi: 10.1080/14756366.2023.2244694 (PMC10413922; doi:10.1080/14756366.2023.2244694)
Supplement: Supplemental Material [file IENZ_A_2244694_SM0419.pdf]

Supplementary materials for

Semisynthesis and anti-cancer properties of novel honokiol derivatives in human nasopharyngeal carcinoma CNE-2Z cells

Bo-Han Li[a†], Hui Ma[a†], Jing Zhu[a], Jie Chen[a], Yi-Qun Dai[a,b], Xiao-Jing Zhang[c], Hong-Mei Li[a,b]* and Cheng-Zhu Wu [a,b]*

[a] School of Pharmacy, Bengbu Medical College, Bengbu 233030, Anhui, China

[b] Anhui Province Biochemical Pharmaceutical Engineering Technology Research Center, Bengbu 233030, Anhui, China.

[c] Department of Surgical Oncology, The First Affiliated Hospital of Bengbu Medical College, Bengbu 233000, Anhui, China

* Corresponding authors: lihongmei@bbmc.edu.cn (Li HM);wuchengzhu0611@bbmc. edu.cn (Wu CZ).

[†] These authors contributed equally to the work.

List of supplemental data

Figure S1 ^{1}H-NMR (400 MHz, $CDCl_3$) spectrum of **1a** 4
Figure S2 ^{13}C-NMR (100 MHz, $CDCl_3$) spectrum of **1a** 4
Figure S3 HR-ESI-MS spectrum of **1a** 5
Figure S4 ^{1}H-NMR (400 MHz, $CDCl_3$) spectrum of **2a** 5
Figure S5 ^{13}C-NMR (100 MHz, $CDCl_3$) spectrum of **2a** 6
Figure S6 HR-ESI-MS spectrum of **2a** 6
Figure S7 ^{1}H-NMR (400 MHz, $CDCl_3$) spectrum of **3a** 7
Figure S8 ^{13}C-NMR (100 MHz, $CDCl_3$) spectrum of **3a** 7
Figure S9 HR-ESI-MS spectrum of **3a** 8
Figure S10 ^{1}H-NMR (400 MHz, $CDCl_3$) spectrum of **1b** 8
Figure S11 ^{13}C-NMR (100 MHz, $CDCl_3$) spectrum of **1b** 9
Figure S12 HR-ESI-MS spectrum of **1b** 9
Figure S13 ^{1}H-NMR (600 MHz, CD_3OD) spectrum of **2b** 10
Figure S14 ^{13}C-NMR (150 MHz, CD_3OD) spectrum of **2b** 10
Figure S15 HR-ESI-MS spectrum of **2b** 11
Figure S16 ^{1}H-NMR (600 MHz, CD_3OD) spectrum of **3b** 11
Figure S17 ^{13}C-NMR (150 MHz, CD_3OD) spectrum of **3b** 12
Figure S18 HR-ESI-MS spectrum of **3b** 12
Figure S19 ^{1}H-NMR (600 MHz, CD_3OD) spectrum of **1c** 13
Figure S20 ^{13}C-NMR (150 MHz, CD_3OD) spectrum of **1c** 13
Figure S21 HR-ESI-MS spectrum of **1c** 14
Figure S22 ^{1}H-NMR (600 MHz, CD_3OD) spectrum of **2c** 14
Figure S23 ^{13}C-NMR (150 MHz, CD_3OD) spectrum of **2c** 15
Figure S24 HR-ESI-MS spectrum of **2c** 15
Figure S25 ^{1}H-NMR (600 MHz, CD_3OD) spectrum of **3c** 16
Figure S26 ^{13}C-NMR (150 MHz, CD_3OD) spectrum of **3c** 16
Figure S27 HR-ESI-MS spectrum of **3c** 17
Figure S28 ^{1}H-NMR (400 MHz, $CDCl_3$) spectrum of **1d** 17
Figure S29 ^{13}C-NMR (100 MHz, $CDCl_3$) spectrum of **1d** 18
Figure S30 HR-ESI-MS spectrum of **1d** 18
Figure S31 ^{1}H-NMR (400 MHz, $CDCl_3$) spectrum of **2d** 19
Figure S32 ^{13}C-NMR (100 MHz, $CDCl_3$) spectrum of **2d** 19
Figure S33 HR-ESI-MS spectrum of **2d** 20
Figure S34 ^{1}H-NMR (400 MHz, $CDCl_3$) spectrum of **3d** 20
Figure S35 ^{13}C-NMR (100 MHz, $CDCl_3$) spectrum of **3d** 21
Figure S36 HR-ESI-MS spectrum of **3d** 21
Figure S37 ^{1}H-NMR (600 MHz, CD_3OD) spectrum of **1e** 22
Figure S38 ^{13}C-NMR (150 MHz, CD_3OD) spectrum of **1e** 22
Figure S39 HR-ESI-MS spectrum of **1e** 23
Figure S40 ^{1}H-NMR (400 MHz, $CDCl_3$) spectrum of **2e** 23
Figure S41 ^{13}C-NMR (100 MHz, $CDCl_3$) spectrum of **2e** 24
Figure S42 HR-ESI-MS spectrum of **2e** 24

Figure S43 ^{1}H-NMR (400 MHz, $CDCl_3$) spectrum of **3e** 25
Figure S44 ^{13}C-NMR (100 MHz, $CDCl_3$) spectrum of **3e** 25
Figure S45 HR-ESI-MS spectrum of **3e** 26
Figure S46 ^{1}H-NMR (400 MHz, $CDCl_3$) spectrum of **1f** 26
Figure S47 ^{13}C-NMR (100 MHz, $CDCl_3$) spectrum of **1f** 27
Figure S48 HR-ESI-MS spectrum of **1f** 27
Figure S49 ^{1}H-NMR (600 MHz, CD_3OD) spectrum of **2f** 28
Figure S50 ^{13}C-NMR (150 MHz, CD_3OD) spectrum of **2f** 28
Figure S51 HR-ESI-MS spectrum of **2f** 29
Figure S52 ^{1}H-NMR (400 MHz, $CDCl_3$) spectrum of **3f** 29
Figure S53 ^{13}C-NMR (100 MHz, $CDCl_3$) spectrum of **3f** 30
Figure S54 HR-ESI-MS spectrum of **3f** 30
Figure S55 ^{1}H-NMR (400 MHz, DMSO-d_6) spectrum of **1g** 31
Figure S56 ^{13}C-NMR (100 MHz, DMSO-d_6) spectrum of **1g** 31
Figure S57 HR-ESI-MS spectrum of **1g** 32
Figure S58 ^{1}H-NMR (600 MHz, CD_3OD) spectrum of **2g** 32
Figure S59 ^{13}C-NMR (150 MHz, CD_3OD) spectrum of **2g** 33
Figure S60 HR-ESI-MS spectrum of **2g** 33
Figure S61 ^{1}H-NMR (400 MHz, DMSO-d_6) spectrum of **3g** 34
Figure S62 ^{13}C-NMR (100 MHz, DMSO-d_6) spectrum of **3g** 34
Figure S63 HR-ESI-MS spectrum of **3g** 35

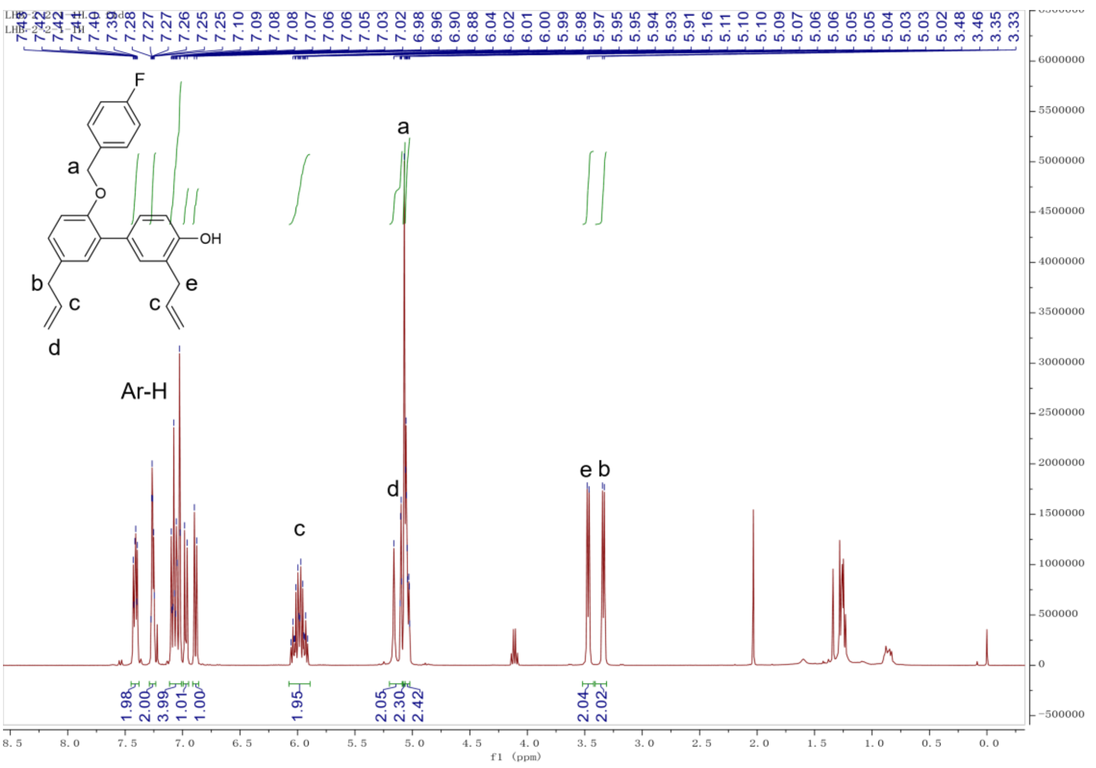

Figure S1. ^{1}H-NMR (400 MHz, $CDCl_3$) spectrum of **1a**.

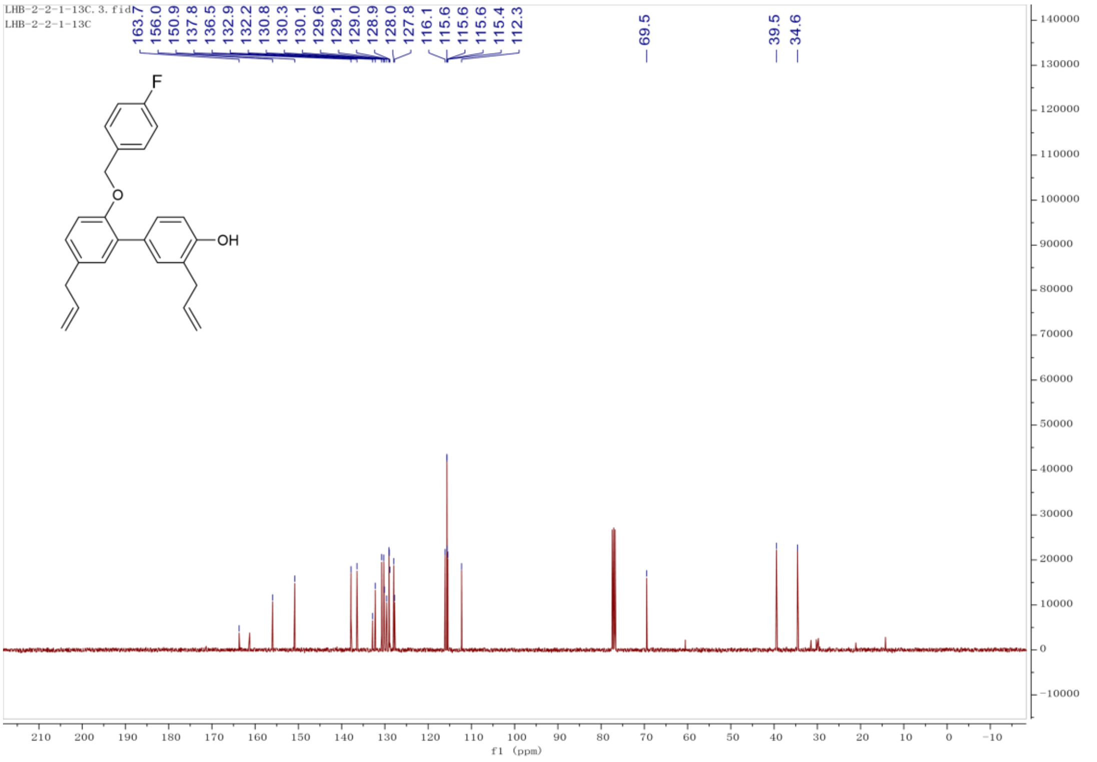

Figure S2. ^{13}C-NMR (100 MHz, $CDCl_3$) spectrum of **1a**.

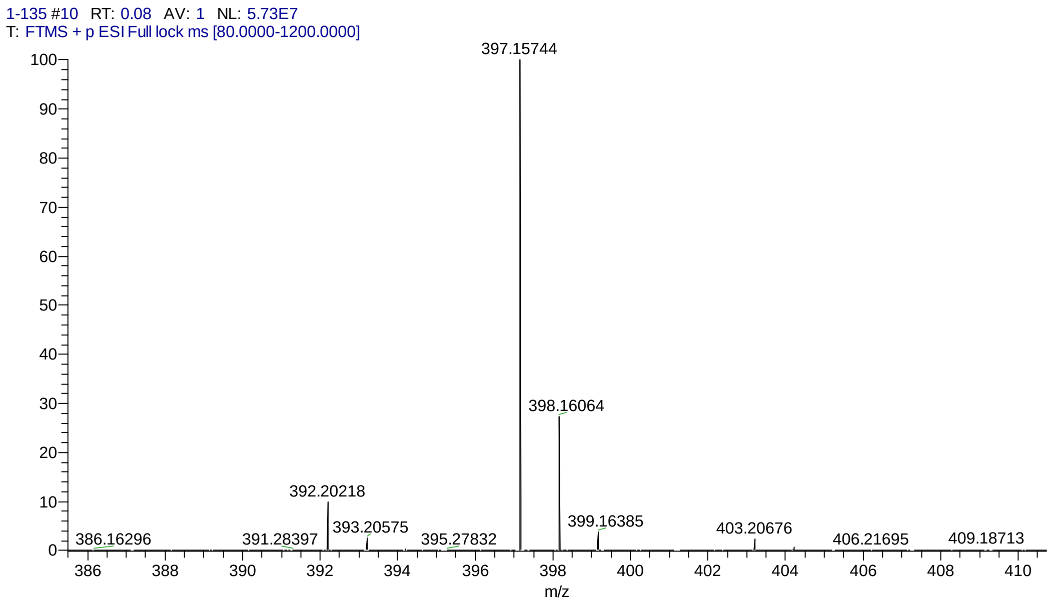

Figure S3. HR-ESI-MS spectrum of **1a.**

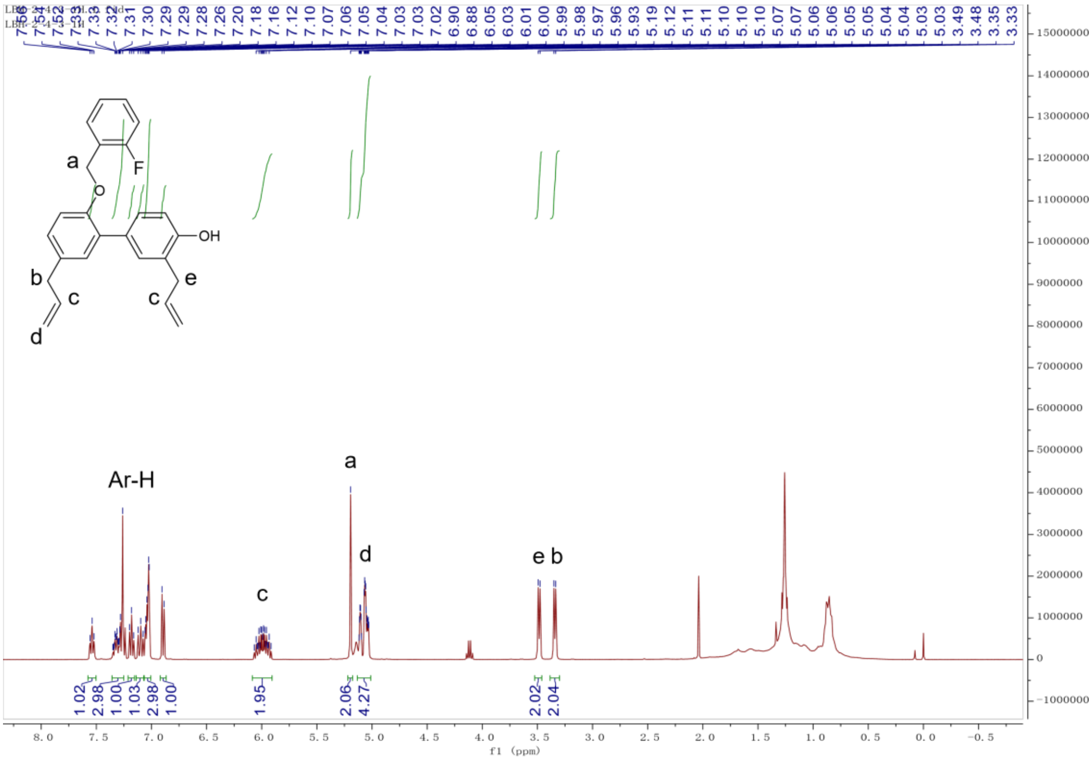

Figure S4. ^{1}H-NMR (400 MHz, $CDCl_3$) spectrum of **2a**.

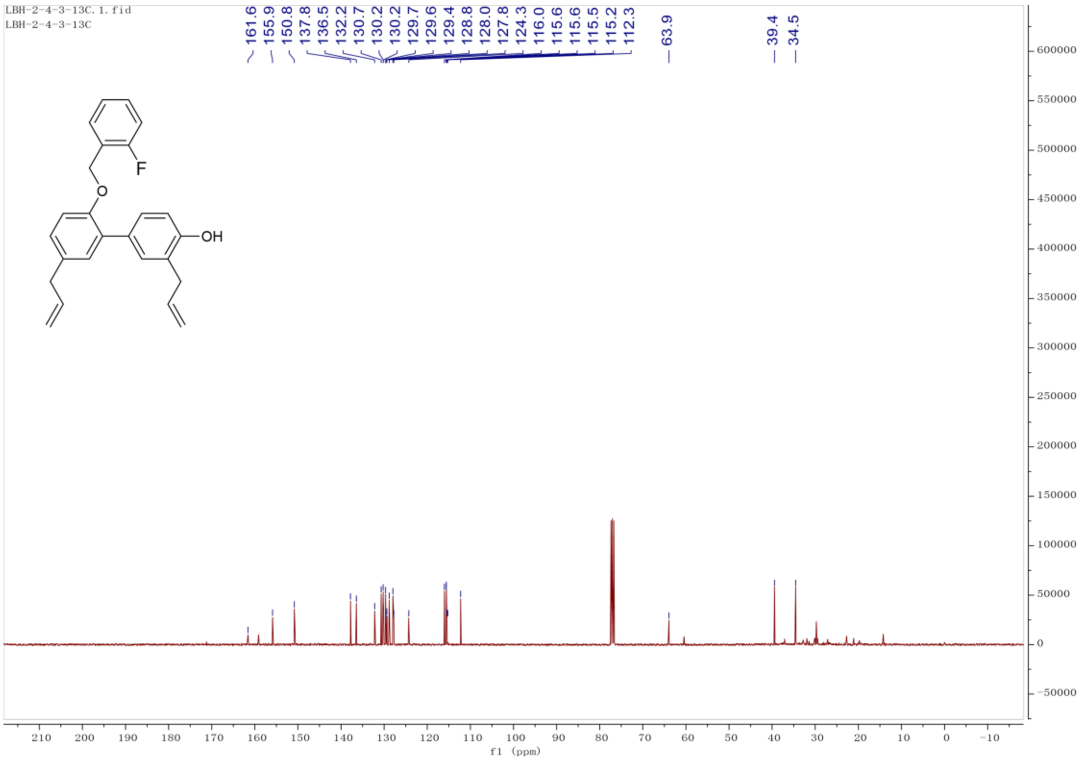

Figure S5. ^{13}C-NMR (100 MHz, $CDCl_3$) spectrum of **2a**.

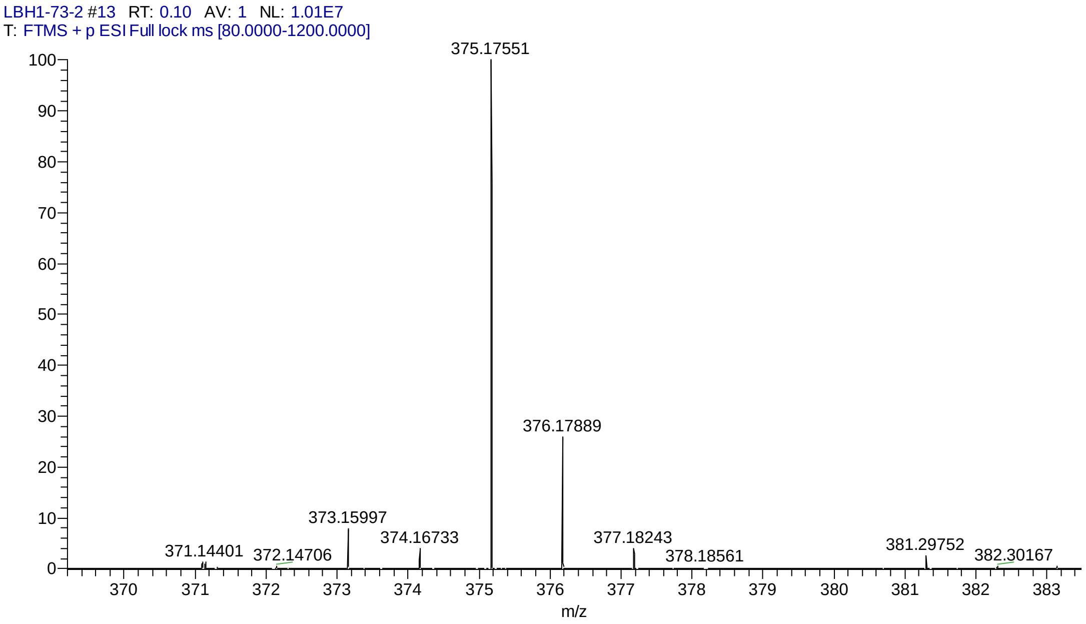

Figure S6. HR-ESI-MS spectrum of **2a.**

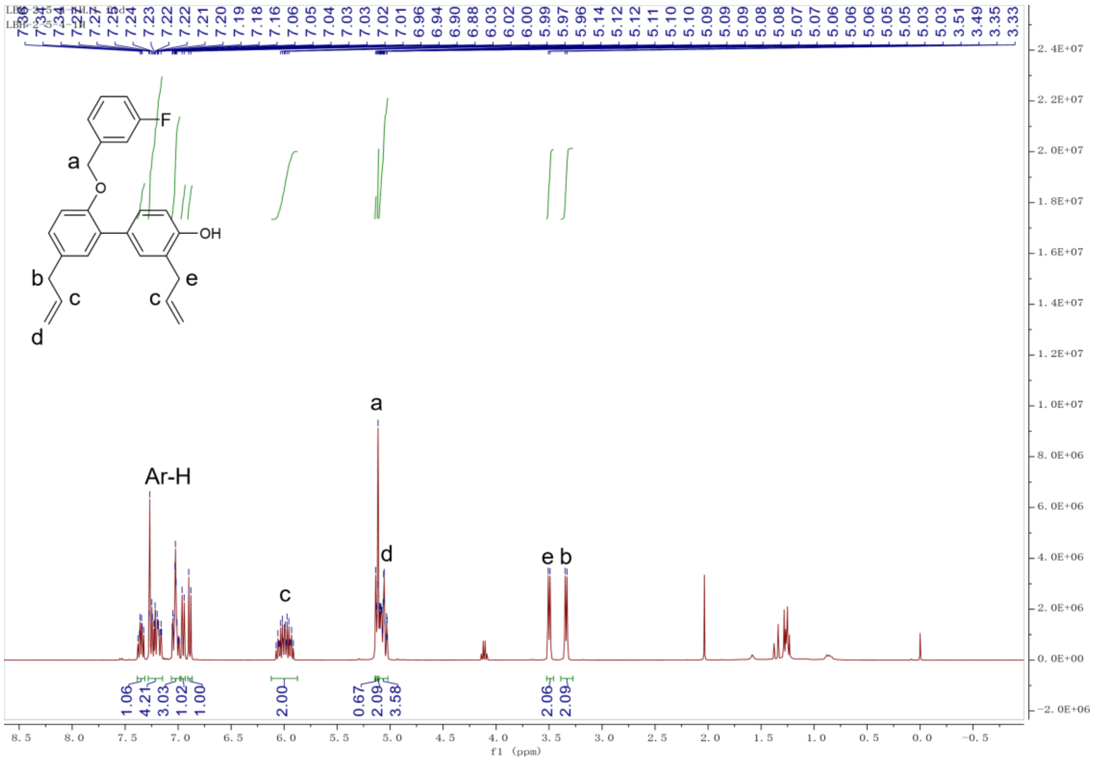

Figure S7. ^{1}H-NMR (400 MHz, $CDCl_3$) spectrum of **3a**.

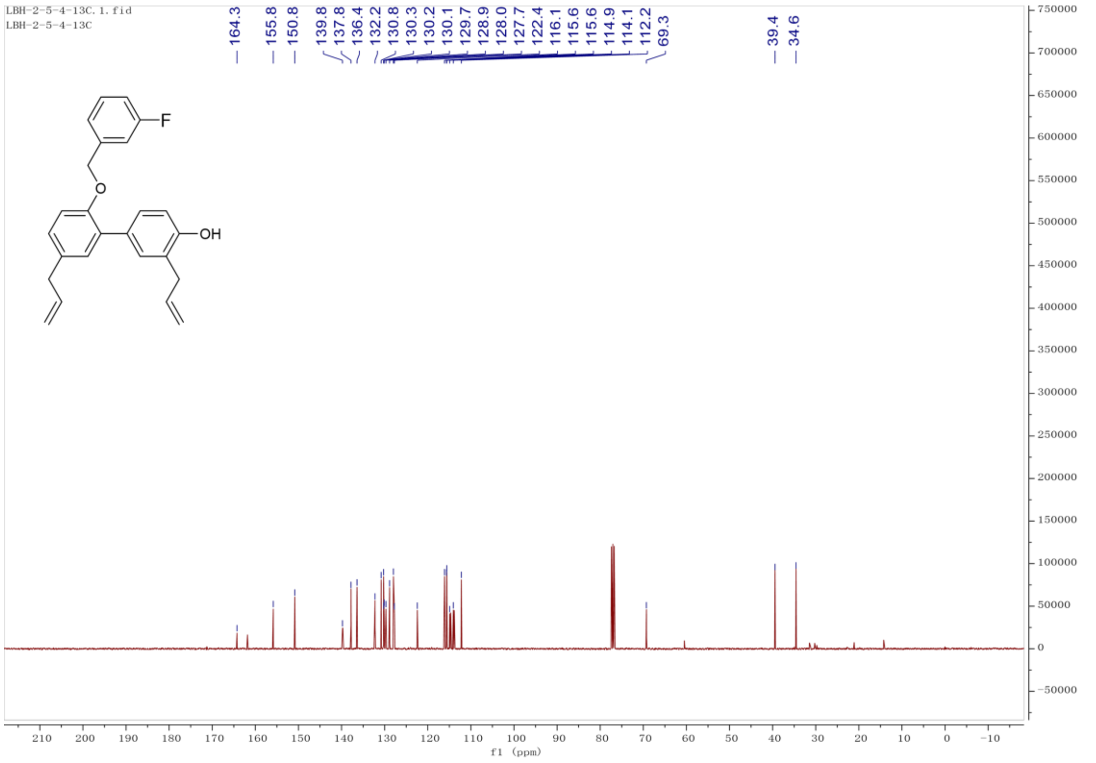

Figure S8. ^{13}C-NMR (100 MHz, $CDCl_3$) spectrum of **3a**.

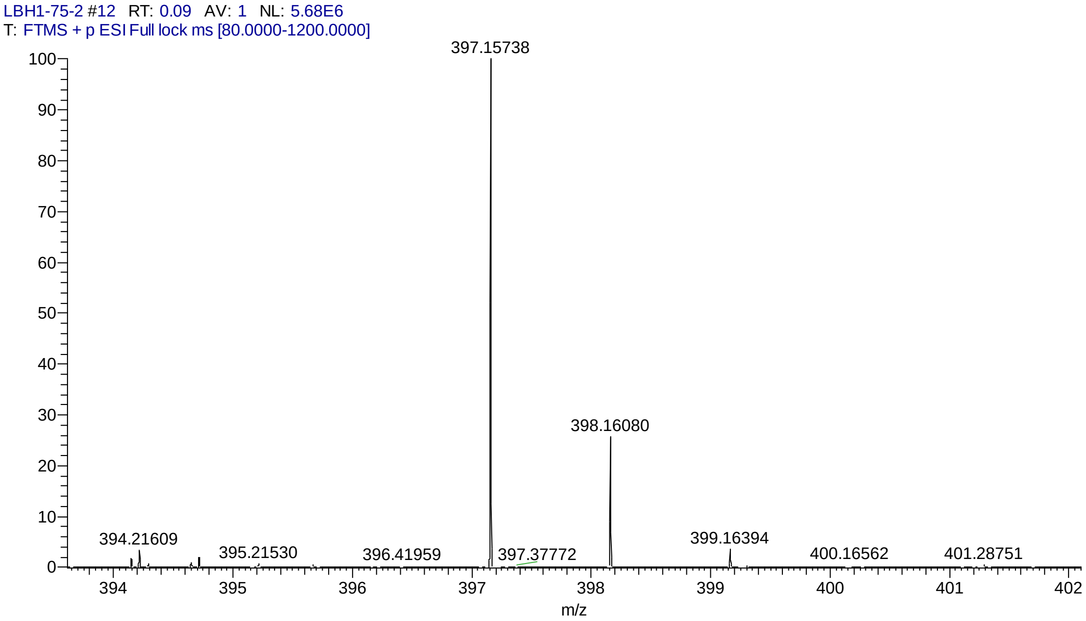

Figure S9. HR-ESI-MS spectrum of **3a.**

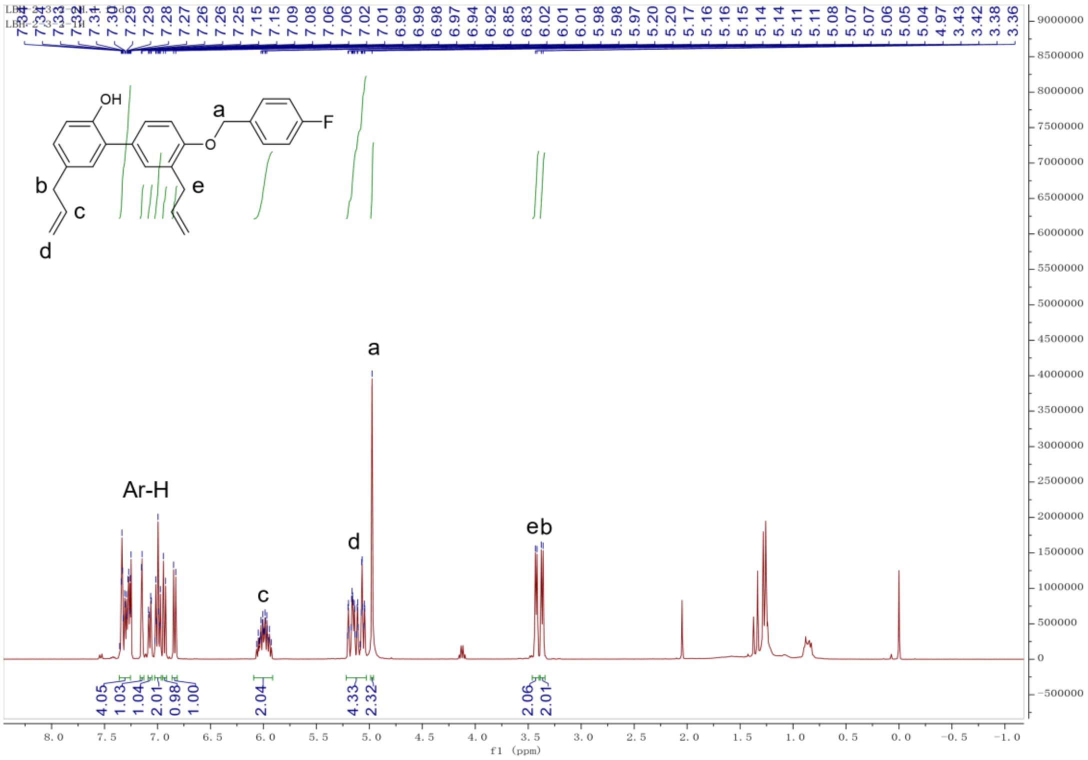

Figure S10. ^{1}H-NMR (400 MHz, $CDCl_3$) spectrum of **1b**.

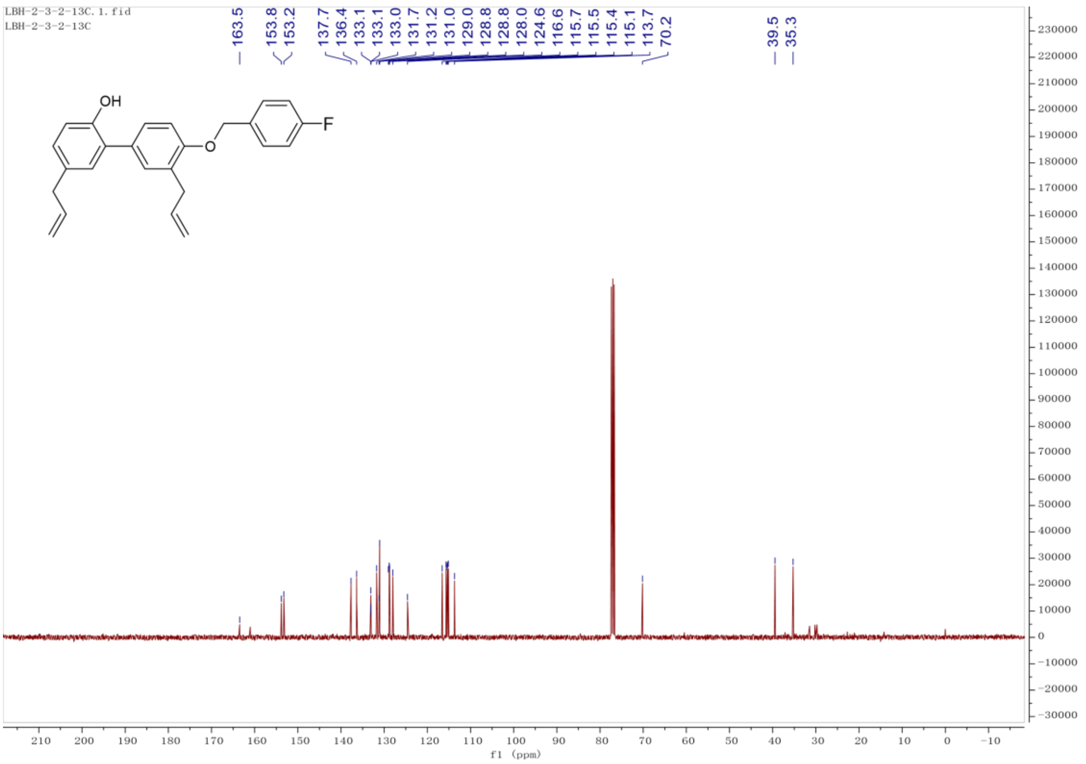

Figure S11. ^{13}C-NMR (100 MHz, $CDCl_3$) spectrum of **1b**.

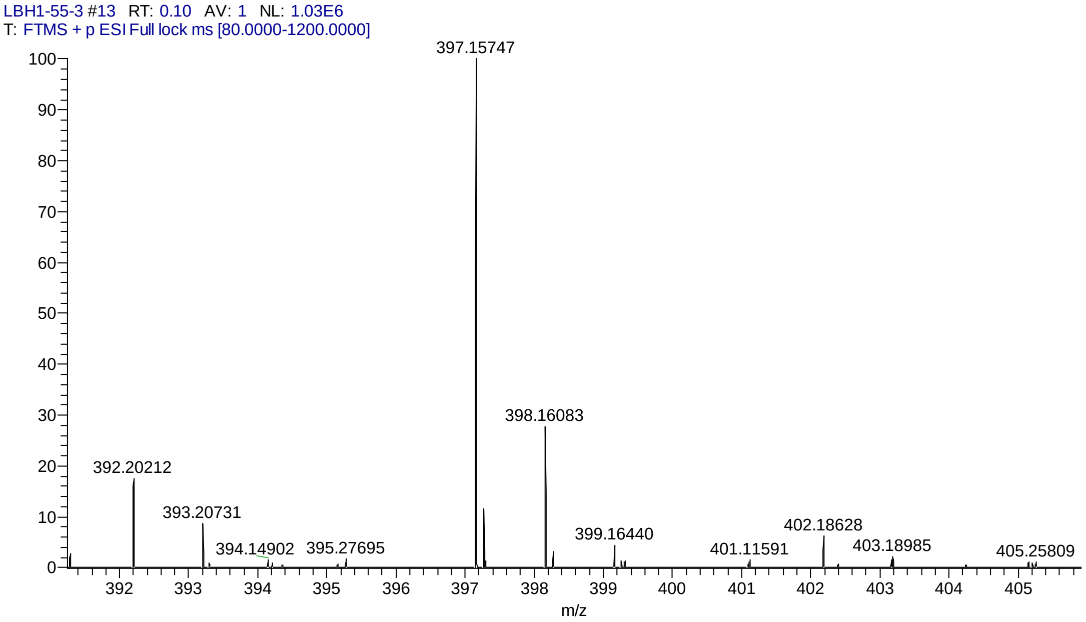

Figure S12. HR-ESI-MS spectrum of **1b.**

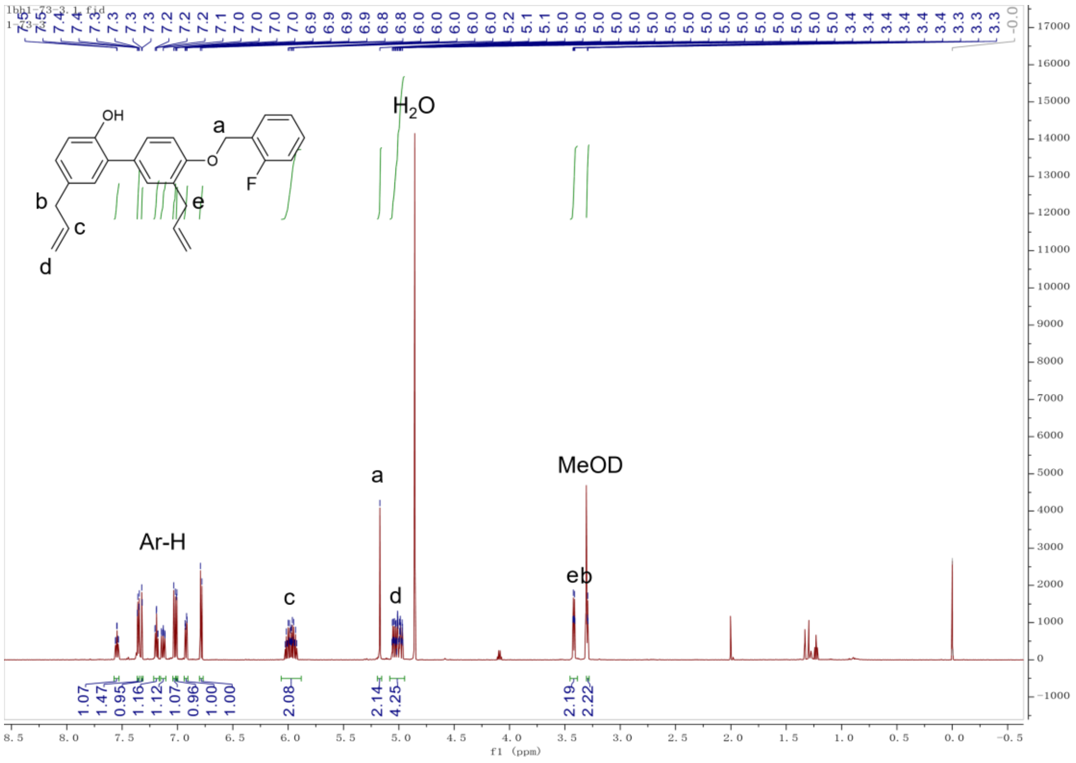

Figure S13. ^{1}H-NMR (600 MHz, CD_3OD) spectrum of **2b**.

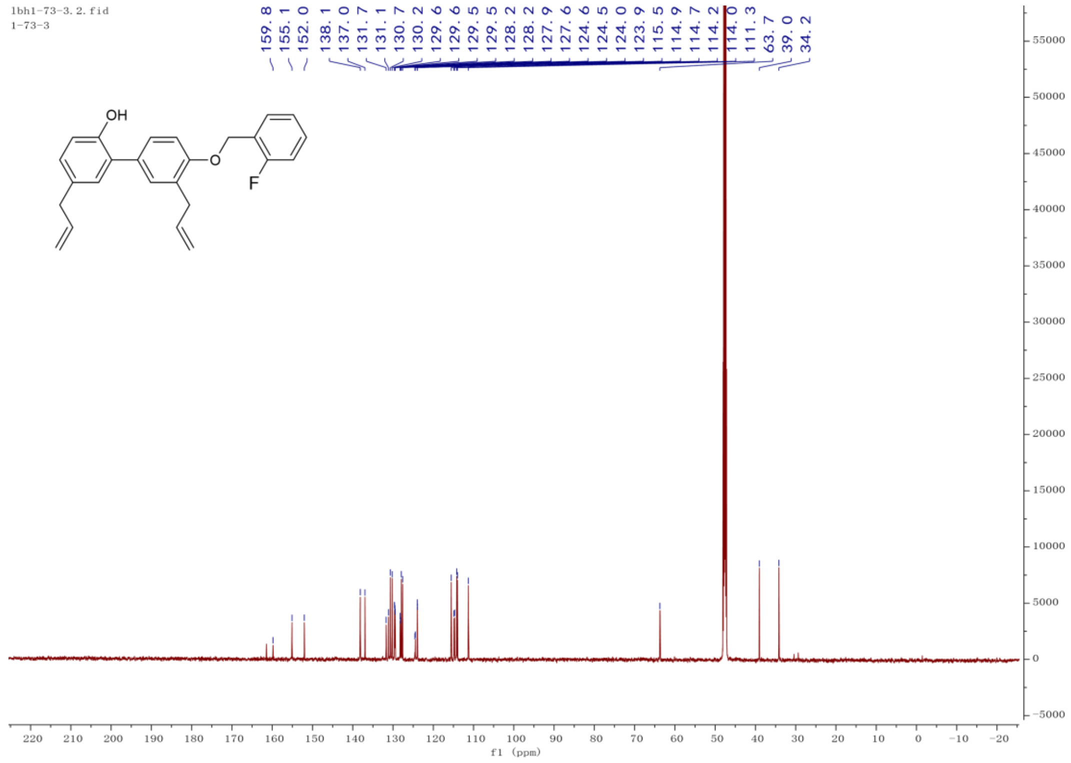

Figure S14. ^{13}C-NMR (150 MHz, CD_3OD) spectrum of **2b**.

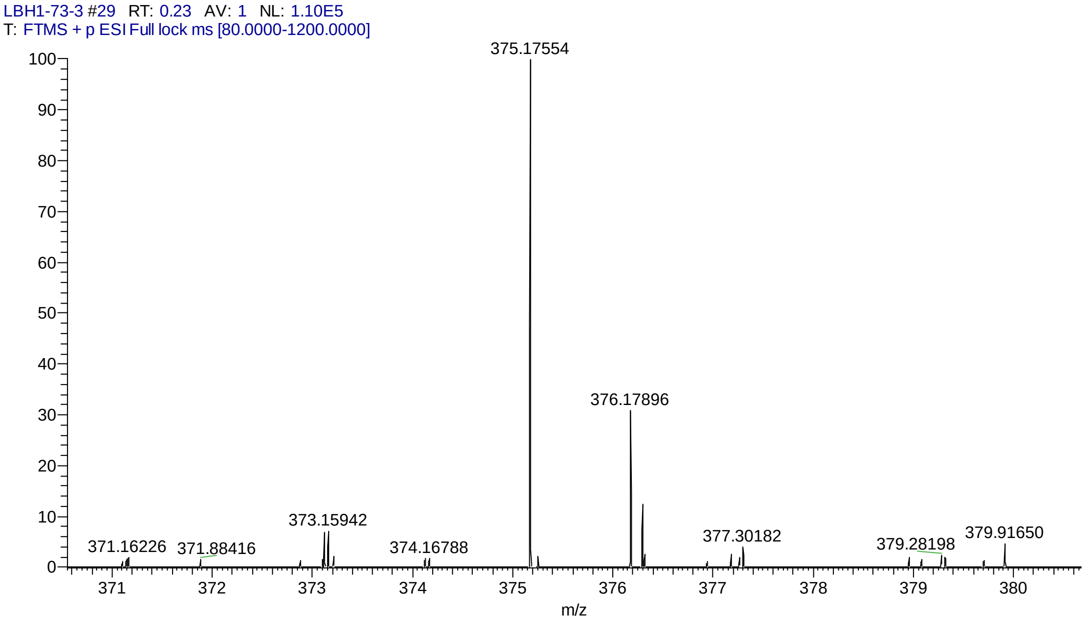

Figure S15. HR-ESI-MS spectrum of **2b.**

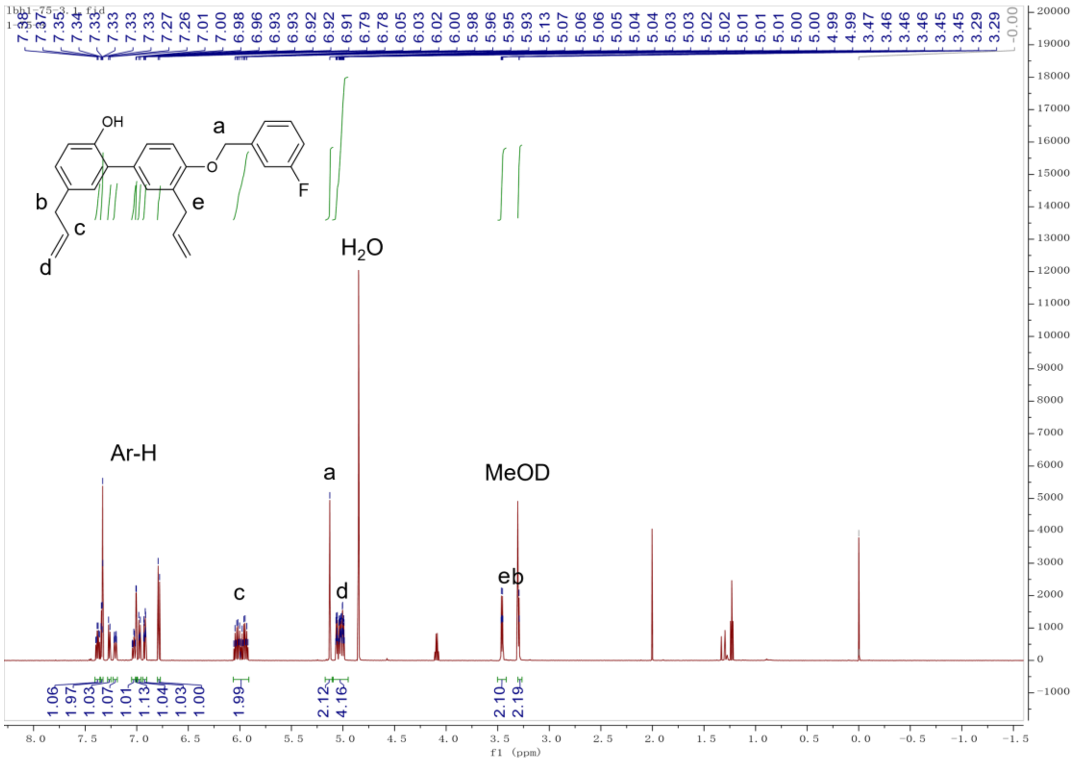

Figure S16. ^{1}H-NMR (600 MHz, CD_3OD) spectrum of **3b**.

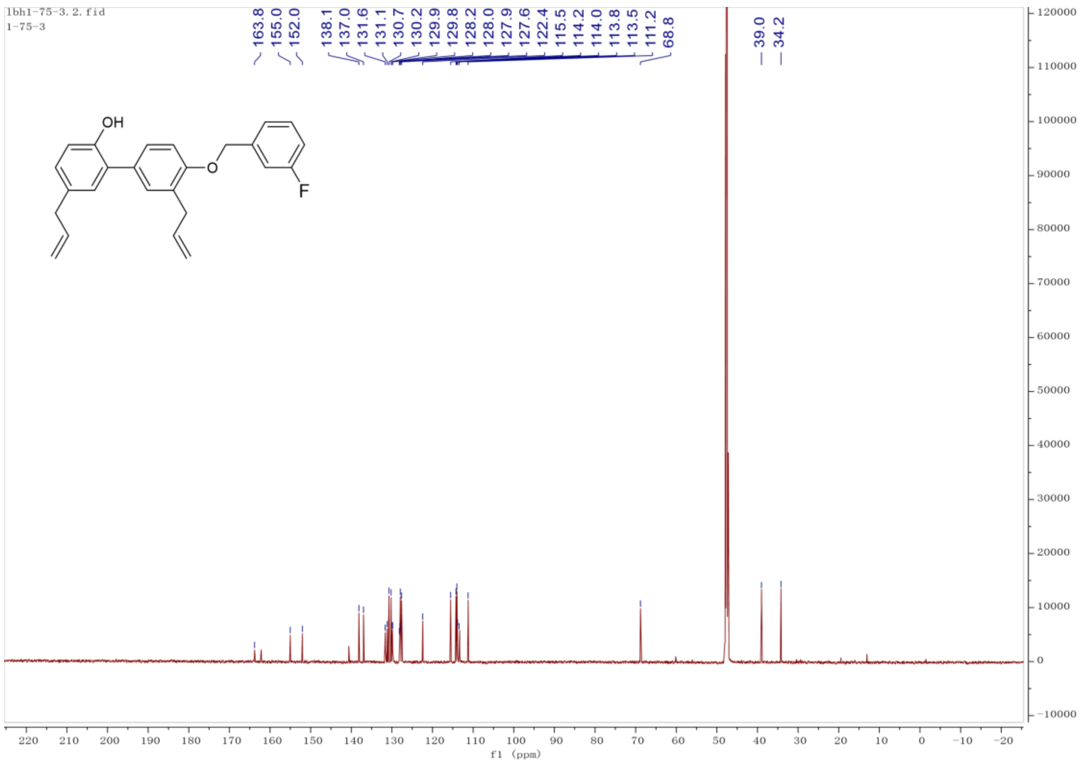

Figure S17. ^{13}C-NMR (150 MHz, CD_3OD) spectrum of **3b**.

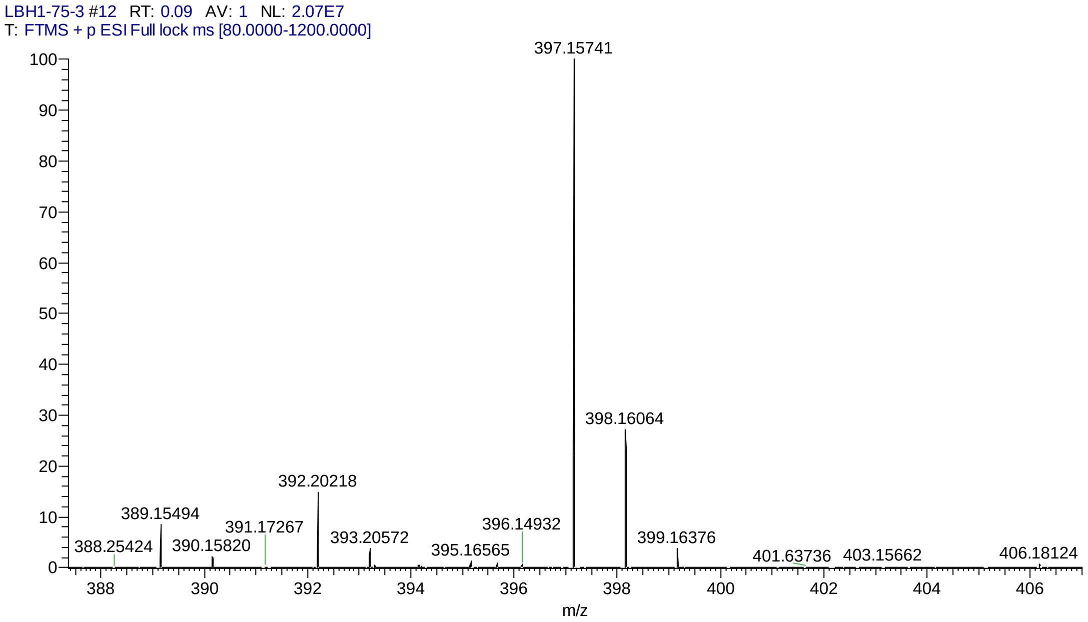

Figure S18. HR-ESI-MS spectrum of **3b.**

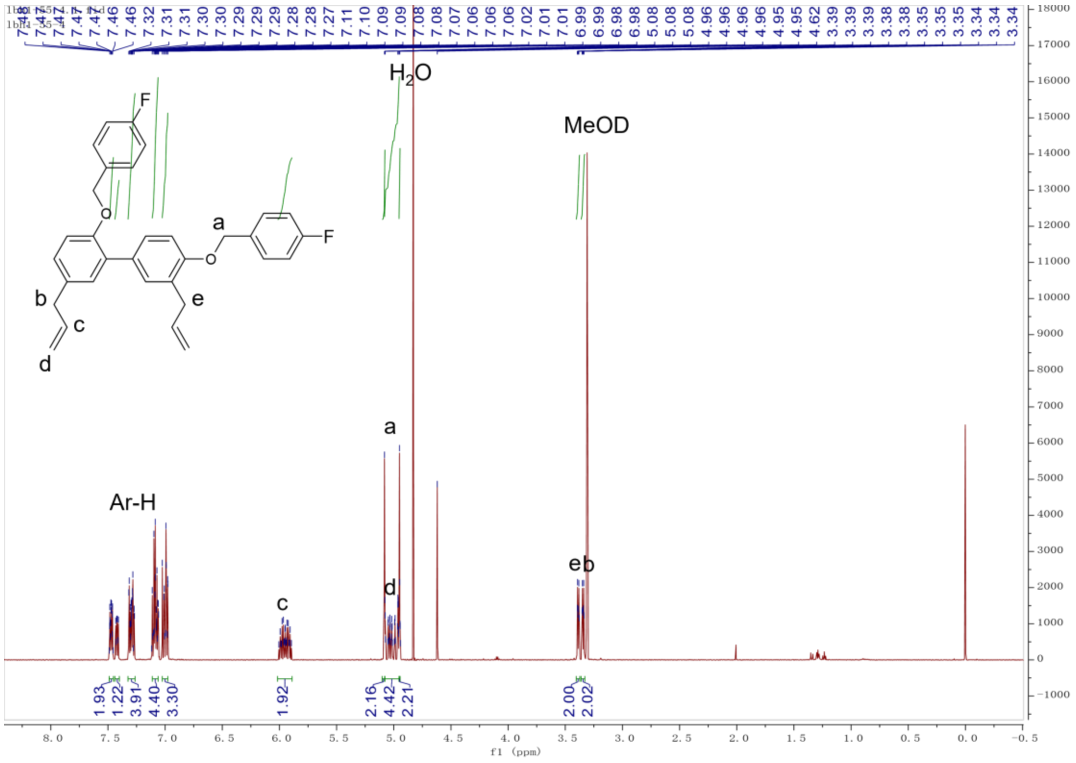

Figure S19. ^{1}H-NMR (600 MHz, CD_3OD) spectrum of **1c**.

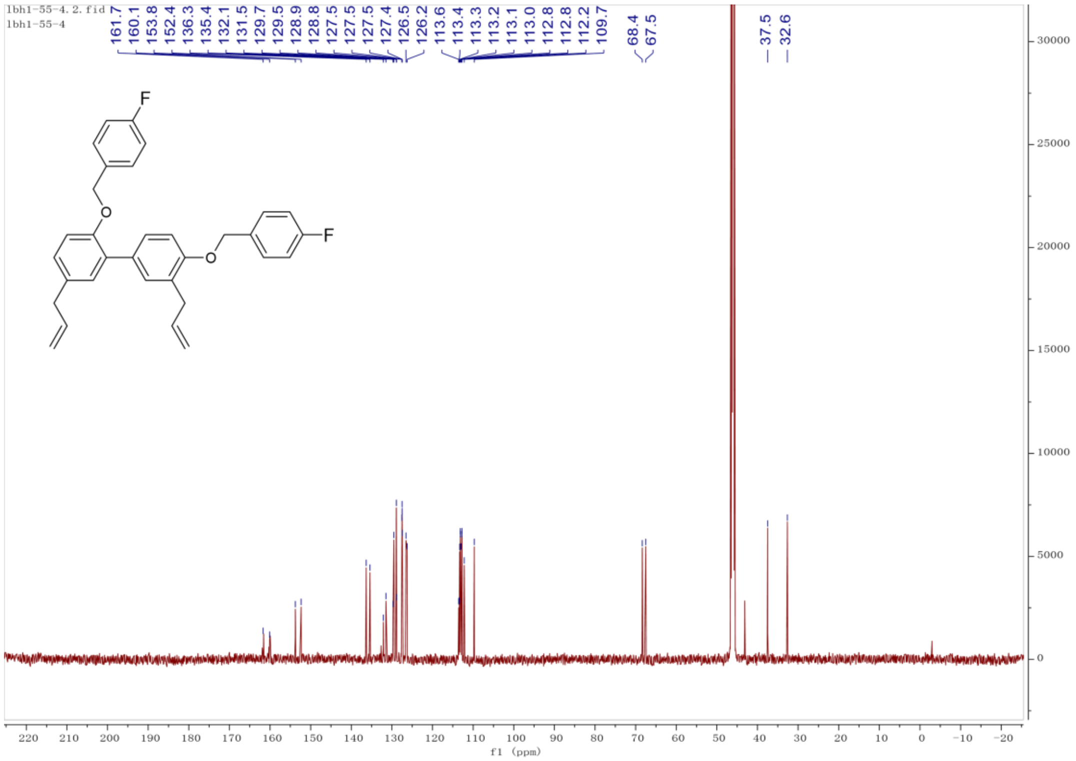

Figure S20. ^{13}C-NMR (150 MHz, CD_3OD) spectrum of **1c**.

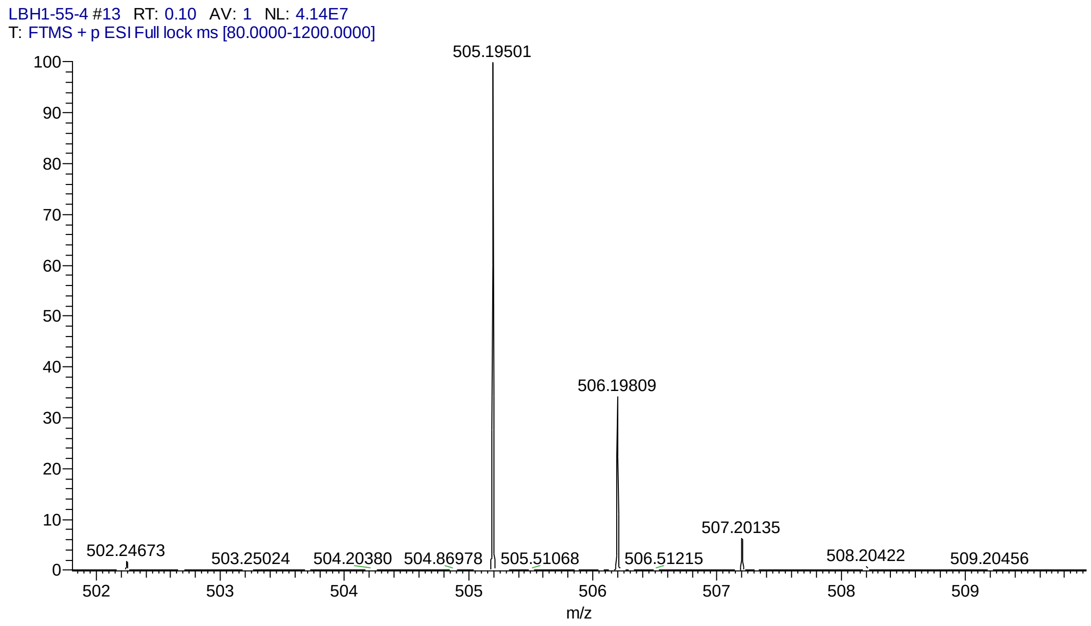

Figure S21. HR-ESI-MS spectrum of **1c.**

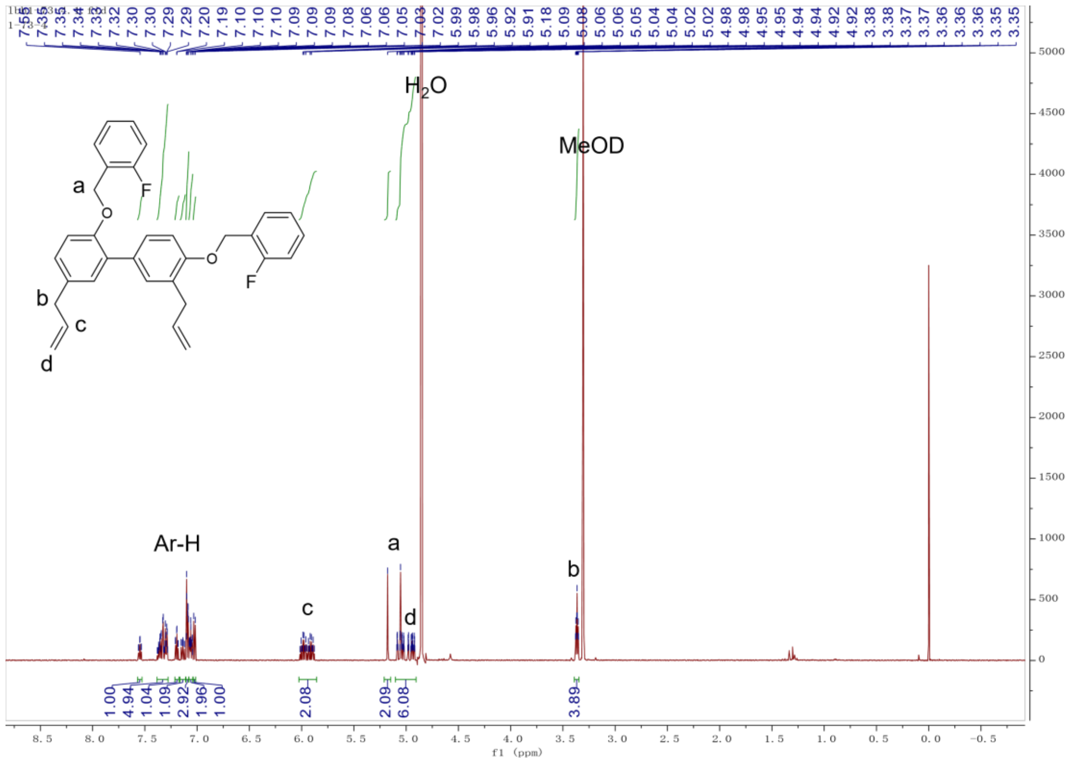

Figure S22. ^{1}H-NMR (600 MHz, CD_3OD) spectrum of **2c**.

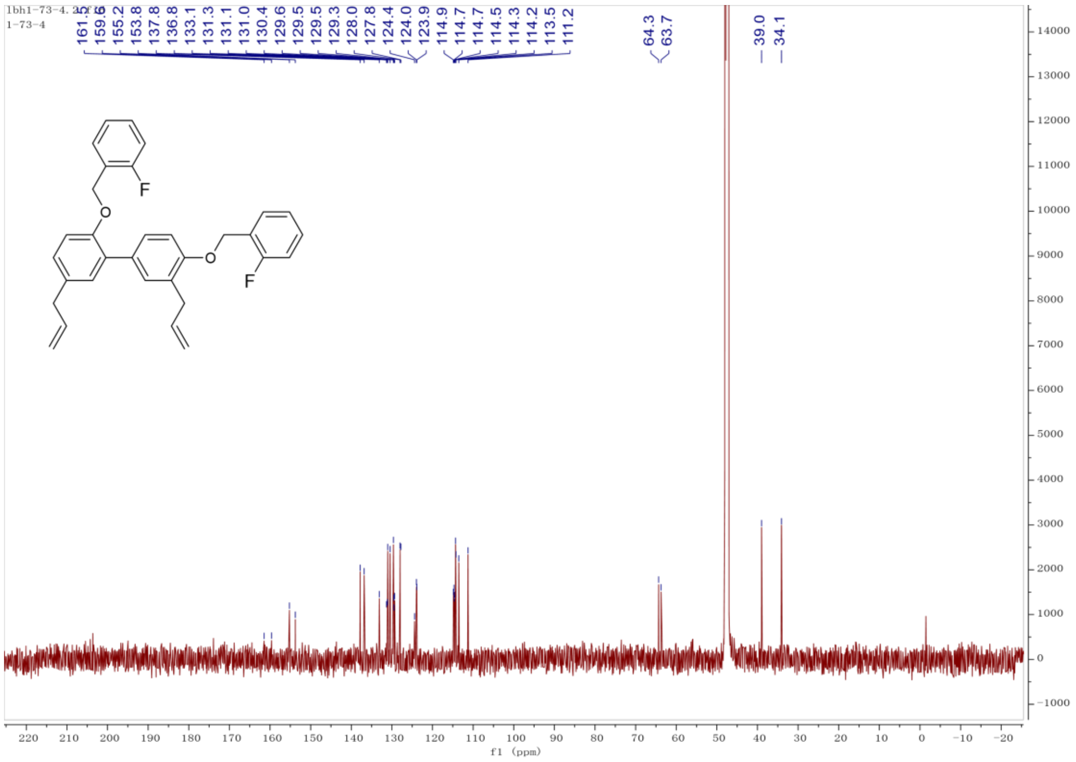

Figure S23. ^{13}C-NMR (150 MHz, CD_3OD) spectrum of **2c**.

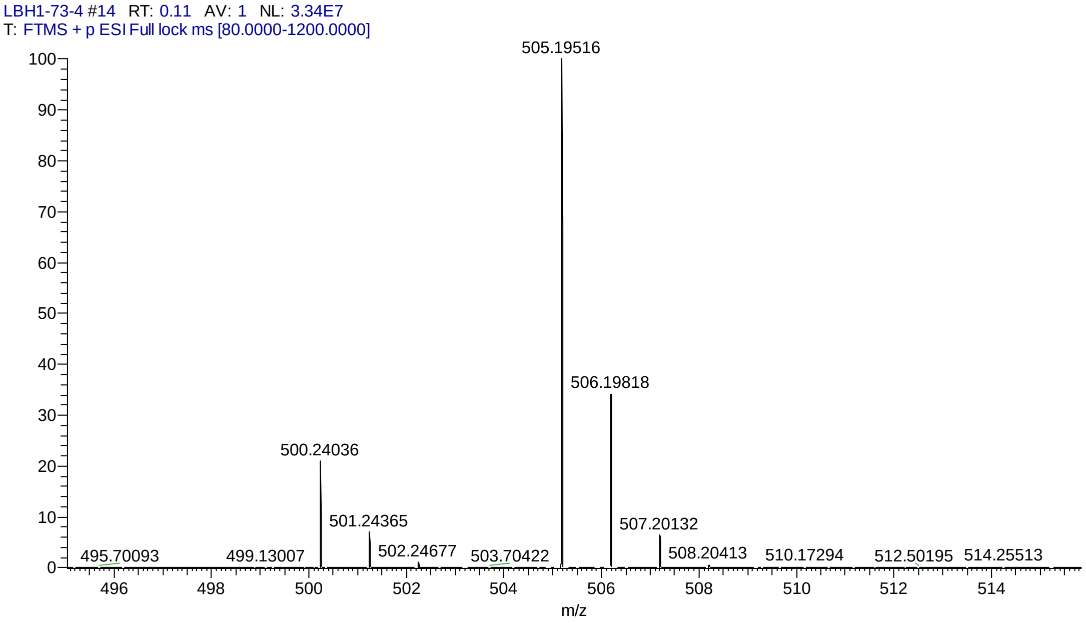

Figure S24. HR-ESI-MS spectrum of **2c.**

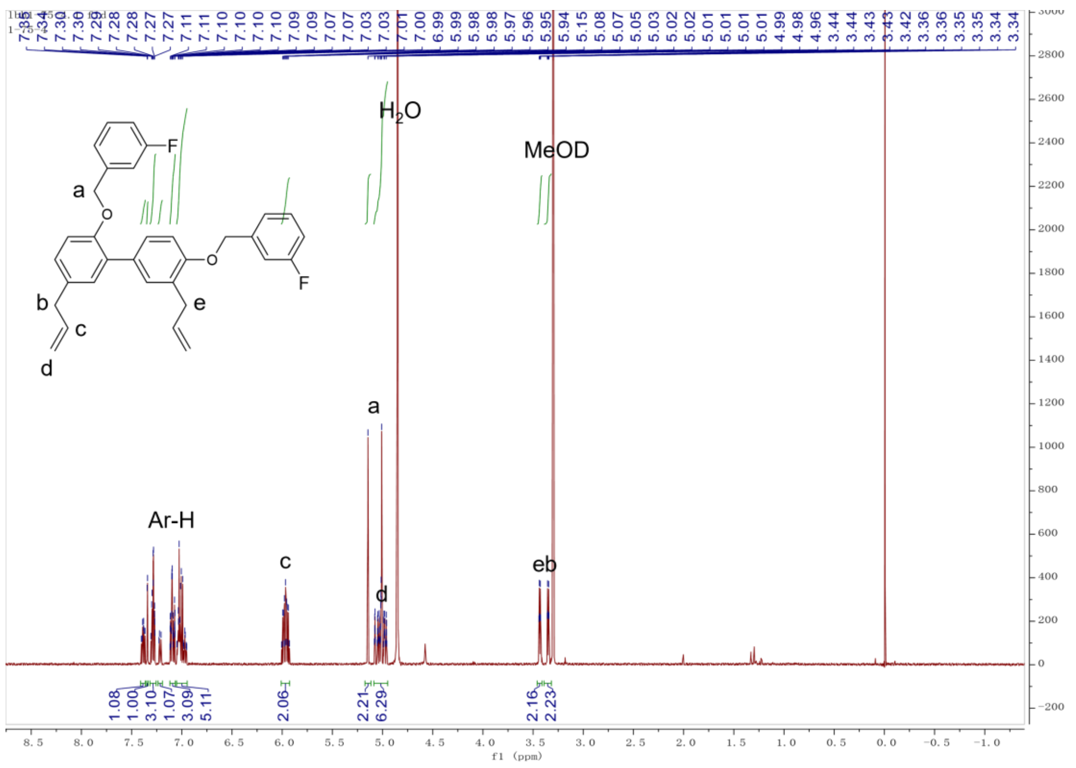

Figure S25. ^{1}H-NMR (600 MHz, CD_3OD) spectrum of **3c**.

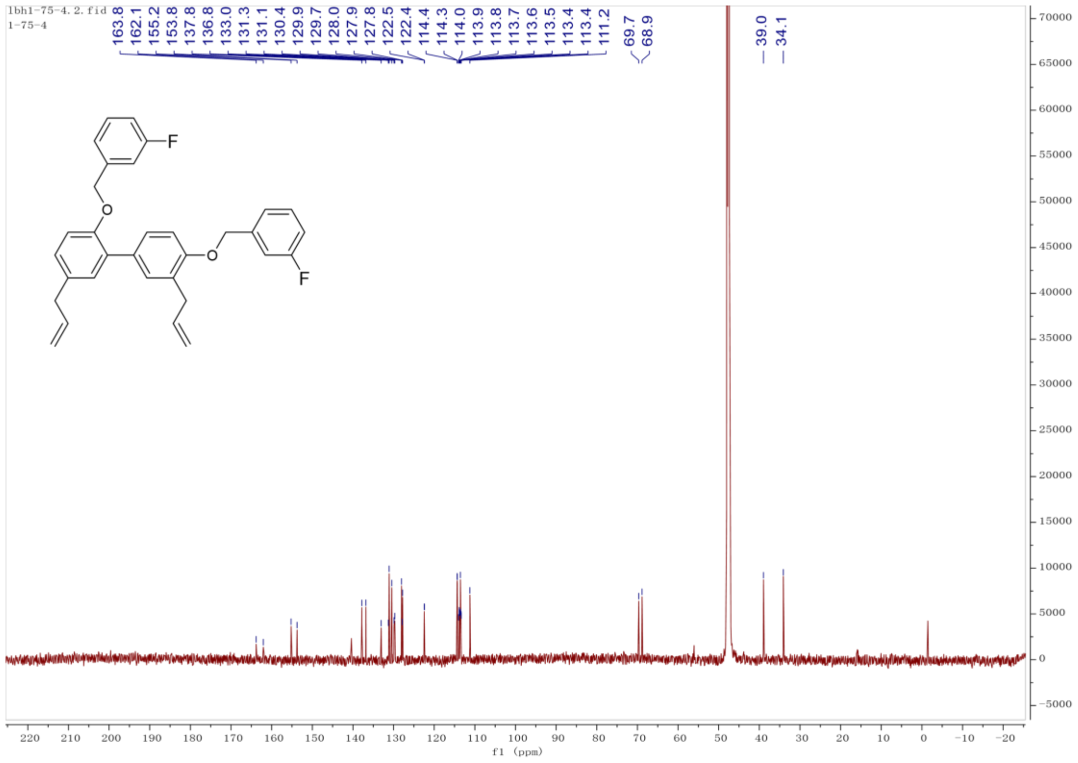

Figure S26. ^{13}C-NMR (150 MHz, CD_3OD) spectrum of **3c.**

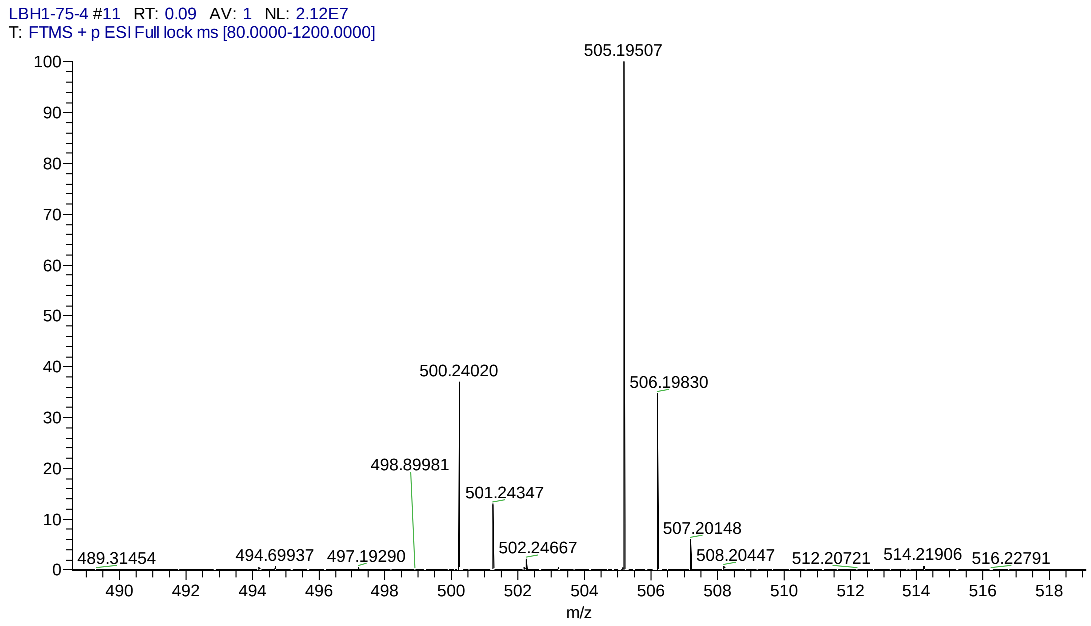

Figure S27. HR-ESI-MS spectrum of **3c.**

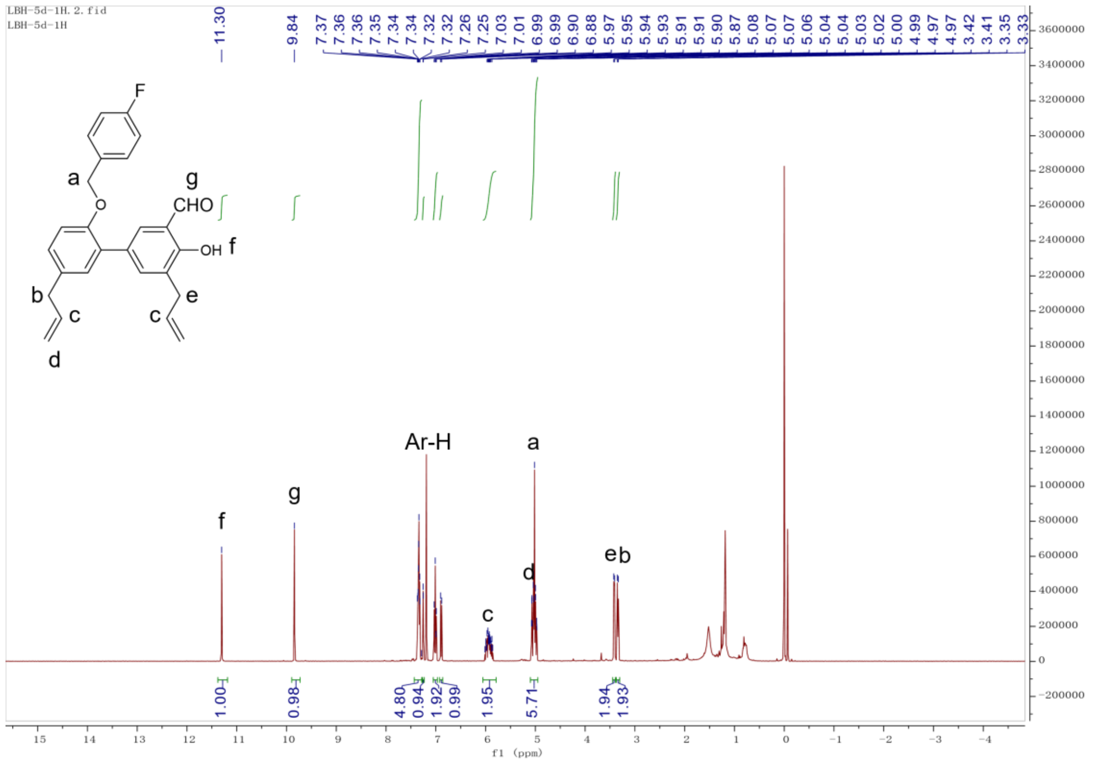

Figure S28. ^{1}H-NMR (400 MHz, $CDCl_3$) spectrum of **1d**.

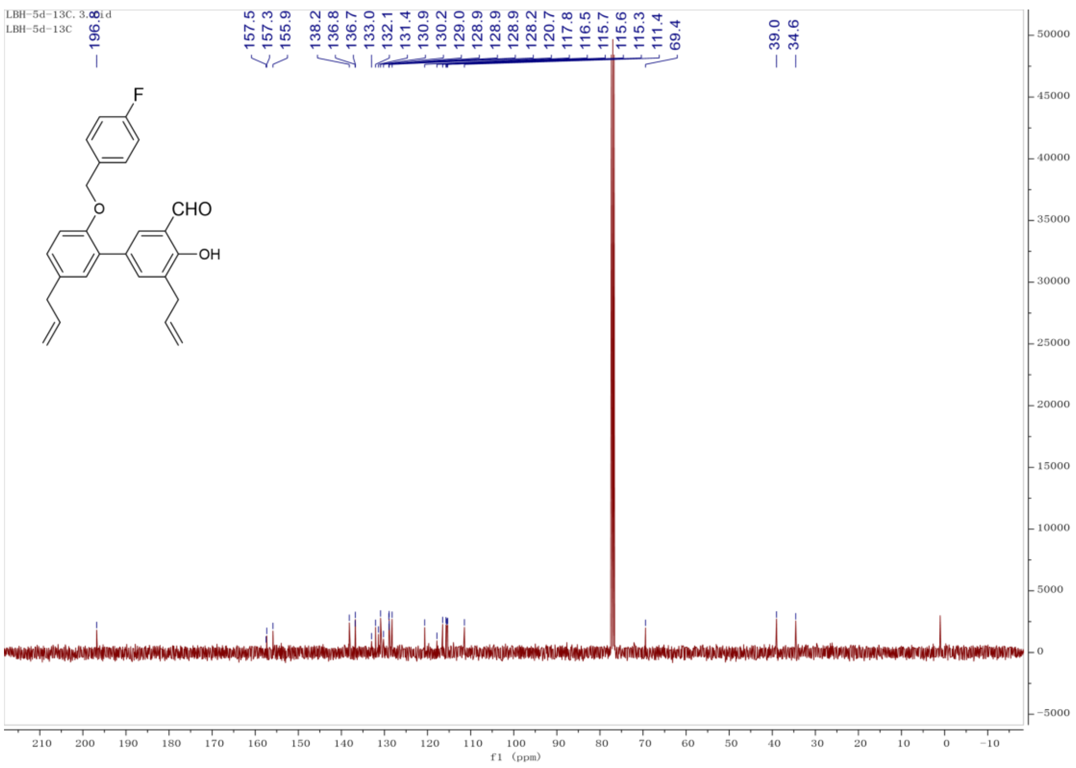

Figure S29. ^{13}C-NMR (100 MHz, $CDCl_3$) spectrum of **1d.**

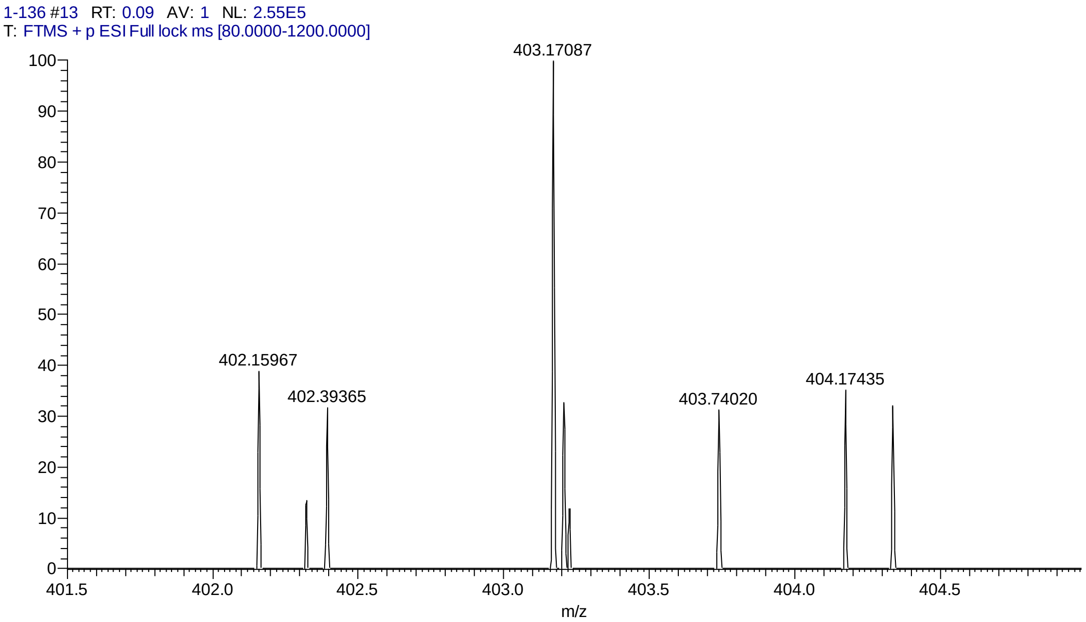

Figure S30. HR-ESI-MS spectrum of **1d.**

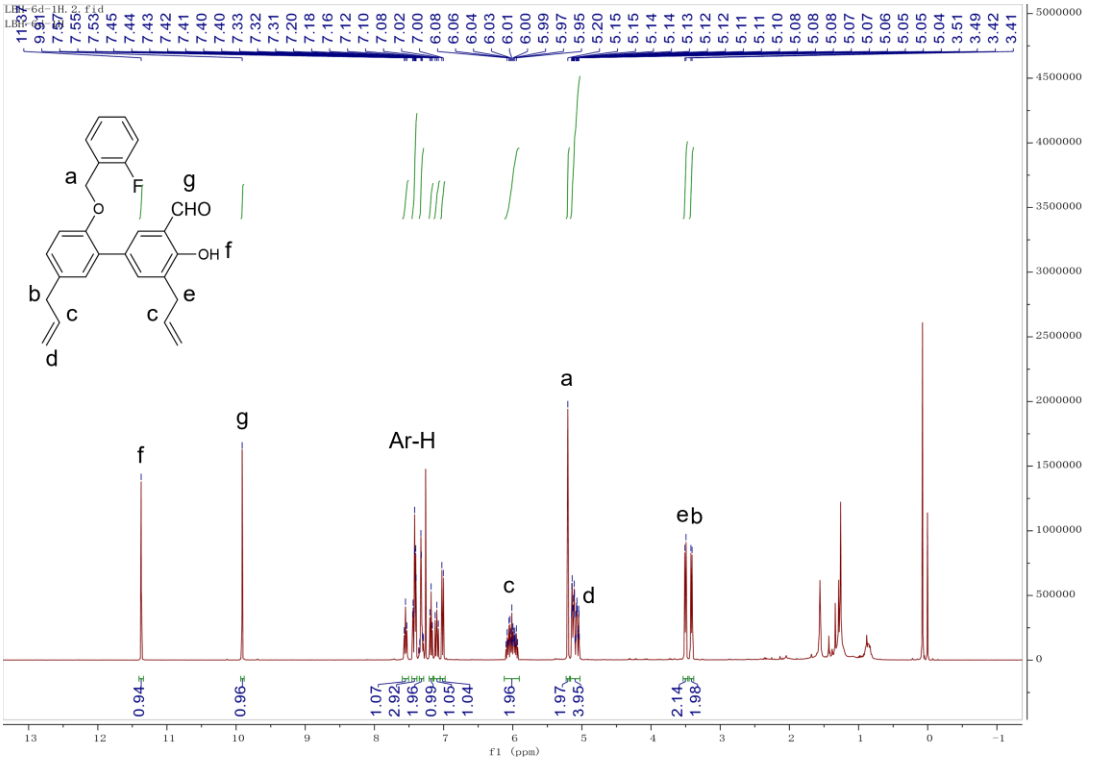

Figure S31. ^{1}H-NMR (400 MHz, $CDCl_3$) spectrum of **2d**.

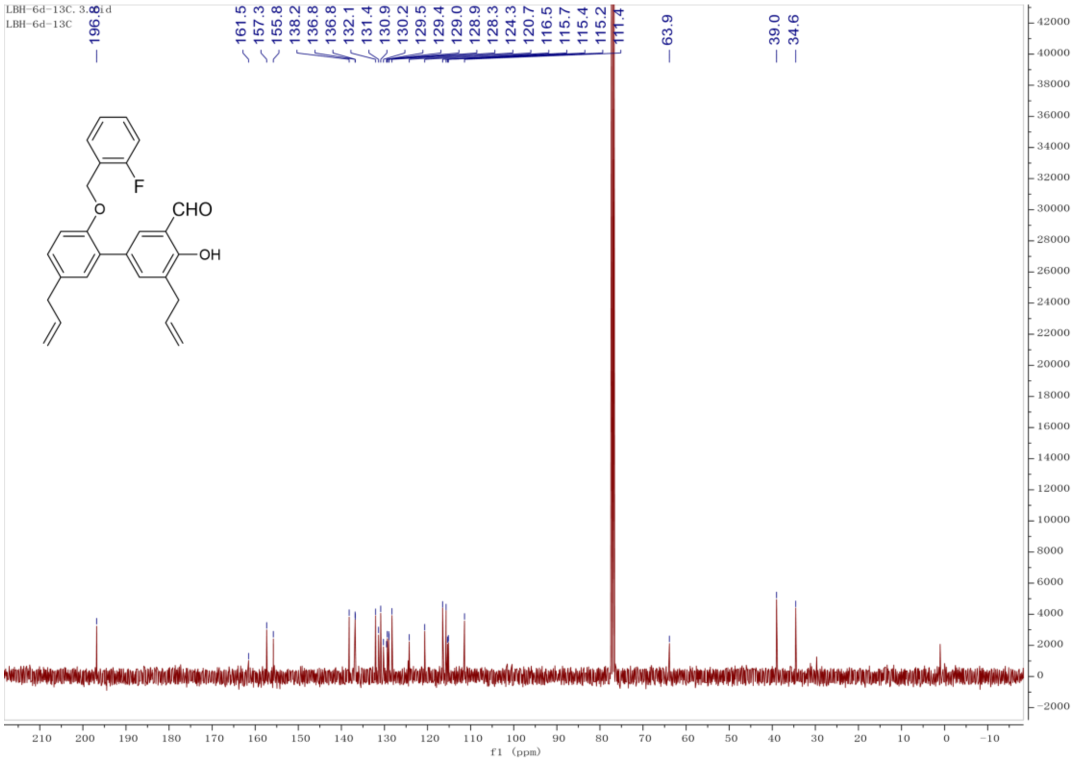

Figure S32. ^{13}C-NMR (100 MHz, $CDCl_3$) spectrum of **2d.**

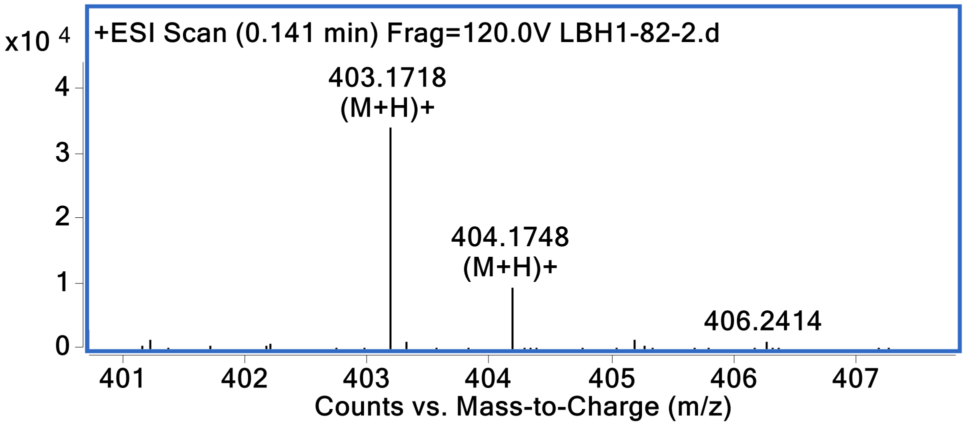

Figure S33. HR-ESI-MS spectrum of **2d.**

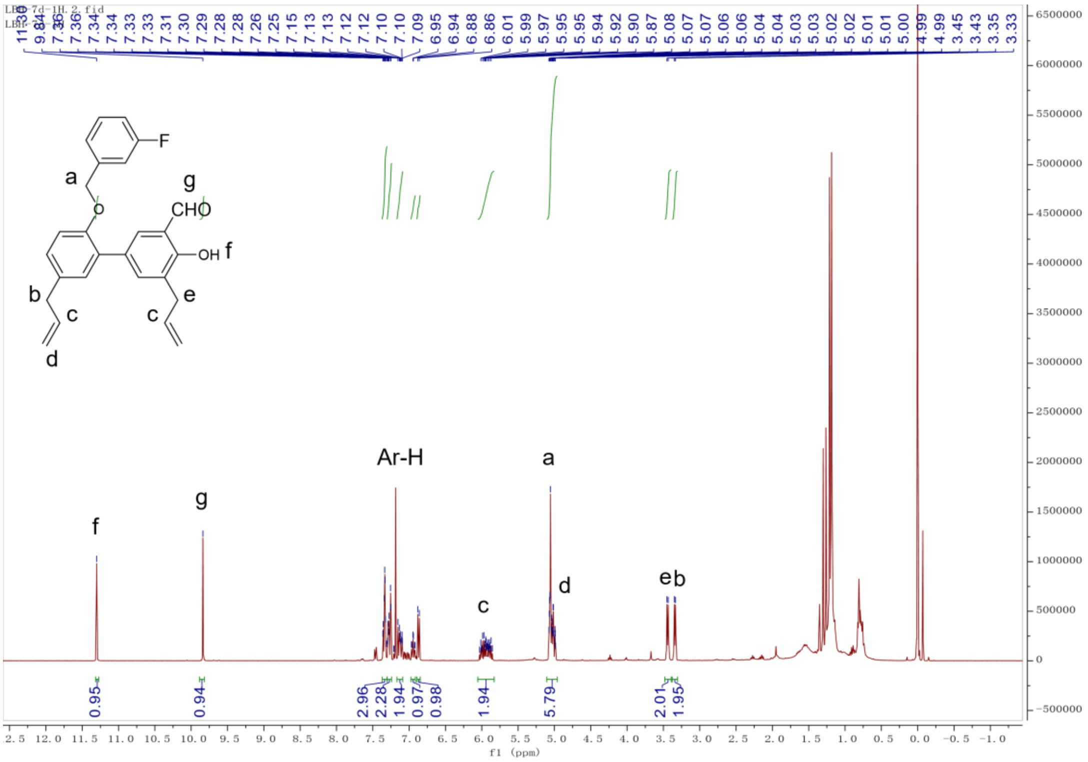

Figure S34. ^{1}H-NMR (400 MHz, $CDCl_3$) spectrum of **3d**.

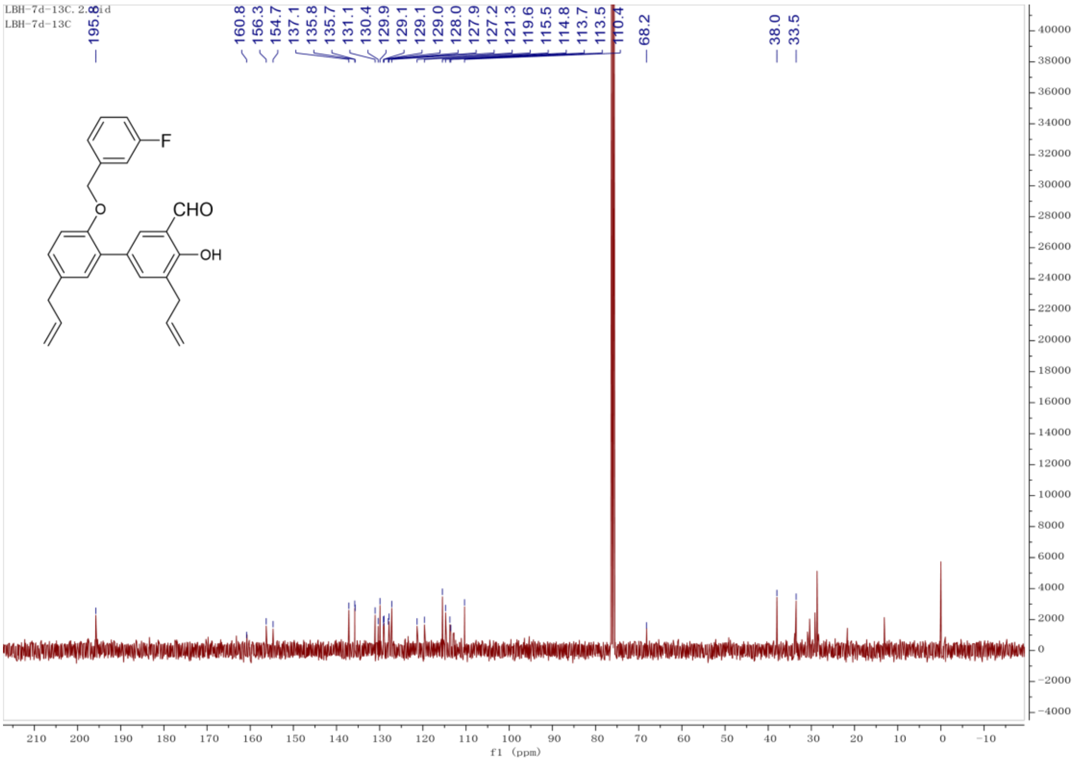

Figure S35. ^{13}C-NMR (100 MHz, $CDCl_3$) spectrum of **3d.**

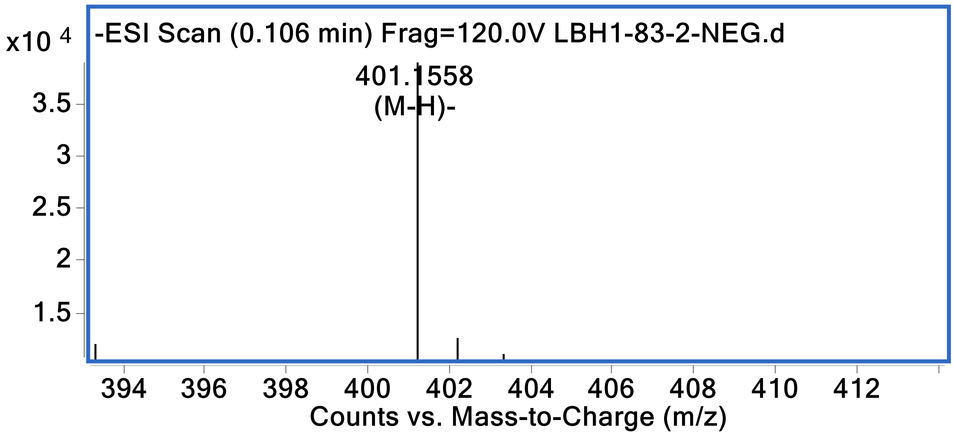

Figure S36. HR-ESI-MS spectrum of **3d.**

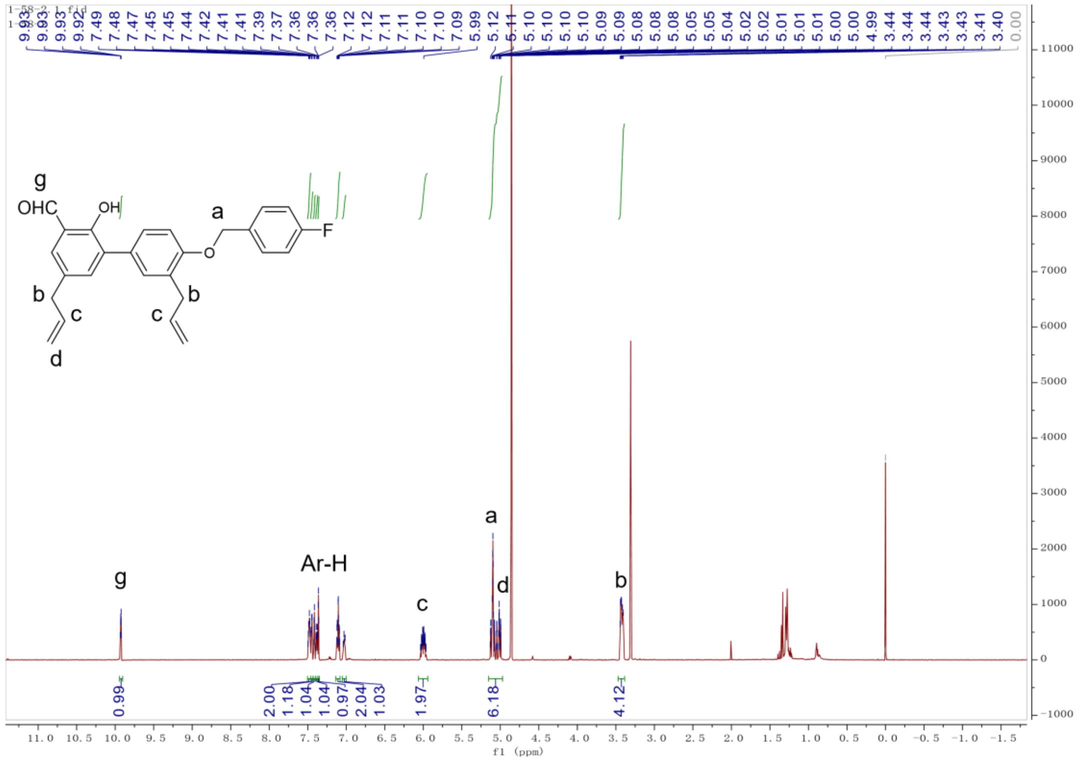

Figure S37. ^{1}H-NMR (600 MHz, CD_3OD) spectrum of **1e**.

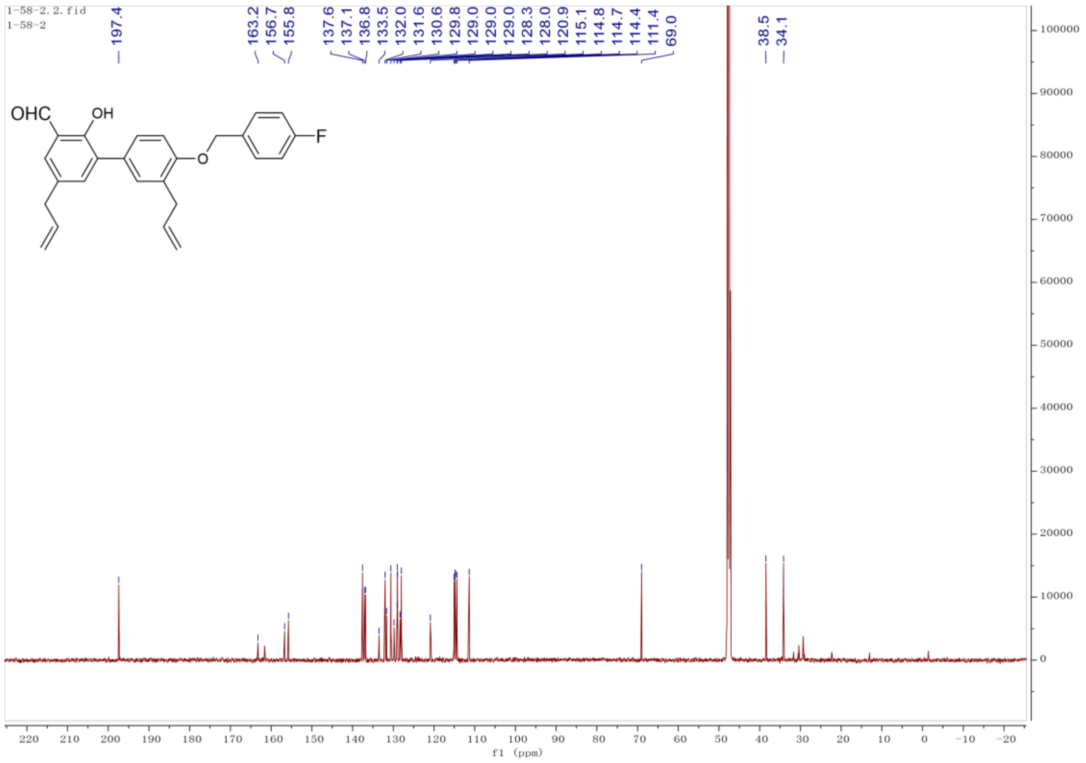

Figure S38. ^{13}C-NMR (150 MHz, CD_3OD) spectrum of **1e.**

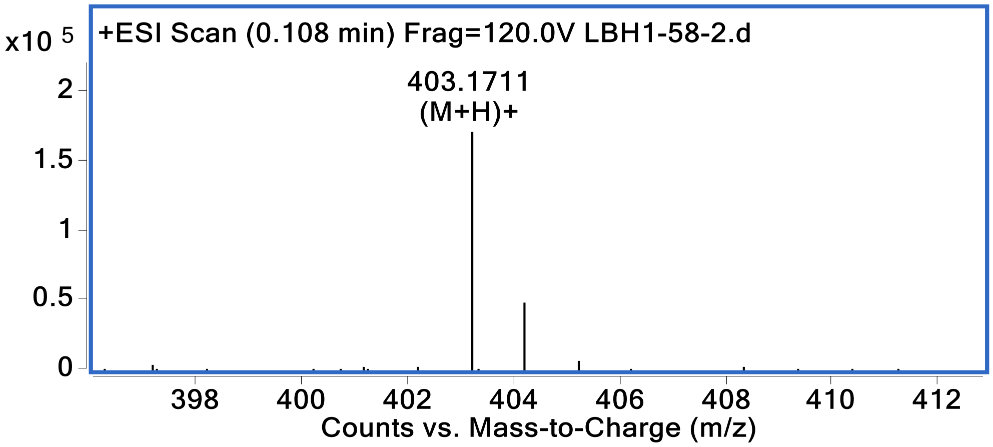

Figure S39. HR-ESI-MS spectrum of **1e.**

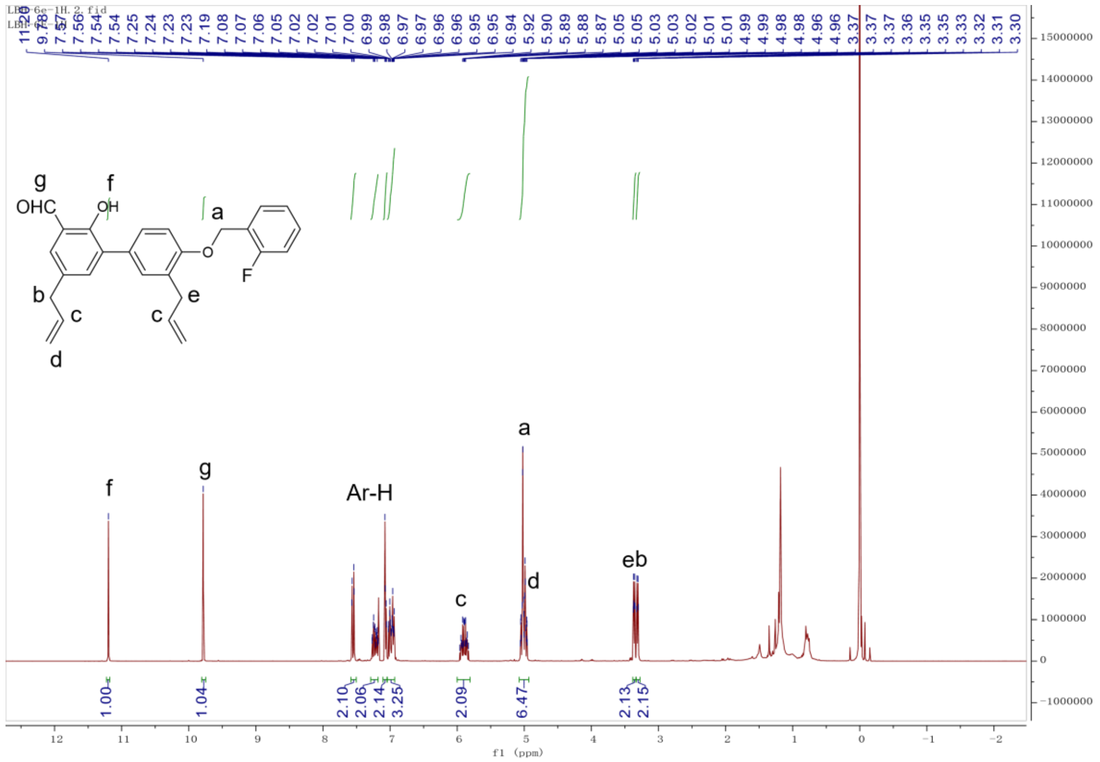

Figure S40. ^{1}H-NMR (400 MHz, $CDCl_3$) spectrum of **2e**.

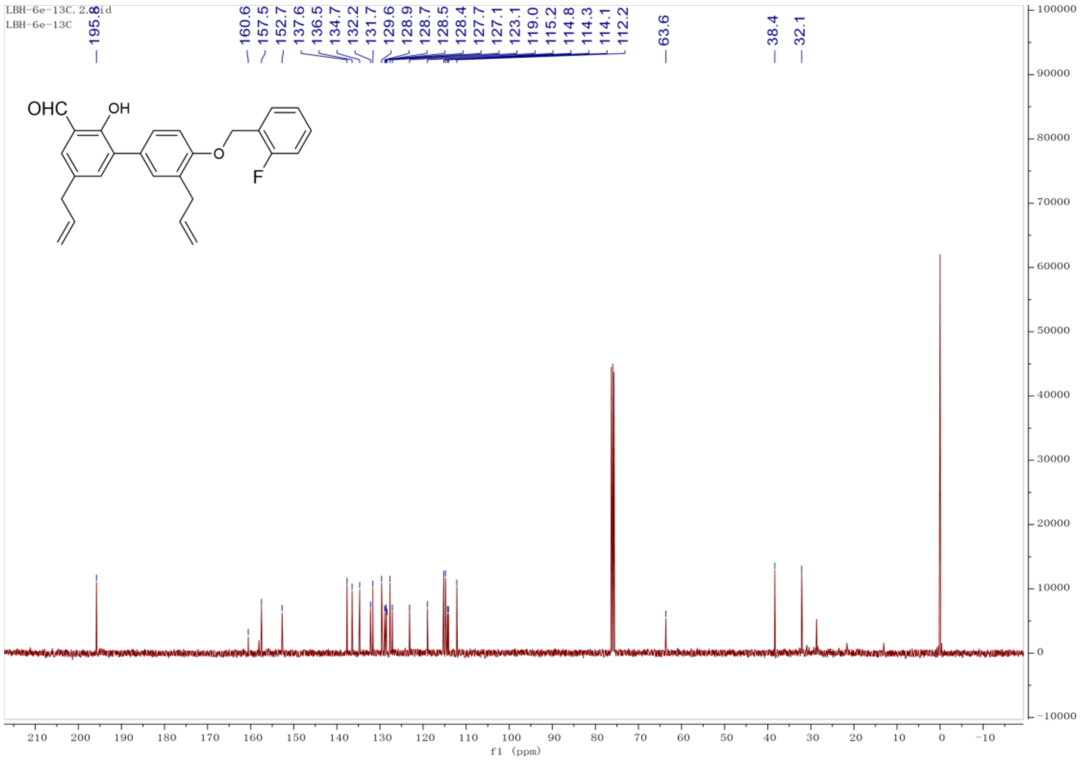

Figure S41. ^{13}C-NMR (100 MHz, $CDCl_3$) spectrum of **2e.**

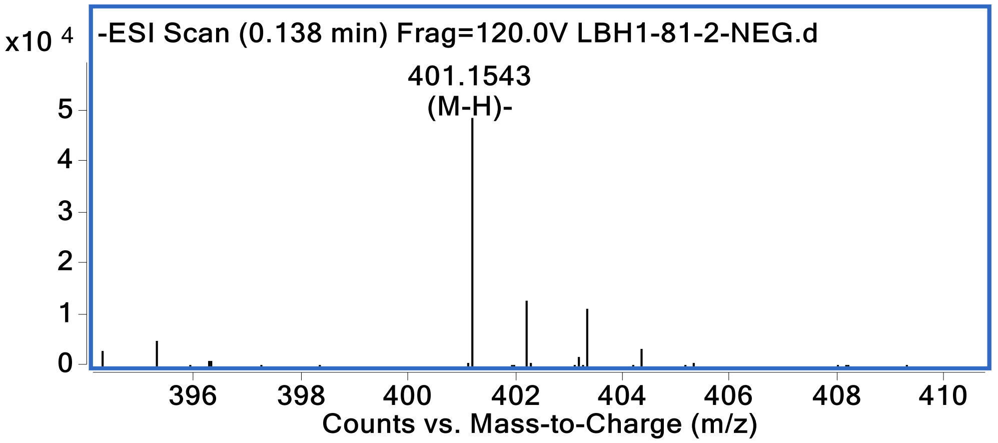

Figure S42. HR-ESI-MS spectrum of **2e.**

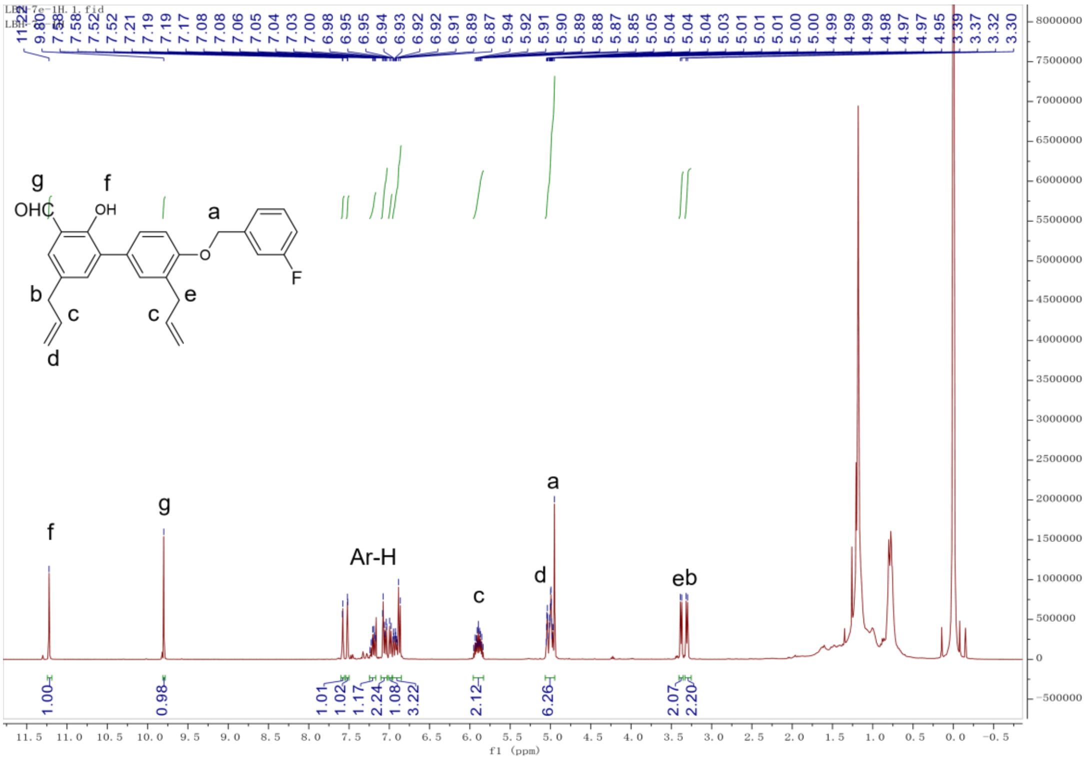

Figure S43. ^{1}H-NMR (400 MHz, $CDCl_3$) spectrum of **3e**.

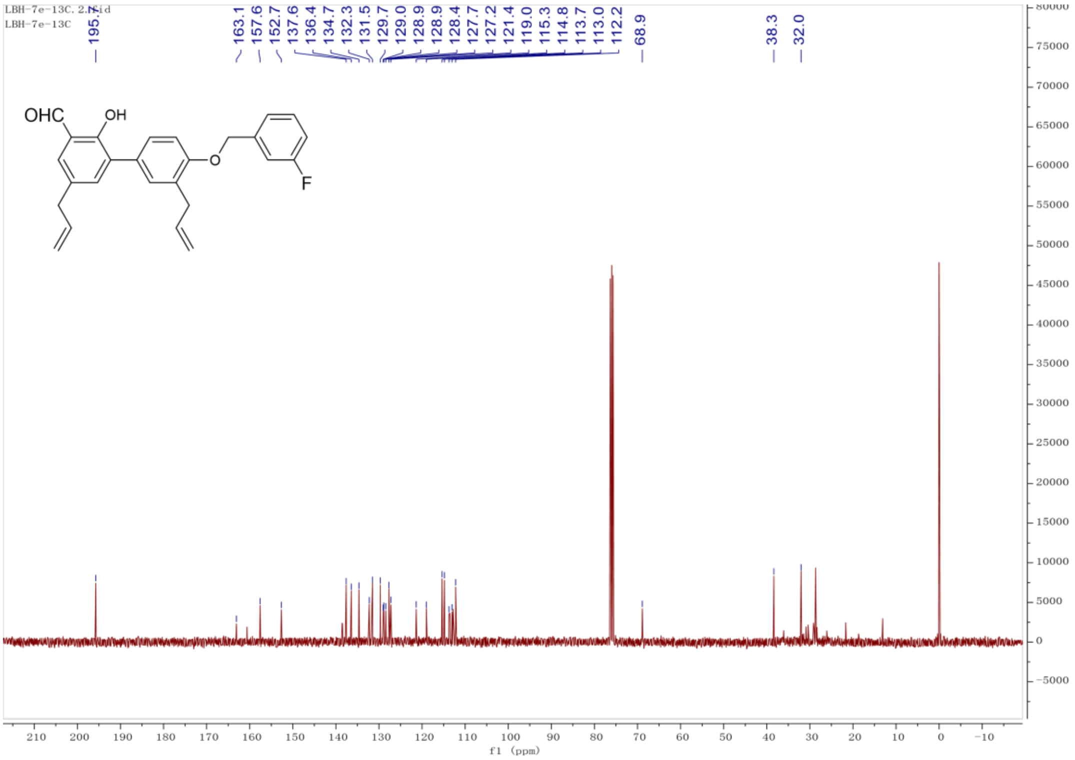

Figure S44. ^{13}C-NMR (100 MHz, $CDCl_3$) spectrum of **3e.**

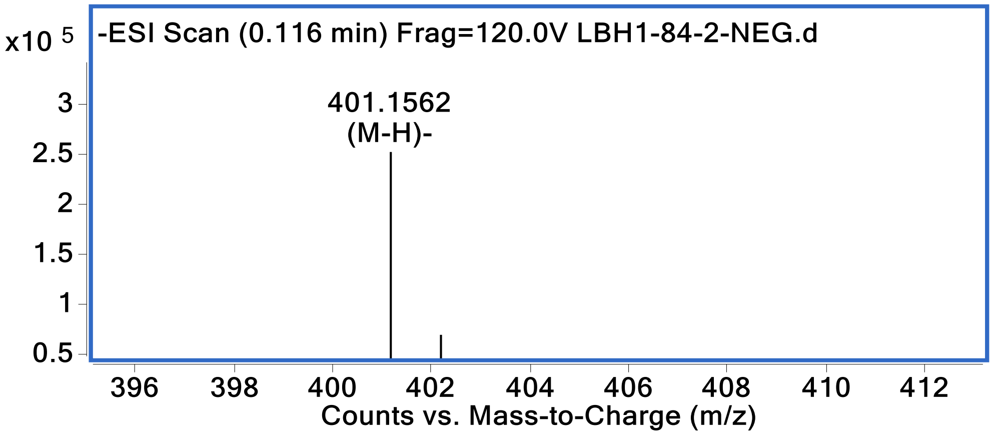

Figure S45. HR-ESI-MS spectrum of **3e.**

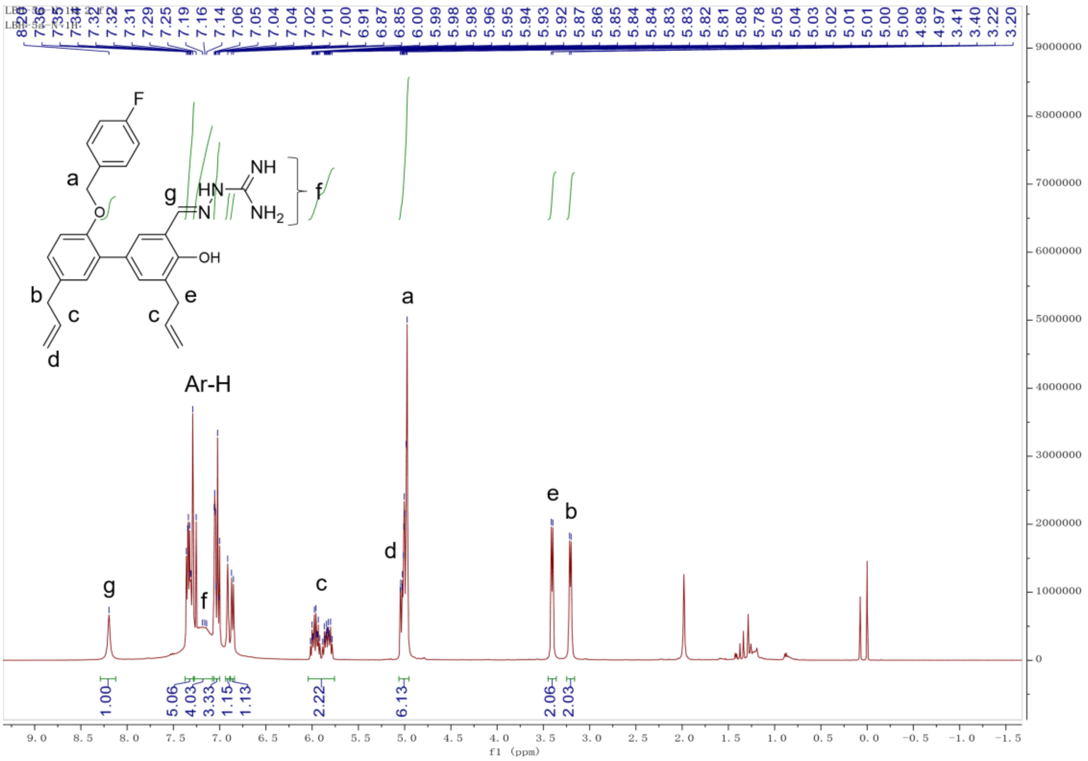

Figure S46. ^{1}H-NMR (400 MHz, $CDCl_3$) spectrum of **1f**.

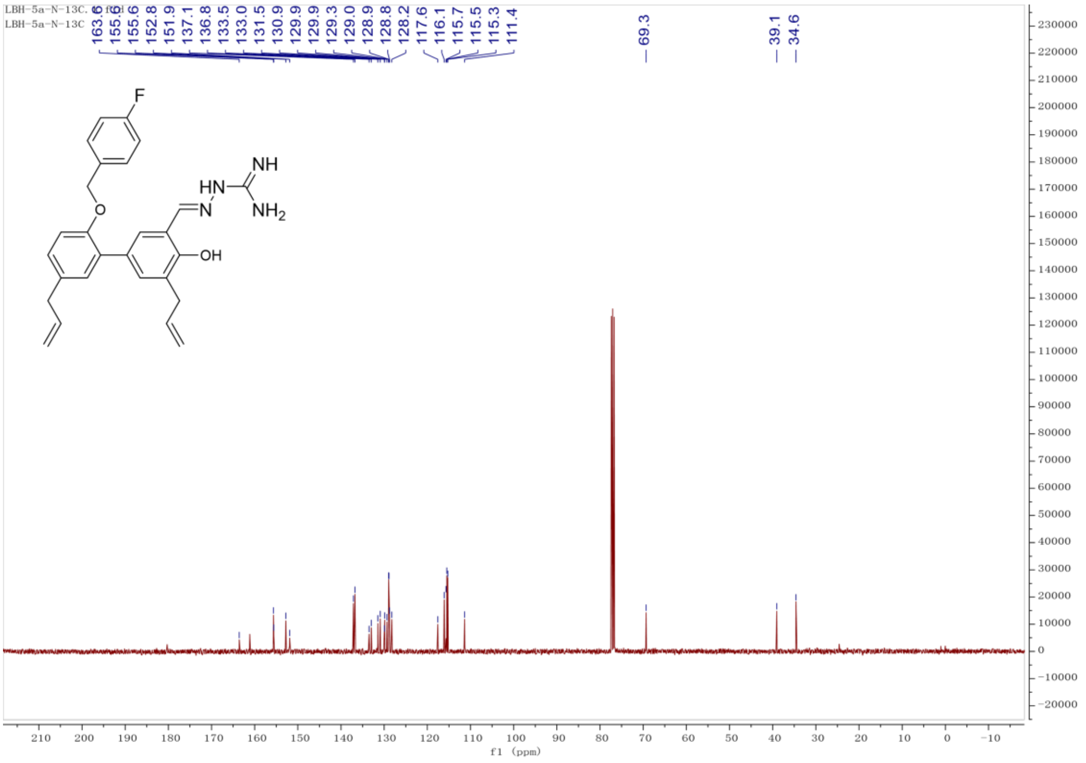

Figure S47. ^{13}C-NMR (100 MHz, $CDCl_3$) spectrum of **1f.**

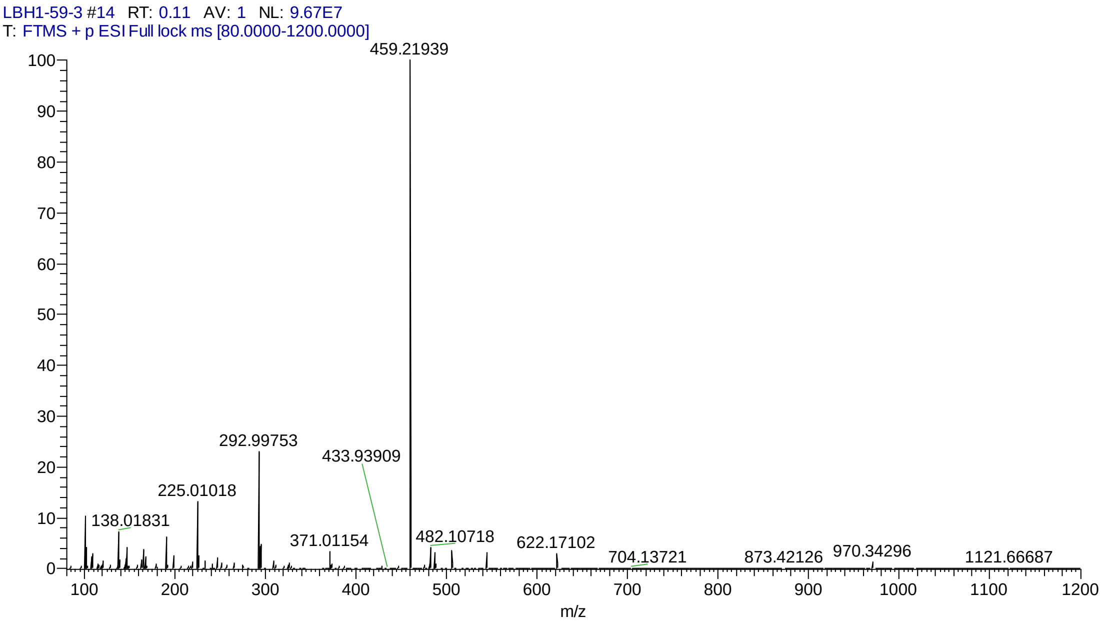

Figure S48. HR-ESI-MS spectrum of **1f.**

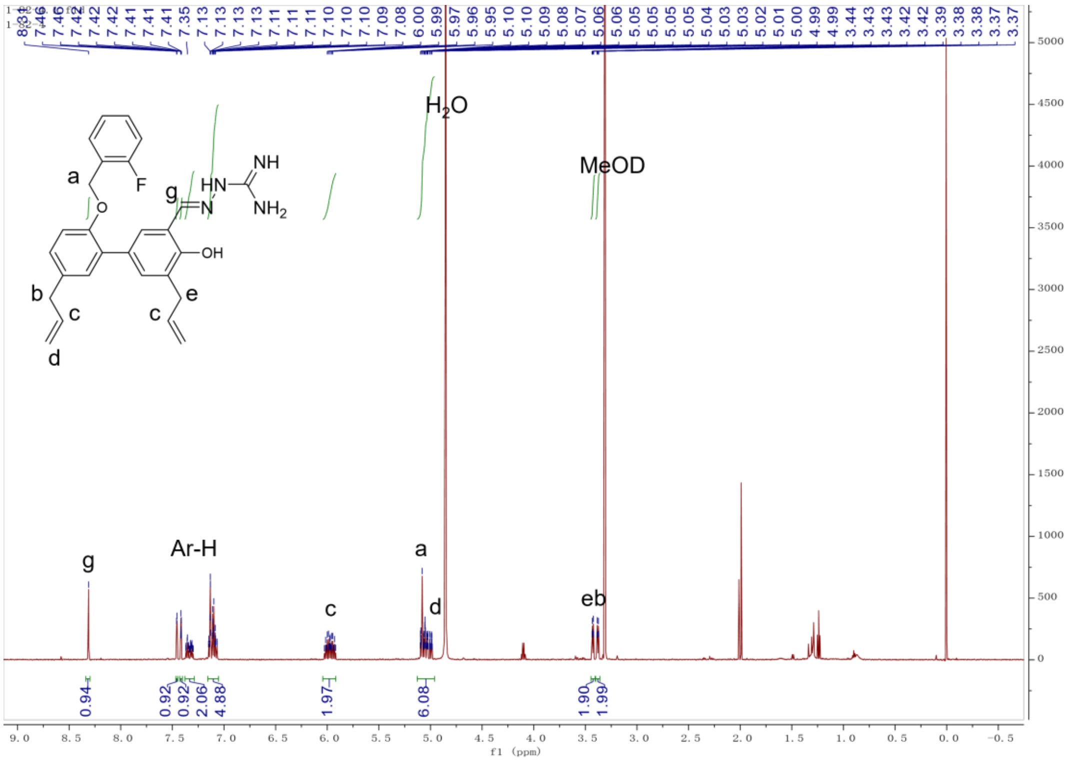

Figure S49. ^{1}H-NMR (600 MHz, CD_3OD) spectrum of **2f**.

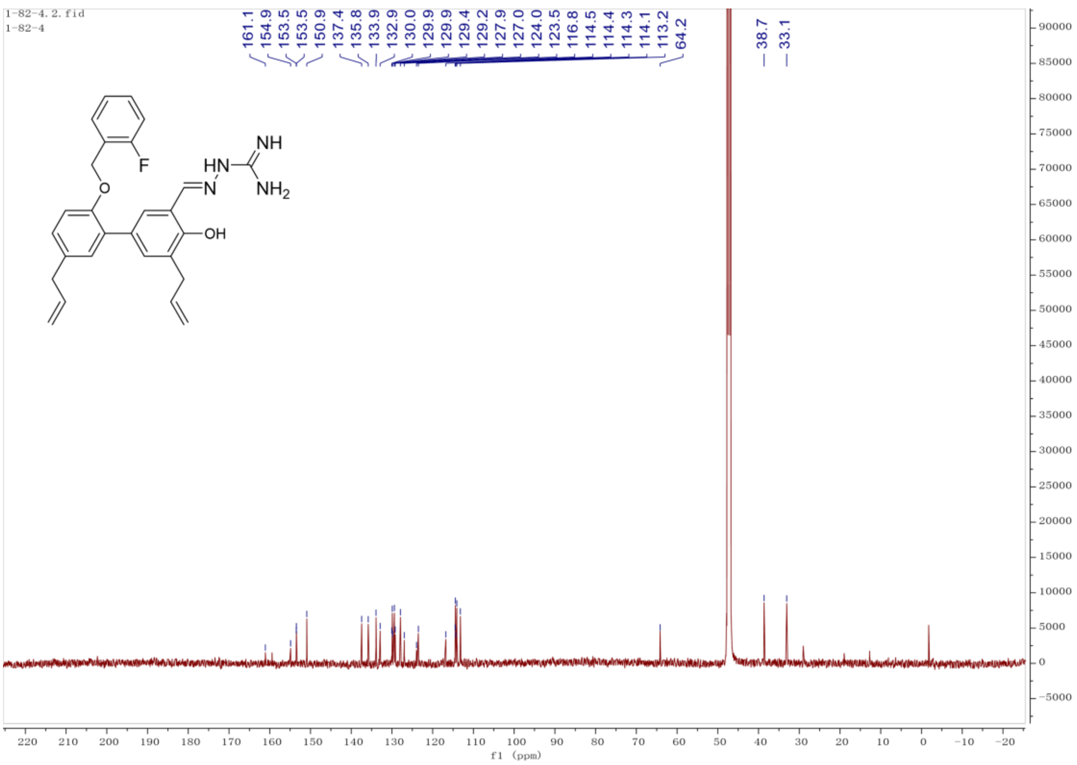

Figure S50. ^{13}C-NMR (150 MHz, CD_3OD) spectrum of **2f.**

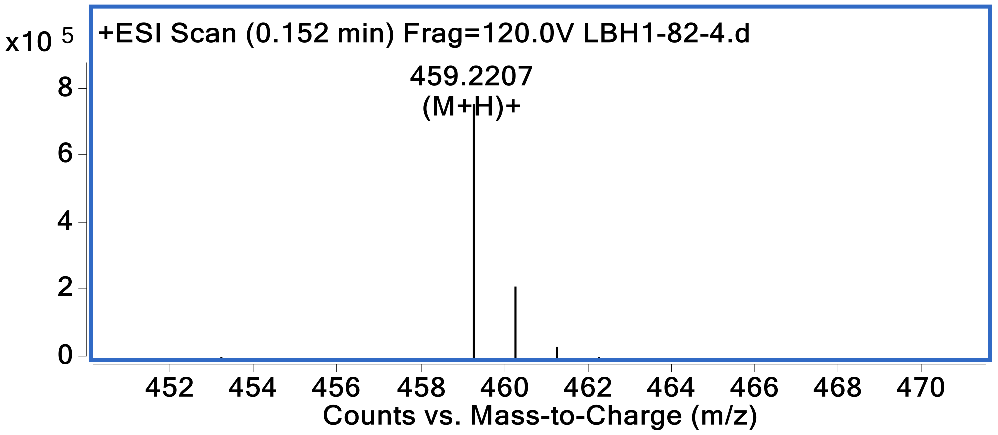

Figure S51. HR-ESI-MS spectrum of **2f.**

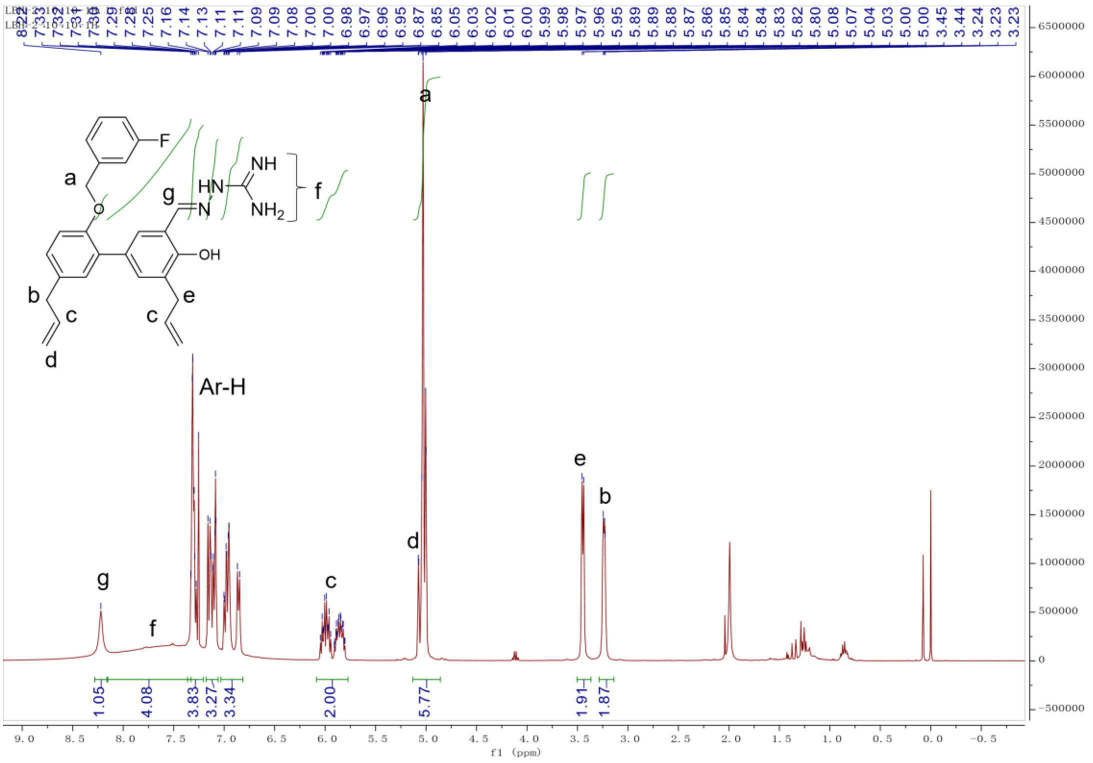

Figure S52. ^{1}H-NMR (400 MHz, $CDCl_3$) spectrum of **3f**.

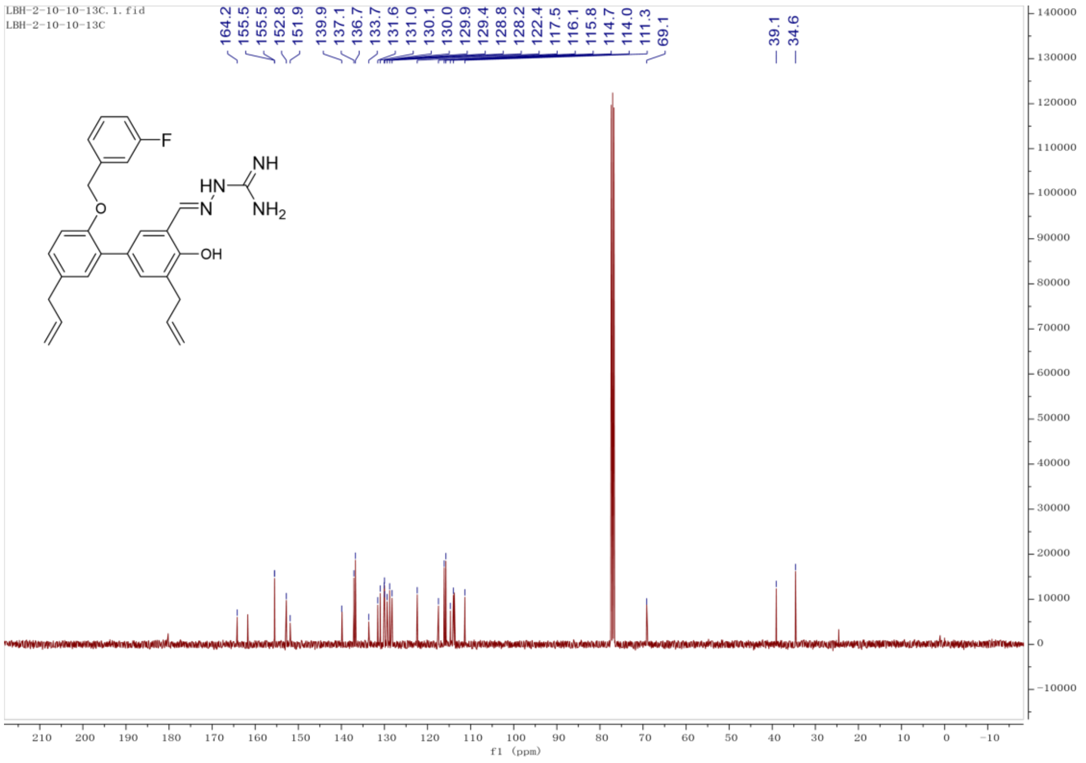

Figure S53. ^{13}C-NMR (100 MHz, $CDCl_3$) spectrum of **3f.**

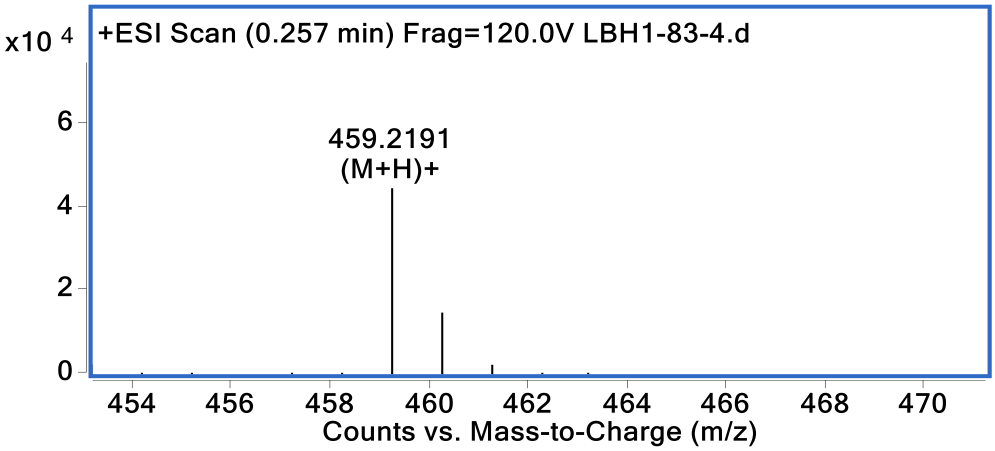

Figure S54. HR-ESI-MS spectrum of **3f.**

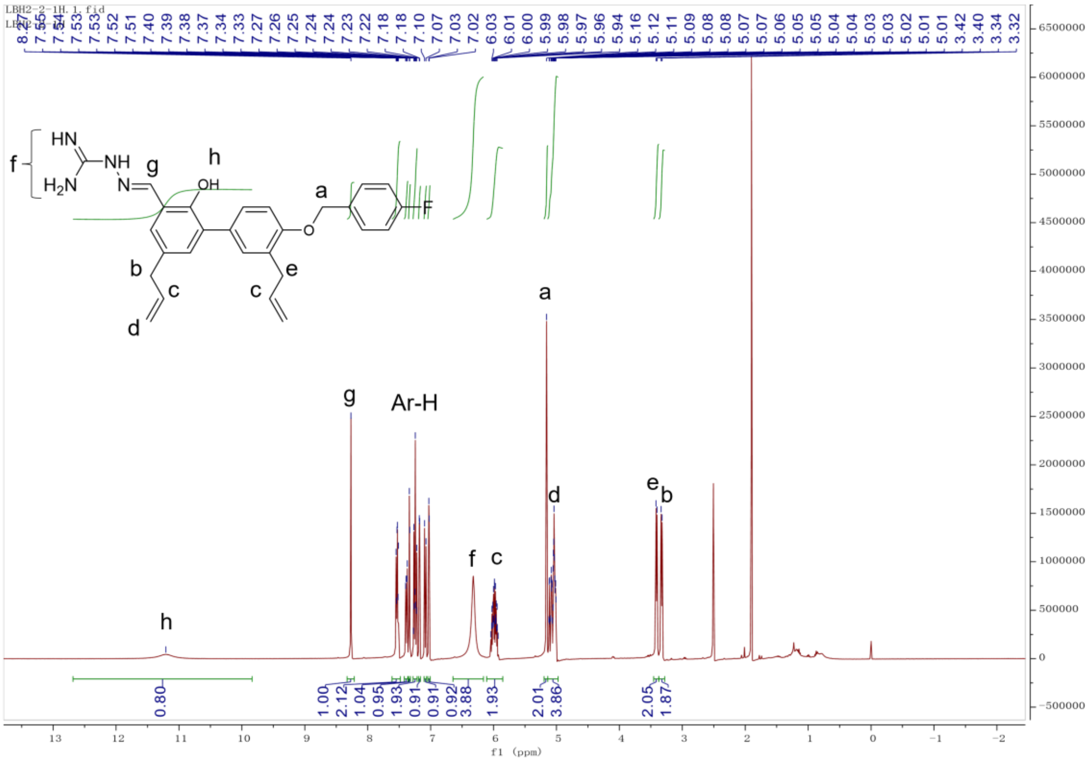

Figure S55. ^{1}H-NMR (400 MHz, DMSO-d_6) spectrum of **1g**.

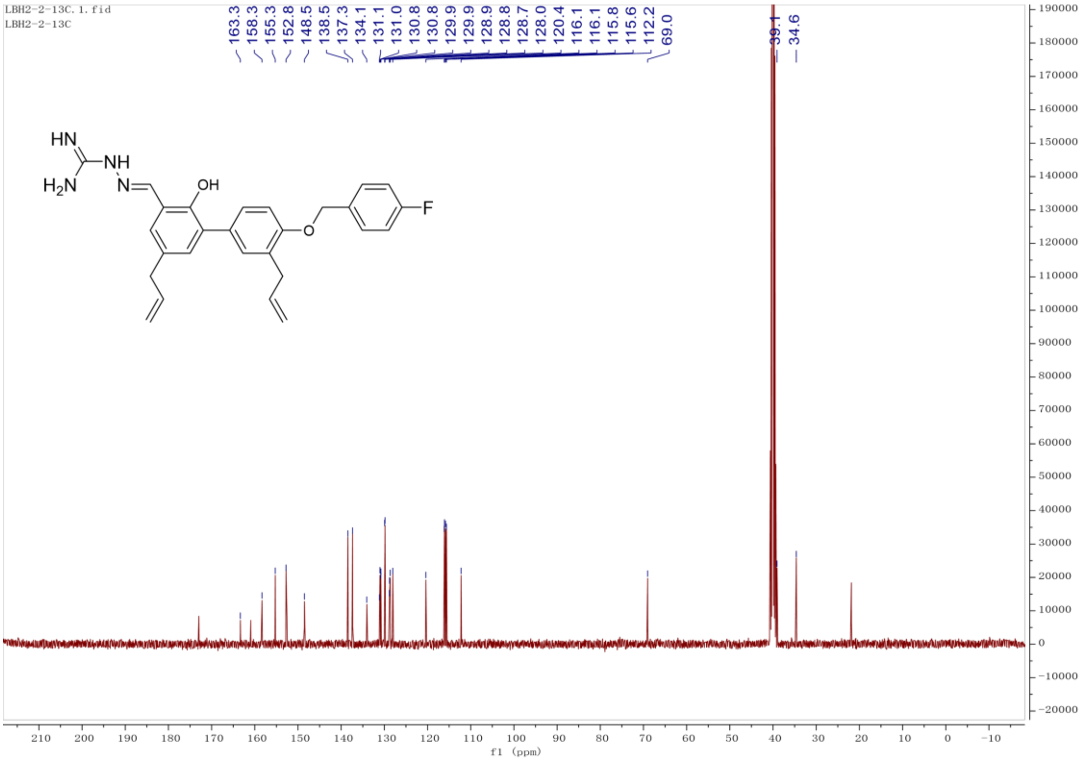

Figure S56. ^{13}C-NMR (100 MHz, DMSO-d_6) spectrum of **1g.**

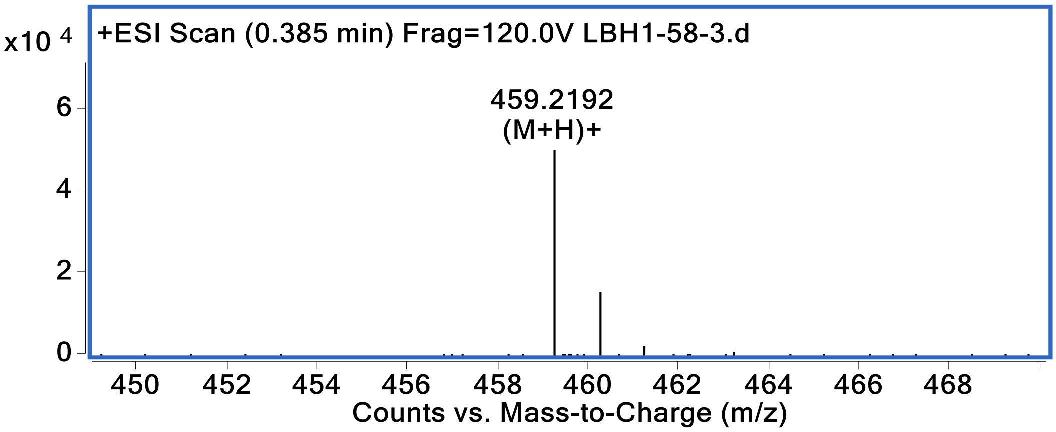

Figure S57. HR-ESI-MS spectrum of **1g.**

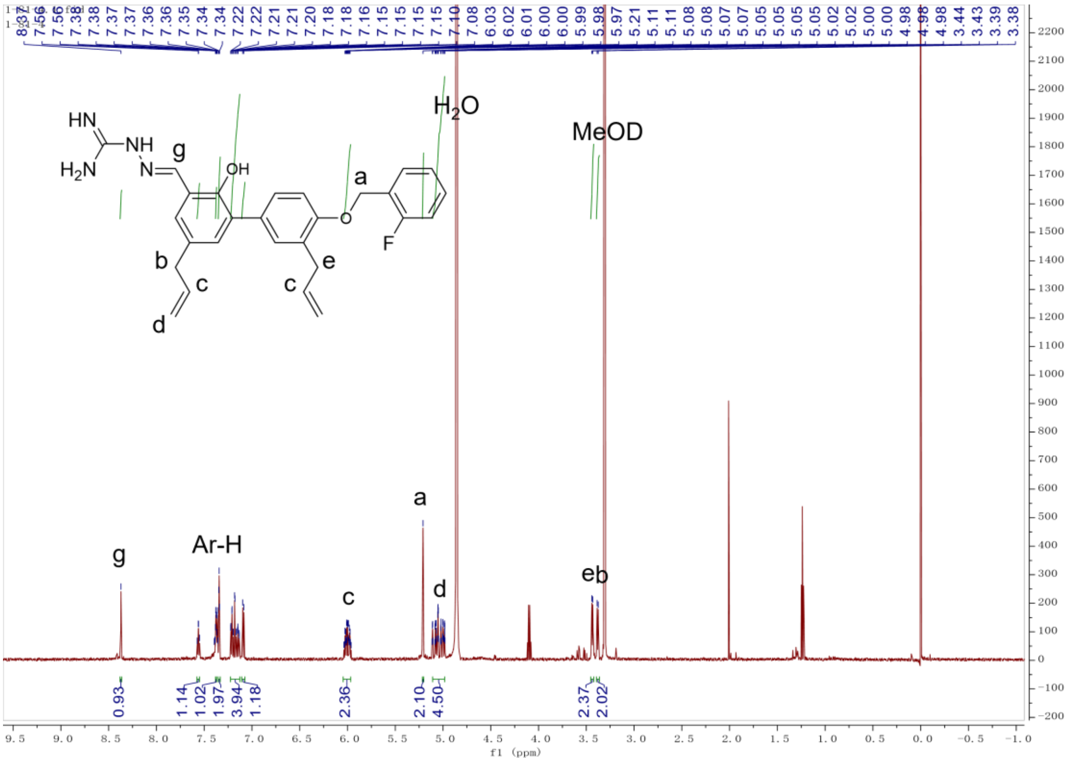

Figure S58. ^{1}H-NMR (600 MHz, CD_3OD) spectrum of **2g**.

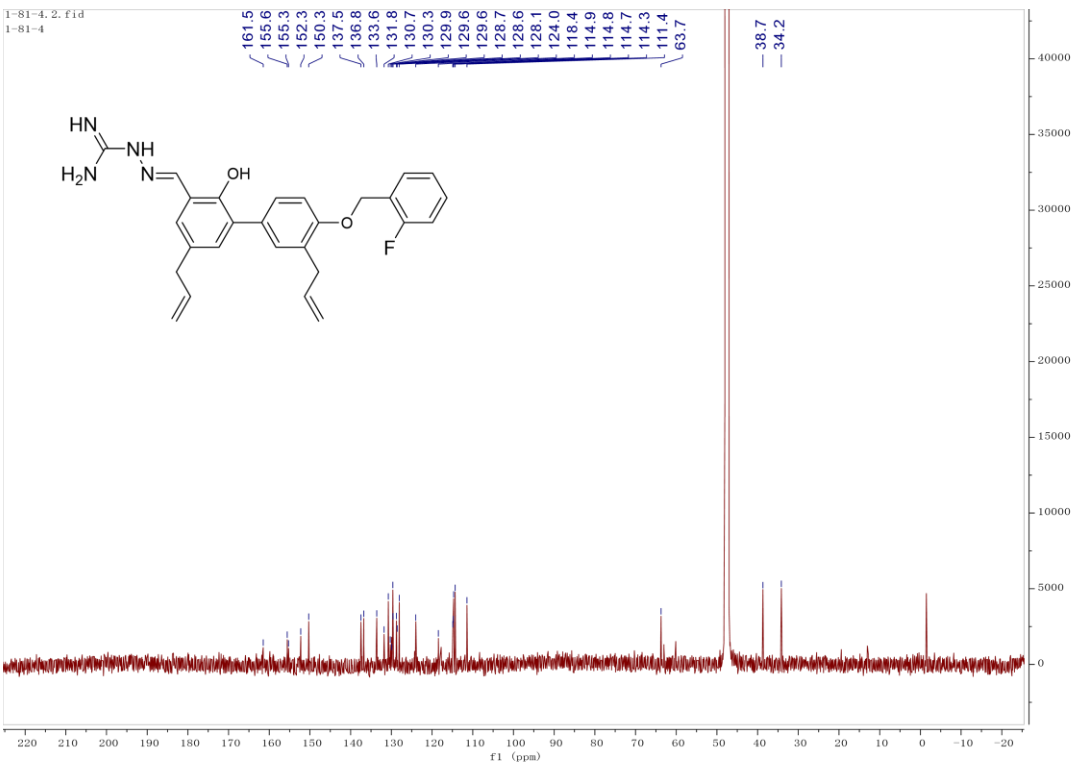

Figure S59. ^{13}C-NMR (150 MHz, CD_3OD) spectrum of **2g.**

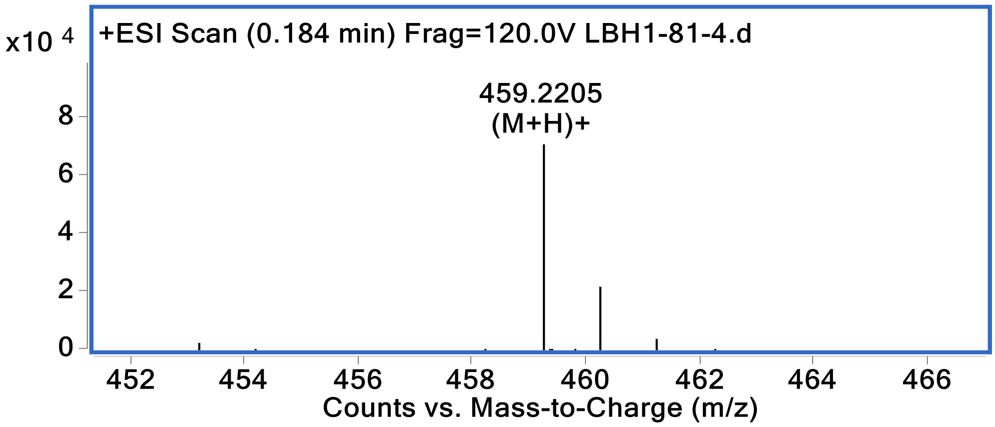

Figure S60. HR-ESI-MS spectrum of **2g.**

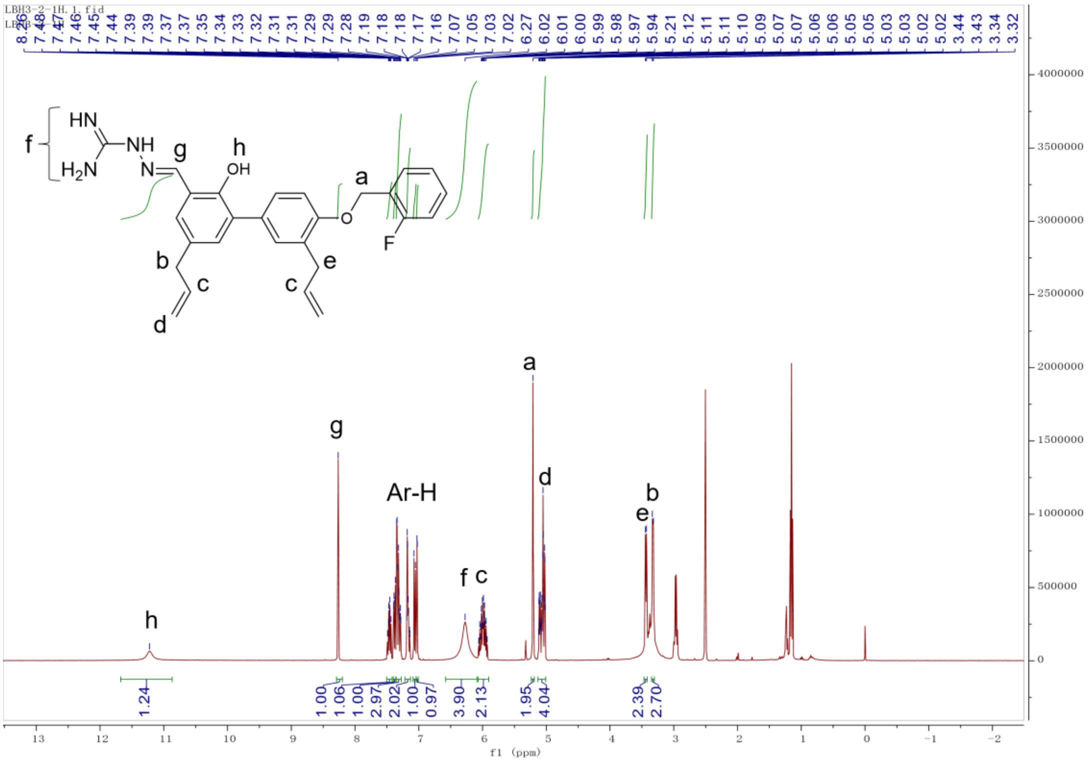

Figure S61. ^{1}H-NMR (400 MHz, DMSO-d_6) spectrum of **3g**.

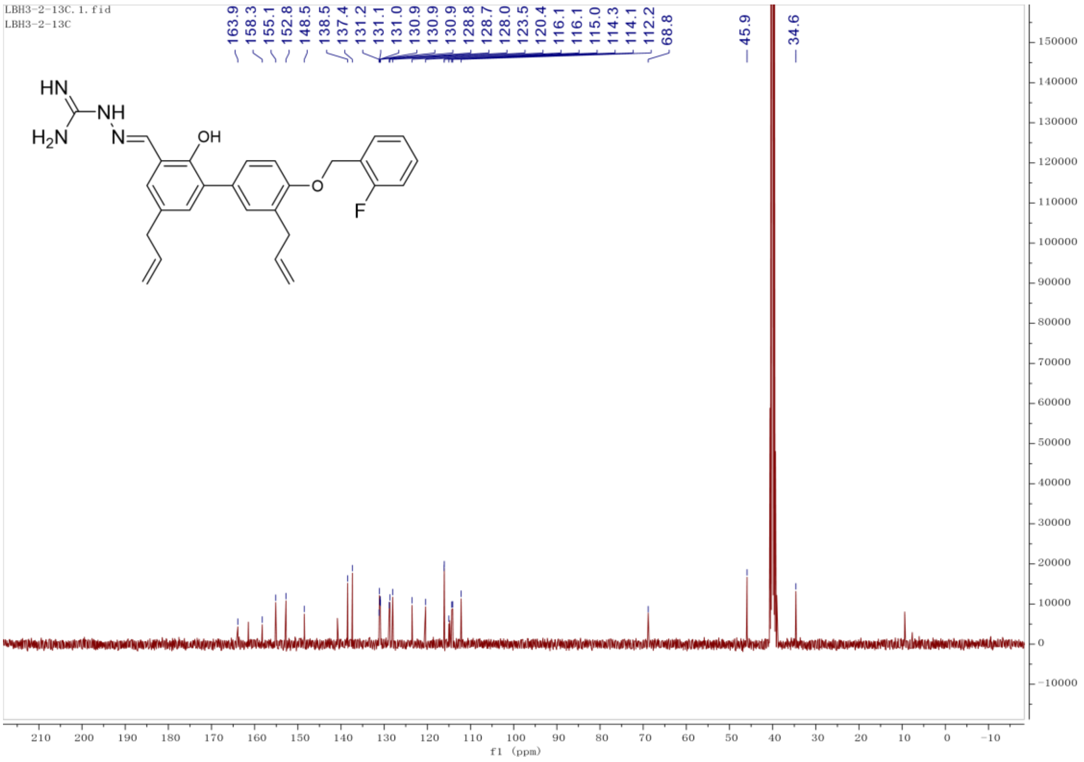

Figure S62. ^{13}C-NMR (100 MHz, DMSO-d_6) spectrum of **3g.**

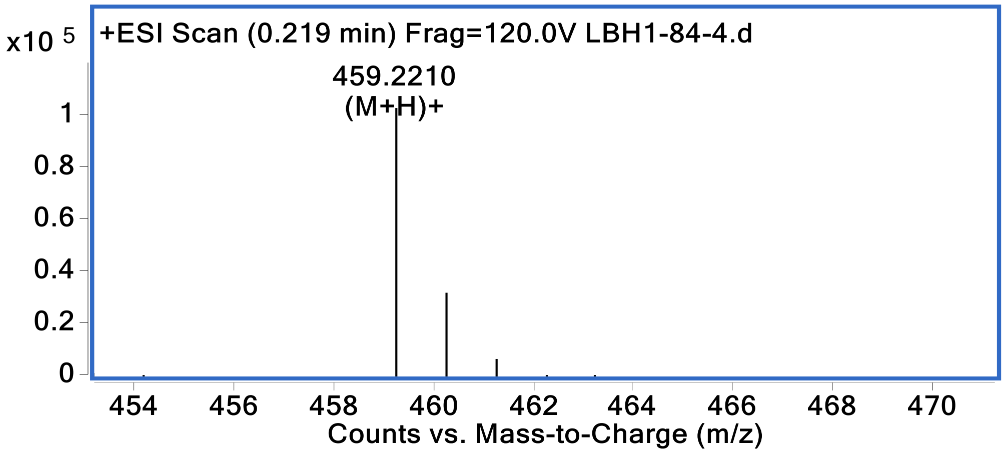

Figure S63. HR-ESI-MS spectrum of **3g.**
